# Thermodynamics and Kinetics of Drug-Target Binding by Molecular Simulation

**DOI:** 10.1021/acs.chemrev.0c00534

**Published:** 2020-10-02

**Authors:** Sergio Decherchi, Andrea Cavalli

**Affiliations:** † Computational and Chemical Biology, Fondazione Istituto Italiano di Tecnologia, 16163 Genoa, Italy; ‡ Department of Pharmacy and Biotechnology, University of Bologna, 40126 Bologna, Italy

## Abstract

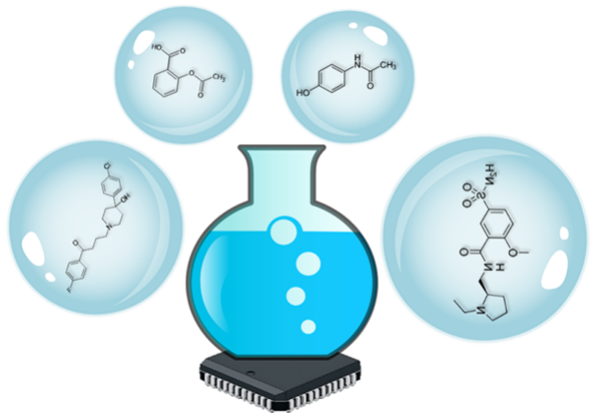

Computational studies play an increasingly important role in chemistry and biophysics, mainly thanks to improvements in hardware and algorithms. In drug discovery and development, computational studies can reduce the costs and risks of bringing a new medicine to market. Computational simulations are mainly used to optimize promising new compounds by estimating their binding affinity to proteins. This is challenging due to the complexity of the simulated system. To assess the present and future value of simulation for drug discovery, we review key applications of advanced methods for sampling complex free-energy landscapes at near nonergodicity conditions and for estimating the rate coefficients of very slow processes of pharmacological interest. We outline the statistical mechanics and computational background behind this research, including methods such as steered molecular dynamics and metadynamics. We review recent applications to pharmacology and drug discovery and discuss possible guidelines for the practitioner. Recent trends in machine learning are also briefly discussed. Thanks to the rapid development of methods for characterizing and quantifying rare events, simulation’s role in drug discovery is likely to expand, making it a valuable complement to experimental and clinical approaches.

## 1 Introduction

Medical treatments are becoming more effective and more widely available to the global population, driven in part by the introduction of new drugs to treat more conditions with fewer side effects. Sustaining this progress is a social, industrial, financial, and scientific challenge. Most current drugs are small organic molecules of natural or synthetic origin (molecular weight ≤ 500 Da). In comparison, biological macromolecules (e.g., antibodies) are a new frontier, biochemically, clinically, and in terms of computational investigations.^1−4^ For both kinds of drugs, their discovery and development requires massive investment by pharmaceutical companies, national governments, and other funding institutions. A huge basic research effort is required to fully understand the pathological processes of a given disease. Moreover, for each new drug approved for use in humans, an estimated 5,000–10,000 chemical compounds will undergo chemical and biological studies. Of these, approximately 250 will enter preclinical testing, and 5 will enter clinical trials.^5^ Bringing a new drug to market is estimated to take 10–15 years and cost up to 1.5–2.0 billion US dollars.^5^ Despite advances in technology and in our understanding of biological systems, it is still challenging to predict how a living organism will respond to a medicine. Yet accurate predictions can reduce the time and expense of drug discovery and development. In recent years, the dominant drug discovery paradigm has been to modulate a single biological target to tackle the symptoms and/or progression of a given disease.^6^ There is, however, growing evidence to suggest that, when seeking to understand a drug’s activities, one should also consider polypharmacological mechanisms of action.^6^ Most drug targets are proteins, whether cytosolic (e.g., kinases, proteases, and nuclear receptors) or membrane-embedded (e.g., G-protein-coupled receptors and ion channels). Biomolecules such as nucleic acids are also emerging as potential targets for treating several diseases. When a drug makes contact with its biological target, they establish a set of atomistic interactions, which are responsible for the drug’s potency and therapeutic effect. These atomistic interactions can be explored in great detail nowadays with experimental and computational tools. Ultimately, a drug can bind into different pockets, which are referred to as orthosteric or allosteric binding sites. Different biochemical responses can be triggered by binding to one pocket type or the other. This makes it more challenging to interpret a new drug’s molecular mechanism. The orthosteric binding site is the pocket (either shallow or deeply buried) where a protein binds its natural substrate. It is therefore the most obvious binding pocket for small molecules designed to modulate proteins that are misregulated in pathological conditions. By preferentially occupying the orthosteric binding site, a small molecule can prevent this site being occupied by its natural substrate. In contrast, allosteric binding sites are alternative pockets that, once occupied by a drug, may affect the molecular mechanism at the orthosteric site via cross-talk communication. In mechanistic terms, when a drug occupies an allosteric site, it alters the protein’s conformation or plasticity, thus changing its ability to bind and release its natural substrate at the orthosteric site. Allosteric binding can be used to achieve better drug selectivity. Indeed, while orthosteric sites are broadly conserved across wide classes of proteins (e.g., the ATP binding site in kinases), allosteric sites can be more specific, allowing more selective control over a protein’s function and limiting the side effects. Needless to say, allosteric binding is challenging to investigate computationally. This is because of the great number and variety of sites to be probed and because their a priori identification is often difficult.^7^

At the microscopic level, the interactions driving these molecular processes are known. In principle, one could use the laws of physics to predict the time evolution of even the most complex biomolecular transformation. The eventual feasibility of this idea is supported by remarkable developments in computational biochemistry and biophysics, which have already provided a meaningful understanding of various biological processes.^8^ However, we are far from achieving a general applicability of these approaches to pharmacology. This is because, in pharmacology, the local molecule–molecule interaction is just one part of the problem. One must also consider systems biology aspects of the various effects and the eventual fate of a compound introduced into a living organism. Nevertheless, our knowledge of the molecular basis and mechanisms of life is already so advanced that we now design new drugs by applying this knowledge to drug–target interactions and effects at the microscopic level. More importantly, this knowledge is rapidly increasing along with our ability to analyze, organize, and simulate reality by computational means.^9^

It will be useful, at this stage, to summarize the conceptual basis of these developments and to imagine what the future has in store for pharmacology as a result. We define and limit the scope of this review to the computational prediction of the equilibrium and nonequilibrium evolution of a complex consisting of a drug and its target in solution or perhaps embedded into a biomembrane, while neglecting most other effects from the host organism as a whole. Such computational predictions are challenging because a comprehensive description must cover a range of time scales, from the femtosecond period of molecular vibrations to the slow diffusion rate of all species in solution, up to the millisecond and beyond to follow the binding and unbinding of drugs and targets.^10^ From a physics standpoint, it should be possible to comprehensively describe these phenomena using a combination of quantum and statistical mechanics. This description could be summarized as a set of thermodynamics and kinetics relations, which could ultimately account for the affinity/potency of a drug toward its target and its efficacy in vivo. Hence, an accurate estimation of the thermodynamics and kinetics of drug-target interactions can provide useful information for predicting the efficacy/toxicity of a new medicine in the human body. From a chemistry standpoint, understanding the chemical mechanism responsible for the free energy and kinetics of the binding can help us to develop drugs with an improved therapeutic profile and reduced toxicity. Indeed, understanding how the atoms of a drug and its target interact is key to identifying chemical modifications to improve the drug’s thermodynamic and kinetic profile. There are several experimental methods for measuring the thermodynamics and kinetics of drug-target binding at the molecular level. These approaches can be biochemical (e.g., ELISA, enzymatic, and radioactive assays) or biophysical (e.g., surface plasmon resonance, isothermal titration calorimetry, and FRET).^11,12^ Supporting structural information is routinely provided by high-resolution X-ray diffraction^13^ and by neutron scattering.^14^ All these methods provide the (often extremely accurate) experimental values of thermodynamic (e.g., *K*_a_, *K*_d_, IC_50_, and EC_50_) and kinetic (e.g., *k*_off_ and *k*_on_) constants, which are necessary to progress a drug candidate through the discovery and development pipeline. These experimental observables are quantitatively related to the free energy and the binding/unbinding kinetics (*k*_off_ and *k*_on_) of the drug-target interaction. For instance, the Gibbs binding free energy is directly related to the equilibrium concentration of bound ([PL]) and unbound ligand ([L]) and protein ([P]) complexes, according to
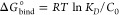
1where *T* is the temperature, *R* is the gas constant, and *C*_0_ is the standard state concentration of 1 mol/L. *K*_D_ is the dissociation constant (usually obtained experimentally at pH 7) and is defined by
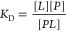
2

*K*_*D*_, in turn, is expressed in terms of the kinetic coefficients *k*_on_ and *k*_off_ through the relation:
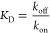
3In terms of equilibrium thermodynamics, the ergodic theorem then provides a suitable theoretical framework for linking the chemical world to the physical observables used to assess drug potency and efficacy. In particular, for closed systems, the time average of their properties is equal to the average over the entire space. This provides the statistical properties of a system in thermodynamic equilibrium. Molecular simulation can thus merge the microscopic and macroscopic worlds by estimating the time that the system spends in a certain microscopic state. If the simulations are sufficiently extensive, they can also estimate the probability of that state. This is becoming ever more feasible thanks to modern algorithms and efficient hardware architectures. The resulting in silico studies are extensive, covering a growing range of size and time scales. In drug discovery, recent microsecond-to-millisecond-long simulations have allowed the unbiased study of multiple processes of a ligand binding to a biological target.^15−19^

This emphasis on equilibrium thermodynamics should not distract from the fact that life is inherently a nonequilibrium process. Every living organism is an out-of-equilibrium system, powered by external energy and crisscrossed by fluxes of heat, chemical species, and ionic currents driven by a corresponding variety of gradients.^20^ Researchers are therefore starting to consider several scenarios of nonequilibrium. For instance, (un)binding kinetics, which is an out-of-equilibrium parameter, is attracting increasing attention. Recent publications have pointed out that, at least for some systems, the efficacy of a new drug in vivo (i.e., in nonequilibrium conditions) is highly correlated to the unbinding kinetics (or its reciprocal, known as “residence time”). Binding free energy is the classical quantity that correlates with efficacy, but kinetics is also relevant. There are many experimental and computational methodologies for measuring and computing residence time, which is a direct indicator of the time a drug spends in contact with its biological target.^21−26^ These approaches are being applied more and more by the drug discovery community. Nevertheless, one should always consider that efficacy in vivo is affected by many other factors, including metabolism and pharmacokinetics.

In this review, we report on recent progress in developing (and applying) molecular simulation approaches to calculate and predict the free energy and kinetics of drug-target binding. One section covers the theoretical background, outlining molecular dynamics and enhanced sampling. These methods are at the forefront of computational approaches to drug discovery. This is because they are increasingly capable of providing mechanistic and energetic (thermodynamics and kinetics) information at an unprecedented level of detail. Thanks to the availability of larger computational infrastructures and codes optimized for this hardware, it is now feasible to use previously prohibitive methods (i.e., MD and related methods) for computational drug discovery. The central section focuses on applications to drug discovery. In particular, we discuss the use of molecular simulations to estimate the free energy and kinetics of binding. First, we report on selected applications of molecular simulation to estimate the binding free energy. Some approaches estimate the absolute binding free energy. They require massive computations for adequate statistics and a robust estimation of thermodynamic observables. Other approaches estimate free energy differences within a series of congeneric molecules. These methods are mainly based on free energy perturbation and thermodynamic integration. They do not provide the absolute binding free energy. However, they are efficient in predicting potency difference, particularly within series of congeneric compounds. Alchemical methods and similar comparative approaches are nowadays widely used in the lead optimization phase of drug discovery. Then, we discuss the kinetics of binding and unbinding, which are emerging concepts in drug discovery and development. In terms of sampling, the binding and unbinding observables (*k*_on_ and *k*_off_) are related to the activation free energy. This can only be estimated with an accurate and exhaustive sampling of high-in-free-energy states in order to properly describe the probability density function of these points in the free energy surface. Here too, methods that compare the unbinding kinetics within a series of congeneric compounds are more practical for drug discovery, and their use is increasing. Accurate absolute (un)binding kinetics predictions are still very limited and are one of the biggest challenges in computational drug discovery. Next, we briefly report recent machine learning and deep learning trends, highlighting their scope and limitations for drug discovery and development. We then discuss some practical guidelines for the practitioner. Lastly, we discuss major challenges and perspectives.

This review offers the concepts and information necessary to properly understand the role and challenges of the various simulation approaches in drug design and discovery. It is therefore suitable for readers (including nonexperts) wishing to learn how molecular simulation can be used to obtain an in-depth molecular and mechanistic understanding of drug-target binding in terms of thermodynamics and kinetics.

## 2 Basics on Molecular Simulation

### 2.1 Models

The simulation of drug-target binding is a specialized branch of computational biochemistry and biophysics. As such, it largely uses the models and methods of this field. Let us consider a system made of molecules. We will focus on microscopic models, in which the molecules are represented by interacting particles that, in most cases, correspond to atoms. The system is thus described by a set of coordinates {**r**_*i*_; *i* = 1, ..., *N*} ≡ {**r**^*N*^} and their conjugate momenta {**p**_*i*_; *i* = 1, ..., *N*} ≡ {**p**^*N*^}, which collectively define the system phase space. Equilibrium properties, in particular, are expressed as averages of suitable distribution functions over the system phase space. In classical mechanics, one often deals with the configuration space, which comprises all the admissible coordinates.

Under the conditions of interest, classical mechanics provides a fair description of the system properties, covering the structure, dynamics, and overall time evolution. Equilibrium and nonequilibrium properties can be determined from knowledge of the system’s potential energy for every point in configuration space, which we define for simplicity’s sake as a single-valued function of coordinates *U* ≡ *U*({**r**^*N*^}). In the case of atomistic models, the potential energy can be determined ab initio using quantum chemistry methods or density functional theory. Biochemical and biophysical simulations, however, are the realm of molecular force fields, which split the potential energy into nonbonded and bonded interactions:
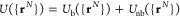
4

Bonded interactions depend on the molecular topology. This is defined by the distribution of covalent bonds among atoms. In popular force fields, bonded contributions consist of interactions up to four-body. A standard form for these terms is
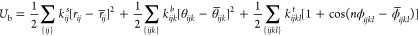
5where *i*, *j*, *k*, and *l* are atoms joined by consecutive covalent bonds, *k*_*ij*_^*s*^, *k*_*ijk*_^*b*^, and *k*_*ijkl*_^*t*^ are force constants, and *r̅*_*ij*_, θ̅_*ijk*_, and ϕ̅_*ijkl*_ are reference values for bond lengths, bending and dihedral angles, respectively, defining stretching (s), bending (b), and torsion (t) energy contributions. These are selected to reproduce molecular properties measured by spectroscopy or computed by ab initio methods. The integer parameter *n* in the torsional term reflects the (usually two- or 3-fold) periodicity of torsion potential. The single four-body term in eq 5 is sometimes replaced by a short Fourier sum over *n*. One can also include terms such as the Urey–Bradley potential and, more often, improper torsions.

Nonbonded interactions are primarily pair-additive and account for Coulomb forces, short-range repulsion arising from Pauli exclusion, and dispersion forces. By representing the last two contributions with, for example, a Lennard-Jones (LJ) potential, nonbonded interactions can be written as
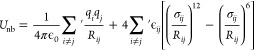
6where the {*q*_*i*_} are atomic charges, and σ_*ij*_ and ϵ_*ij*_ are the length and energy scales of the LJ potential. The prime on each sum indicates that pairs of atoms separated by one and two consecutive bonds are excluded, and the contribution from pairs separated by three consecutive bonds might be reduced, often by a factor of 2. Coulomb interactions act through the vacuum (of electric permittivity ϵ_0_) and, in most cases, are described within the rigid ion approximation, although there is increasing appreciation of the role of polarization contributions.^27^

Covalent bonds in organic chemistry are remarkably transferable from one molecule to another, opening the way for general force field parametrizations that are valid for large classes of compounds. Their broad coverage of organic molecules greatly eases the task of moving across the vast expanse of chemical space. Popular parametrizations include OPLS,^28^ Amber,^29,30^ Gromos,^31^ and CHARMM.^32,33^ Over the years, these parametrizations have led to more refined versions or have been specialized into different subsets that target more restricted classes of molecules. Thus, the current Amber parametrization for proteins is ff14SB, while GAFF,^34^ suitable for pharmacological applications, was developed to model ligands interacting with proteins. A similar evolution of CHARMM gave rise to several improved versions, exemplified by the popular CHARMM22, CHARMM27, and CHARMM36 parametrizations. Gromos generated the parameter sets 45A4, 53A5/6, 54A7, and 54A8, which are optimized for specific applications, such as computing the thermodynamic properties of liquids or targeting lipids and nucleic acid systems. The newest OPLS generation, as of 2019, is OPLS3.0e,^35^ which is also optimized for free energy computations. The large number of atom types in OPLS has prompted the development of a web server to carry out automatic parametrization of OPLS potentials.^36^ Further software tools^37^ (e.g., Antechamber in the Amber package) can facilitate the sometimes difficult task of analyzing the topology of complex molecules, writing the input for the corresponding simulation engine.^38^

Most parametrizations for ligands leave out atomic charges, which must be computed with semiempirical (AM1-BCC^39^ in the case of Antechamber) or ab initio methods on a case by case basis. However, the partition of the total electron charge among atoms is not unique. Popular methods for assigning charges^40^ to atoms (ions) include Mulliken, Löwdin, Bader, Davidson, and Hirshfeld.^41^ Fitting the electric field around a gas-phase molecule is an appealing approach, which underlies the so-called electrostatic potential model (ESP).^42^ It turns out that determining these fitting charges is ill-conditioned for all but the simplest molecules, and restraints are added in the RESP method.^43^ By construction, the fit considers only points of negligible electron density. Therefore, condensed phases cannot be used as the basis for the charge assignment. However, the ill-conditioning of the fit reflects the fact that many different sets of charges give nearly the same electrostatic field. Thus, the precise choice of charges might not be so crucial. There is an obvious physical reason for assigning charges that sum to an integer value (in units such that *e* = 1) for each molecular species in the system. However, this often results in low diffusion constants. These can be corrected by scaling charges by a factor of ∼0.8.^44^ This rescaling is generally seen as a very empirical way to account for polarization effects.

The lengths that define the molecular frame of covalent bonds may be kept fixed, or they may change in time according to the balance of intramolecular and intermolecular interactions. Fixed rigid bonds are enforced by constraints using methods such as SHAKE^45^ or LINCS.^46^ By removing the fast stretching modes, rigid bond models allow more efficient sampling of the remaining degrees of freedom.

Water force fields are a research subject in themselves.^47^ This is because of water’s importance, the complexity of its hydrogen bond network, and the several anomalies apparent in its phase properties.^48^ The simplest models (e.g., SPC, SPC/E, and SPC/Fw)^49^ treat the water molecule as comprising three atoms and two covalent bonds. The multipolar distribution of electron charge is better represented in four-site models, such as the various TIP4P^50^ models available in the literature. Finally, the tetrahedral symmetry of the sp^3^ hybridization of oxygen in water is better represented by five-site models, such as TIP5P,^51^ supplementing the three atomic positions with dummy particles to mimic the effect of the two lone pairs around oxygen.

Water models have had only fair success in reproducing the full complexity of the water phase diagram. Nevertheless, several of these models can complement the force field description of most biosystems in solution, sufficiently describing their structural, thermodynamic, and dynamical properties in the explicit solvent. As a consequence, no clear winner has emerged among the available water models in biophysics and biochemistry, and several water models are currently being used. However, it is advisible to ensure the consistency of the force field for water and the other molecular species in the system. For instance, the Amber ff15ipq force field has been parametrized for SPC/Eb water, and it should be used precisely in that combination.

Full-blown ab initio simulations of biosystems are not yet the norm, mainly because they cover only a short time frame. However, hybrid approaches such as QM-MM^52,53^ play an important role in modeling organometallic complexes (e.g., prosthetic groups in proteins) and in investigating reactions involving a localized change in molecular topology.^54,55^

### 2.2 Molecular Dynamics

Computer simulation determines equilibrium properties at nonzero temperatures. The first broad distinction is between molecular dynamics (MD) and Monte Carlo (MC) methods. The former computes trajectories in real time, the latter samples the equilibrium distribution over the configuration space. We will briefly detail the former here.

MD ability to compute equilibrium properties relies on the ergodic theorem, which states that the average over phase space of a sufficiently smooth operator  is the time average  over a microcanonical trajectory Φ(*t*) ≡{**r**^*N*^(*t*)}:
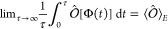
7

Trajectories, in turn, are determined by the numerical (and thus approximate) integration of the system equations of motion. For simplicity, we adopt the Hamiltonian formulation with Cartesian coordinates and focus on Newton’s equation of motion:
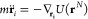
8where, again, {*i* = 1, ..., *N*}, *N* being the number of atoms in the system. Newton’s equations of motion are time-reversible, hence, in the absence of a time-dependent external field, the total energy is conserved.

The numerical integration is usually carried out by some form of discretization, evolving the system in small timesteps d*t* starting from a suitable initial state {**r**_*i*_; **p**_*i*_}. Many integration rules^56,57^ have been proposed and tested over the years, including a variety of predictor-corrector forms. At present, the Verlet algorithm and the virtually equivalent velocity Verlet are widely used.

In principle, the time step can reach, at most, one-hundredth of the highest vibrational frequency in the system. In practice, timesteps of 1 fs are the norm. This can be extended to 2 fs by increasing the mass of the hydrogen atoms or, more often, by fixing the length of every covalent bond involving an H atom. The integration of Newton’s equations of motion (eq 8) samples the microcanonical ensemble. Extensions to other ensembles are available, following precise prescriptions.^57^ One example is thermostats to maintain a specific temperature (i.e., NVT and NPT ensembles).

## 3 Enhanced Sampling Methods

Molecular dynamics is extensively used to sample the Boltzmann equilibrium probability distribution in phase space and to reproduce the real-time dynamics of macromolecules and biosystems. Despite the validity (up to the force field representation capability) of these methods, systems and phenomena of interest for drug discovery still pose a severe challenge, partly because of the complexity and size of the systems of interest, and especially because of the wide range of time scales spanned by phenomena such as the ligand-protein binding and unbinding or the protein folding. These phenomena typically require milliseconds but reach up to seconds and beyond. Considering, for instance, a time step of 1 fs in MD, it is important to verify that sampling events in the millisecond or second time scale require 10^12^ or 10^15^ integration steps. This amount of computing time is far beyond the current available computational technology, making such endeavors unfeasible. Practical considerations of this type have until now hampered the use of simulation in fields such as drug discovery, making methods such as docking^58^ the de facto standard. The recent advent of graphical processing units (GPUs) has partially mitigated this issue, allowing the microsecond time scale to be easily achieved. However, the millisecond and seconds time scale are unavailable to most researchers, apart from very specific efforts.^59^ One significant technological effort is the D.E. Shaw group’s development of a dedicated hardware for MD only, called Anton.^59^ This unique and expensive hardware solution has achieved millisecond time scales, demonstrating MD’s reliability in reproducing the protein–ligand binding process.^17^ However, the seconds time scale is still elusive. Despite the significant technological achievements of the last 20 years, certain phenomena simply cannot be simulated via plain MD. This state of affairs is likely to continue for years to come. Besides these practical considerations, the pedestrian extrapolation of methods devised for simpler systems and problems to a whole new domain is also conceptually unattractive.

Two related but distinct needs are apparent in the drug discovery context. First, one must sample a complex landscape in configuration space, consisting of hierarchically organized basins, separated by barriers, causing the near breaking of ergodicity. The second and more difficult challenge is to quantify the kinetics of biosystems evolving under stationary state or fully nonequilibrium conditions.

**Figure 1:**
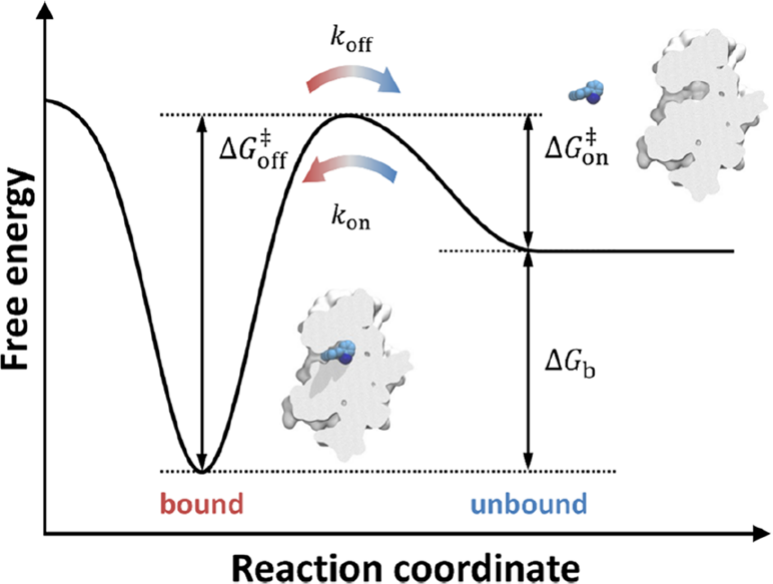
Idealized potential of mean force for protein–ligand binding. The reaction coordinate represents the observable that allows the binding process to be tracked. Reproduced from ref (60). Copyright 2016 American Chemical Society.

A shared feature of these sampling methods is a way of accelerating the events of interest. From a Bayesian standpoint, some constitute a class of methods where a priori information is used to focus the sampling in specific regions of the phase space. This acceleration can be obtained in several ways, such as adding an external potential to the original one, defining proper restraints to collect statistics in a specific point of phase space and morphing the system Hamiltonian with a reference one. These methods are not only able to accelerate the sampling but in some cases also allow a free energy reconstruction. To achieve enhanced sampling, collective variables are often used: these order parameters include distances, angles, RMSD, and, in general, more or less complex observables, whose changing values represent the index of evolution of the phenomenon under analysis. We will call such an observable ξ. In the first approximation, for instance, the distance between two groups can represent the obvious reaction coordinate for a protein–ligand binding study (see Figure 1).

In the following sections, we briefly present a series of enhanced sampling and free energy methodologies that are currently used for protein–ligand binding problems.

### 3.1 Steered MD

**Figure 2:**
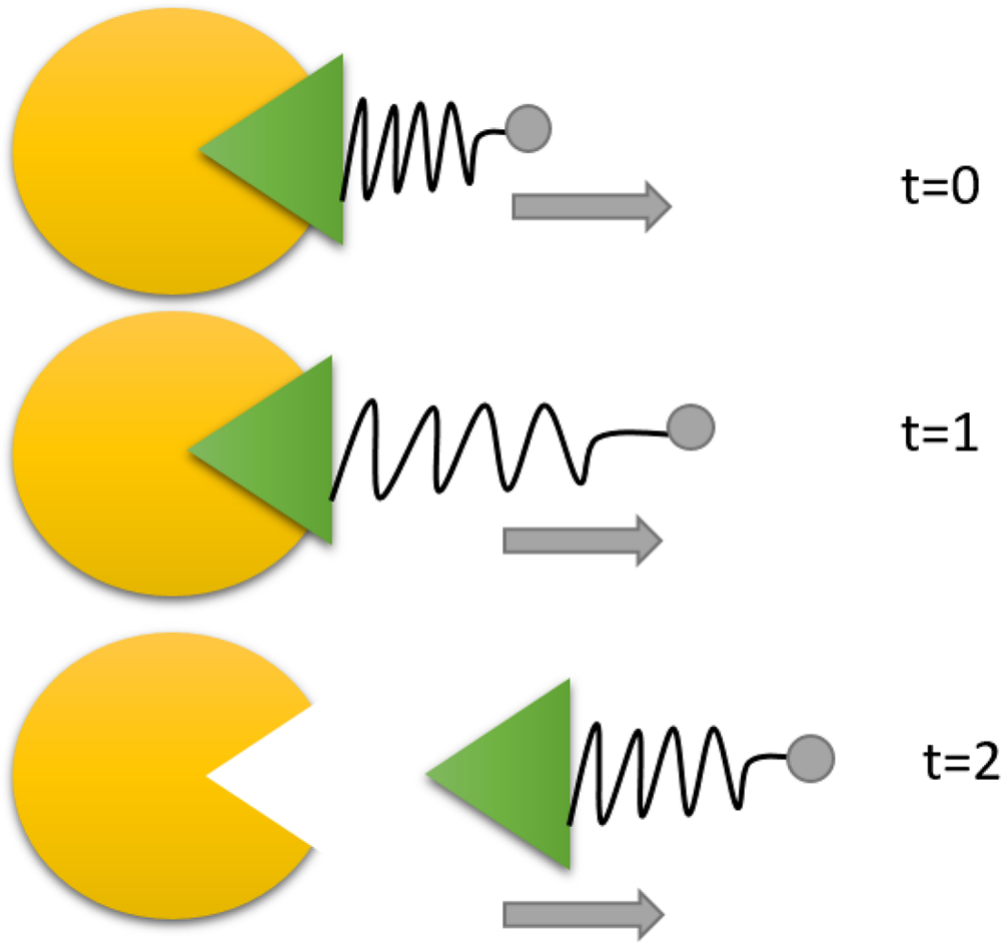
Graphical representation of steered MD in the protein–ligand case. The yellow component represents schematically a protein and the green component represents the ligand. A spring is attached to the ligand, and the center of the spring (in gray) is moved along time by increasing distances to promote the unbinding event. Time is increasing from the top in arbitrary units.

In the steered MD methodology, one adds to the plain MD potential *U* a parabolic potential Δ*U* to increase the probability of sampling a specific phase space region (see Figure 2). Additionally, the center of the parabola moves in time over the desired range of the reaction coordinate ξ. In detail, one has
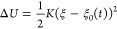
9where the center ξ_0_(*t*) often moves at constant velocity as in
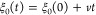
10*v* is the value of the constant velocity in the collective variable space.

In a fundamental work,^61^ Park and Schulten developed a theory for extracting the potential of mean force (the free energy profile) from these kind of simulations. Namely, they considered how a nonequilibrium process such as steered MD can be connected to an equilibrium concept such as the potential of mean force. In turn, the theory in ref (61) is based on an important relation in statistical mechanics, the Jarzynski’s equality, derived in ref (62). The free energy can be reconstructed by running several independent replicas of the same steering process.

### 3.2 Adiabatic Bias Molecular Dynamics

Adiabatic bias molecular dynamics (ABMD) is a conceptually simple method for navigating the phase space. It is particularly well-suited to reaching a given target value in collective variable space.^63^ The key aspect of this biasing method is that the applied perturbation conserves a characteristic energy. Suppose ξ is the reaction coordinate and the bias at time *t*_*n*_ is
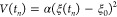
11

**Figure 3:**
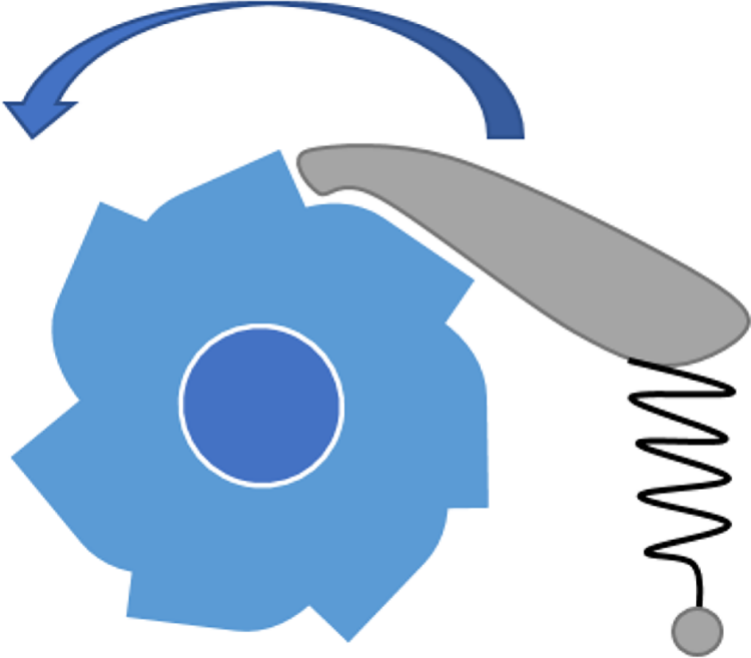
ABMD is similar to a pawl and ratchet system. The collective variable can evolve in one direction only (the wheel rotation); it is restrained if it tries to go in the opposite direction (the wheel is stopped if it tries to go back).

Then, the center ξ_0_ is updated dynamically based on the advancement or not of the collective variable in the desired direction. Suppose that an increasing ξ is desired then the update equations for the center become:
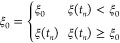
12Evidently, if a decreasing ξ is desired then the opposite update equations hold. ABMD can be seen as an analog of the pawl and ratchet mechanical system (see Figure 3). The wheel (the collective variable) can only progress in one direction. If the system tries to move in the wrong direction, a harmonic restraint prevents these motions. It is similar to steered MD, but the key difference here is that the speed at which the center is moved is not ruled by the user only but also by the natural evolution of the process toward the final value of the collective variable. Still, the user can tune the process speed by modifying the restraint constant; the higher this constant, the stronger is the reluctance of the simulation to visit previous stations and thus the higher the speed. ABMD can be interpreted as an adaptive and gentle version of steered MD.

### 3.3 Parallel Tempering

Parallel tempering is a technique (or, more properly, a family of techniques) which allows one to overcome free energy barriers without explicitly introducing a collective variable.^64^ In this enhanced sampling technique, the increased sampling capability is achieved through the increase of the temperature. Identical replicas of the same system, differing only in the temperature, are run in parallel. Let *M* be the number of parallel replicas run at different temperatures *T*_*i*_ where *T*_1_ is the correct base temperature and the other ones for *i* > 1 are higher. Then, these parallel simulations are allowed, from time to time, to exchange the configurations between consecutive (e.g., *i* and *i* + 1) replicas. This allows a constant exchange of configurations and thus the migration from one high temperature configuration to a low one, and thus the correct sampling, at low temperatures, of a free energy basin, which would not have been visited with the low-temperature simulation only. Important aspects include the number of replicas and the exchange probability in order to maximize sampling efficiency and ensure successful exchanges between replicas. Parallel tempering satisfies the detailed balance, since exchanges between replica *i* and replica *j* are accepted with probability:
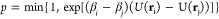
13Among other features, to achieve equal acceptance ratios, a geometric progression of temperatures is required. For concision, we will not discuss here the many other technicalities of an efficient tempering protocol.^64^

However, tempering techniques are not a method but a class of methods. In addition to replicas at different temperatures, the exchange could also involve other observables. For example, the bias in parallel metadynamic runs can be exchanged.^65^

### 3.4 Scaled MD

**Figure 4:**
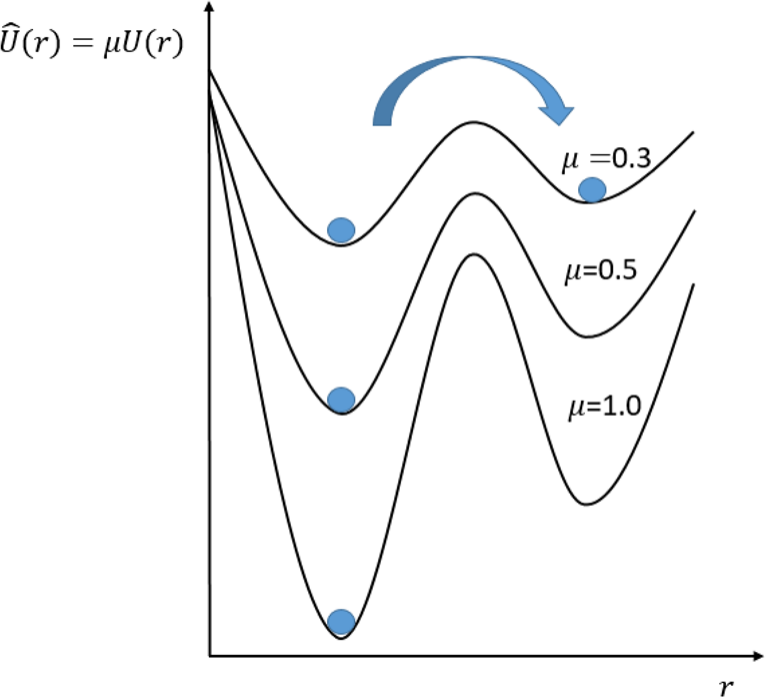
Scaling the potential by μ < 1 increases the crossing rate between the two basins. A constant scaling is equivalent to a 1/μ scaling of the temperature.

Scaled MD is an extremely simple enhanced sampling method. It has been used to increase, in a simple temperature-like way, the probability of escaping from free energy minima.^66−68^ If we define a positive constant μ ∈ (0, 1] then we define a new potential:
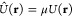
14where *U*(**r**) is the potential energy of the system. Hence, μ = 1 is plain MD, and intermediate values represent a more or less pronounced scaling of the potential (see Figure 4).

The scaling action can be interpreted as a smoothing factor of the potential, which uniformly flattens all the barriers in the potential energy surface. This last property was recently used^25,69−71^ to accelerate the unbinding and binding process. For the case of unbinding,^25^ in addition to the potential scaling, one applies harmonic restraints on the part of the protein backbone that is not involved in the binding. This prevents unfolding. Upon scaling, the time when the ligand is completely surrounded by water molecules for the first time is defined as unbinding time. The unbinding process is repeated several times (usually at least 20) to define an average unbinding time. Compounds are ranked according to this time. This methodology has been successful^25,69,70^ in providing a *k*_off_-based ranking of compounds. This protocol is widely applicable thanks to the reduced number of free parameters, the absence of a reaction coordinate, and the relatively fast computing time. One disadvantage is that, due to the presence of restraints, one requires a priori knowledge of the residues involved in the binding/unbinding process. Although this is formally different from a reaction coordinate, the role is similar. A second disadvantage is that a heavy scaling, while useful for ranking, can sometimes lead to significantly approximate unbinding trajectories. This, in turn, makes it difficult to obtain clear mechanistic insights into the unbinding process. In addition to accelerating the unbinding process, this methodology has been applied to the dynamic docking process.^71^ In this last case, together with the potential scaling, a cylinder-shaped wall is used to restrict the configurational space that the ligand explores, thus increasing the local concentration and hence binding probability.

### 3.5 τ-RAMD

The τ-RAMD protocol was recently proposed by Wade and co-workers^24^ for studying the residence time of some HSP90 binders. The protocol is built upon the random acceleration molecular dynamics simulation method (RAMD) [also known as random expulsion MD (REMD)]. The method involves periodically applying a random force on the ligand during a prescribed time window. If the ligand does not move in the desired direction (assessed by a distance threshold) then the force is reassigned randomly. This simple procedure is effective in accelerating the unbinding time by several orders of magnitude with respect to the physical unbinding time. On average, between 40 and 200 simulations were run for each compound and the mean unbinding time τ (from which the name derives) was used to build correlations. RAMD is a kind of supervised method, in that the randomness of the force is coupled with a prescribed albeit obvious collective variable, namely the distance that accounts for the unbinding progress.

### 3.6 Metadynamics

Metadynamics (MetaD)^72^ is a method for escaping local free energy minima. Metadynamics is part of the family of adaptive bias methods, where a history-dependent bias is modified over time to ideally achieve a fully diffusive behavior on the chosen reaction coordinate. Among other methods in this family,^73−79^ we discuss metadynamics here because of its widespread use and availability in the computational drug discovery community.^80,81^ In the first version of metadynamics, constrained and coarse-grained simulations were used.^72^ Later, a continuous version emerged.^82^ Here, we discuss this second version, which is widely used.

**Figure 5:**
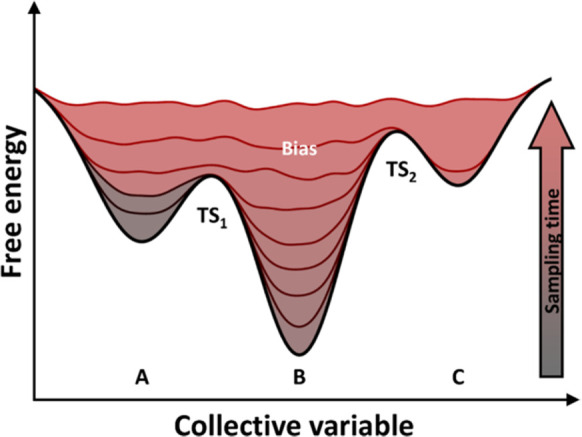
Metadynamics bias potential time evolution. Basin A is filled with Gaussians first, then basin B, and finally basin C until convergence. Reproduced from ref (60). Copyright 2016 American Chemical Society.

Given a vector of reaction coordinates **ξ** of dimension *d* at time *t*, the metadynamics bias potential is
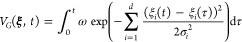
15where ω is an energy rate and σ_*i*_ is the width of the Gaussians corresponding to the *i*th CV. The term ω is the ratio between Gaussian height *W* and a Gaussian deposition stride τ_*G*_. The method eventually achieves a time-continuous deposition of Gaussians along the collective variable space (see Figure 5).

In contrast to umbrella sampling (discussed later), the key advantage of metadynamics is that it automatically explores the collective variable space and computes the free energy when at convergence. This can be seen as an advantage or a drawback because the sampling is not under the user’s control. This is in contrast to umbrella sampling, where the sampling is particularly controlled. Under some hypothesis,^82^ and based on empirical observations, one has
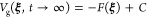
16where *F* is the free energy surface and *C* is an arbitrary additive constant. The error associated with the reconstruction is also proportional to^83^
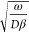
17where *D* is the diffusion coefficient in the ξ space, and β = (*k*_B_*T*)^−1^. However, it is often daunting to compute this error. Independent runs are therefore used to assess the reliability of the free energy surface. Despite this set of shared positive features, metadynamics presents two significant drawbacks. First, given that the Gaussian deposition is continuous in time, it is difficult to understand when to stop a metadynamics simulation. Additionally, stopping a simulation at a certain point means getting an arbitrary bias in the free energy surface due to the last deposited Gaussian, which can happen at any point of ξ if one assumes a nearly diffusive regime. Second, there is no perfectly satisfying proof of its convergence.

These compelling problems led to a solution called well-tempered metadynamics (WTMetaD),^84^ which was defined to fix the first problem and then proved to converge^85^ to the real free energy surface for sufficiently long simulation times.

### 3.7 Mechanisms and Kinetics of Rare Events

Many events of interest for biophysics and biochemistry correspond to what one could loosely call a rare event. Examples of rare events include the crossing of a reaction barrier, the rotation of a protein domain, the flipping of a phospholipid molecule in a biomembrane, and the absorption or release of a ligand by a receptor. The defining property of these phenomena is that, at equilibrium, they are separated by a long waiting time τ_w_, while the event itself takes place over a short time τ_ev_, which is a tiny fraction of the time τ_tot_ = τ_w_ + τ_ev_ required to investigate the phenomenon. No clear precursor allows one to identify or trigger the beginning of the transition state. Hence, the task is challenging due to the need to cover τ_tot_ at a resolution sufficient to analyze τ_ev_. Fortunately, in recent years, advances in hardware and algorithms have pushed back the challenging range of τ_tot_/τ_ev_ by orders of magnitude. However, the broad distribution of characteristic times in complex biosystems means that the exploration of rare events is still a great challenge. Here, we briefly present some methodologies that specifically address the rare events problem and that have been applied successfully to protein–ligand binding problems.

#### 3.7.1 Markov State Models (MSM)

Markov State Models are a statistical method that can be applied to a set of plain MD simulations to retrieve kinetic information. The first task in building an MSM is to subdivide the configuration space into a complete partition of nonoverlapping sets {*A*_1_, ..., *A*_*n*_}. Then, the basic quantity defining the model is the matrix of transition rates among these macrostates. We name this quantity *T*. More precisely, *T* is an *n* × *n* matrix, whose *T*_*ij*_ matrix gives the probability of state *i* going to *j* within a time scale τ, known as the lag time. This must be long enough for transitions to be memoryless^86^ (Markov property assumption) and short enough to allow for high resolution. The Chapman-Kolmogorov test can be used to validate the choice of lag time compatible with the loss of memory assumption.^87^ The diagonal elements *T*_*ii*_ represent the probability for the system at state *i* to remain in the same state. Because of general properties of probability, the elements on each row of *T* sum to 1. All transition probabilities given by *T* represent equilibrium properties, measuring the diffusion of the system over discrete states due to thermal fluctuations. Once built, the model can be interrogated to predict the long-term evolution of a system prepared in an out-of-equilibrium state, relying once again on the relaxation-fluctuation theorem. The results of the first development stages can drive the model’s refinement, requiring the redefinition of the set of states {*A*_*i*_} and the computation of new transition rates using targeted and relatively short MD runs. In this basic form, MSM has been used to analyze protein folding^88−90^ and protein–ligand binding simulations.^15,16,91,92^ MSMs are particularly efficiently applied to diffusive problems, whereas their application to activated (high barrier) problems is less efficient. This is because, in the original formulation of MSMs, the user simply runs many independent plain MD simulations without any prescribed strategy to decide when and where (in phase space) to start a new simulation. This is in contrast to transition path sampling methodologies.^93,94^

Needless to say, this quick overview omits many important details. For more detail, the interested reader is directed to the original papers, or their convenient summaries in recent reviews (see refs (89, 95−97)).

#### 3.7.2 Weighted Ensemble

An original variant of the more general path sampling^93,98−104^ is represented by the weighted ensemble (WE) method,^105^ which was originally conceived by von Neumann and then revived and first implemented with the Huber-Kim algorithm.^106^

As in many path sampling methods, the separation between the initial state A and the final state B is divided into partitions (bins, in the WE language). One starts a set of trajectories (say *M*) in the bin containing A, and the algorithm alternates simulations advance for a (relatively short) fixed amount of time τ to resampling steps where trajectories are pruned or spawned, keeping the number of walkers within each bin invariant. The cycle is repeated until state B is reached. The time evolution step may follow whatever dynamics (microcanonical MD, stochastic, Brownian, etc.) is deemed suitable for the problem at hand.

The potential energy surface underlying trajectories is unbiased. In simple cases, in which the A → B involves two basins only and a single barrier between them, kinetic rates can be computed directly from the trajectories joining the two basins. Whenever intermediate states are present, this procedure becomes inefficient and a postprocessing stage may be needed to compute rates, in which the unbiased trajectories are used to estimate the hopping rates among bins, opening the way to reconstruct the steady state. In this approach, kinetic rates are usually expressed as first passage time (FPT).

This brief outline already points to the close relation of WE to Markov state models. Compared to MSM, the correlation of trajectories due to their resampling makes WE somewhat more efficient and, on contrast to MSM, WE does not rely on a Markovian assumption for the transitions among bins. Moreover, it turns out that MSM prediction depends more heavily on the definition of free energy basins than the WE estimates of kinetic parameters depend on the choice of the bins.^107^

Besides similarities with both transition path sampling and MSM, WE possesses several interesting (nonexclusive) properties. The most remarkable property is that WE provides unbiased predictions on time scales longer than the aggregated duration of the underlying dynamical simulations. This property derives from the validity of the Hill relation, expressing the mean first passage time (MFPT) as a function of steady-state fluxes (FL_LL_):^108^
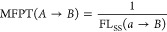
18Moreover, WE can describe both nonequilibrium steady state conditions and equilibrium, which is a special case of steady state. Its multitrajectory character makes it suitable for describing transitions that occur following different pathways. The same multitrajectory aspect makes WE easy to parallelize. The definition of bins does not need to remain unaltered from the beginning to the end of simulations, but it can be defined by an adaptive strategy, as implemented in WExplore.^94^ Last but not least, although not widely exploited yet, WE is rather scale-neutral and can be used to describe a wider variety of dynamical processes than simply the time evolution of particles.^105^

It has been claimed that WE does not need any collective variable, although a careful analysis of the algorithm shows that the definition of bins relies on a metric, such as the displacement of a molecule from its initial position. A metric is nothing other than a collective variable. This claim is shared with MSM, where one can also argue that the MSM definition of basins depends on a metric, hence on a collective variable.

WExplore, first introduced in ref (94), is a recent and effective version of the Weighted Ensemble protocol for biomolecule and biophysics simulations. In this strategy, bins are dynamically and hierarchically defined, thus avoiding the problem of defining bins a priori, while also reducing a high-dimensional order parameter space to a manageable size. Bins consist of Voronoi polyhedra on the space of the sampling variables. Then, to test whether a trajectory belongs to a specific region, computations on the configuration can be used inside the hierarchical tree.

## 4 Methods for Free Energy Computations

In this section, we provide a concise description of some of the most currently used methods to compute free energy in protein–ligand binding problems.

### 4.1 Umbrella Sampling

**Figure 6:**
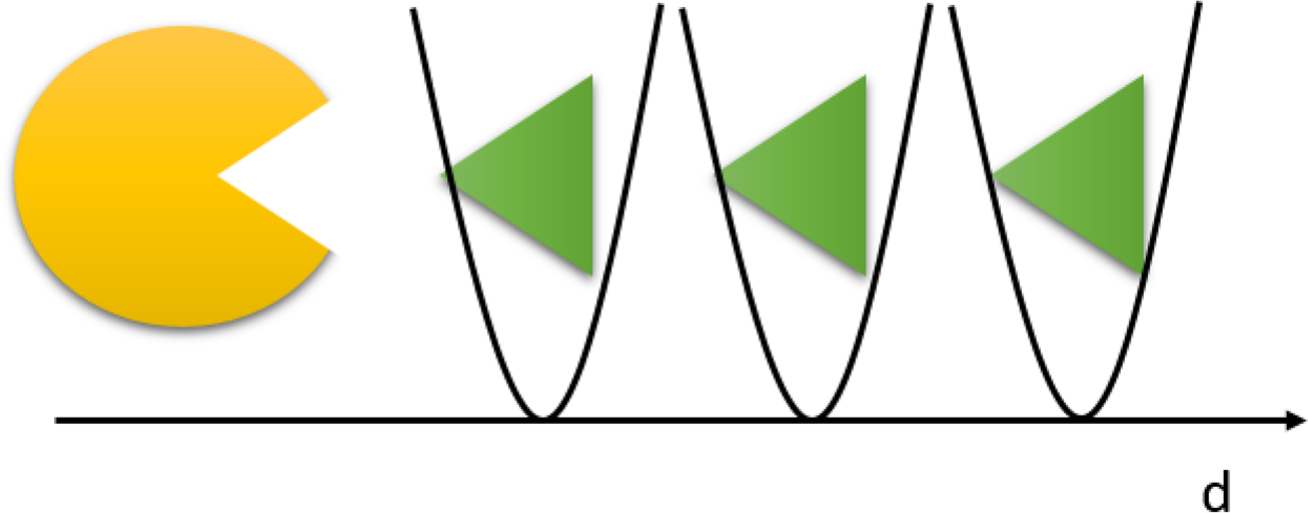
Simplest umbrella sampling scheme for protein–ligand binding. Harmonic restraints are applied along the distance connecting the protein (in yellow) with the ligand center of mass (in green).

To provide unbiased results, approaches for free energy computations need to visit all the relevant configuration space, overcoming barriers that may divide it into barely connected basins. Umbrella sampling^109^ is historically the first and one of the most popular methods used to enhance the sampling in the presence of near-nonergodicity conditions. Umbrella sampling is the progenitor of the family of enhanced sampling methods. The method derives its name from its ability to cover different basins of the configuration space. In this technique, similarly to steered MD, instead of sampling with the potential *U*(**r**), one is sampling with the potential
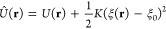
19where the second term is a harmonic restraint centered on ξ_0_ (see Figure 6 for a simple application to protein–ligand binding).

Reconstructing the free energy profile over a broad interval of coordinates is possible but not trivial. First, one simulation might not be sufficient to cover the range of the ξ variable of interest. Second, once one realizes that several centers are required to cover the space, one needs a method to recombine the umbrella sampling information into a unique free energy profile. Indeed, assuming that one is analytically able to reconstruct the free energy on each center, then, considering that the free energy is always known up to a constant, one should find a way to align the various free energies from each simulation into a unique profile. This is the aim of the Weighted Histogram Analysis Method (WHAM).^110^ However, WHAM is not the only method for reconstructing the free energy [the Multistate Bennett Acceptance Ratio (MBAR)^111^ is a notable example]. There is a second class of methods that directly leverage the mean force concept without the need to align the free energies from the different simulations.^112,113^ In several ways, these methods are an adaptation of thermodynamic integration to umbrella sampling, where a generalized force is considered to reconstruct the free energy profile.

#### 4.1.1 Computing the Standard Binding Energy

Umbrella sampling simulations can recover the potential of mean force profile. However, attention is required to move this quantity to a free energy of binding that is comparable to results from experiments. Indeed, to rigorously compare computational values to experimental quantities, one should resort to the standard free energy of binding.^114^

To do so, one must first observe that
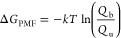
20where Δ*G*_PMF_ is the free energy difference from the PMF and *Q*_b_ and *Q*_u_ are the partition functions of the bound and unbound regions, respectively. Let *W*(ξ) be the PMF profile, that is the reversible work profile of the binding process, then one has^114^
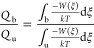
21where the two integrals are on the bound and unbound partition of the PMF profile. To get the standard free energy of binding Δ*G*^*o*^, we must take into account the free energy contribution for moving from the standard-state volume *V*_0_ = 1661 Å^3^, which corresponds to a *C*_0_ = 1 M concentration, to the actual unbound volume sampled during the simulation. Finally one gets
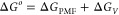
22this quantity is a formally correct quantity to be compared to free energies coming from experimental values. In detail, one has
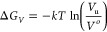
23where *V*_u_ is the unbound volume sampled along the simulation. In umbrella sampling simulations, there are often restraints applied orthogonal to the reaction coordinate. In this case, an additional free energy term must be taken into account.^114^ Throughout the text, for simplicity, we will use Δ*G* to indicate the binding free energy.

### 4.2 Adaptive Biasing Force

The adaptive biasing force method was first theoretically founded in ref (115) then rediscussed and popularized in ref (116). Similarly to thermodynamic integration and to some of the reconstruction techniques for umbrella sampling, this method is based on the concept of mean force.^117^ Here, one estimates on the fly this mean force acting on the reaction coordinate. At the same time, a bias opposing the mean force is applied, such that one can escape local free energy minima. Then, on a long time scale, as the running average of the mean force converges to the true mean force, the total force felt by the system virtually vanishes. This, ideally at convergence, allows for a diffusive regime over the entire range of the collective variable and the free energy to be estimated.

### 4.3 Relative Binding Free Energy

The free energy of binding of a molecule to a receptor Δ*G*_bind_ (or more precisely the *K*_D_) can be reliably experimentally measured.^118^ Nevertheless, a computational machinery able to predict the experimental values would be useful, saving time and reducing the cost of a fully experiment-based drug discovery campaign. Computing Δ*G*_bind_ can be done, for example, using the double annihilation^119^ or the double decoupling method^120^ (see details later). The latter differs in the details of the system transformation and especially in their rigorous use of position restraints. In these methods, a thermodynamic cycle is used to efficiently compute Δ*G*_bind_. This class of methods, in which molecular entities appear and disappear in the simulation box, are commonly referred to as alchemical methods because they follow a nonphysical path to perform the transformation. Since free energy is a state function, the nonphysical nature of the path followed is irrelevant from the theoretical viewpoint.

**Figure 7:**
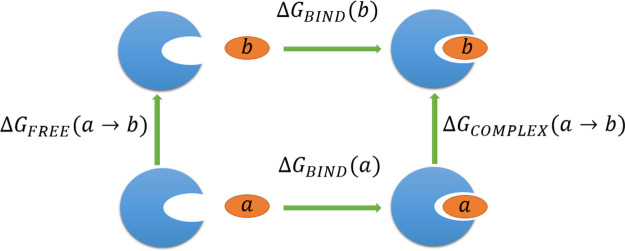
Thermodynamic cycle used to compute relative binding free energies (Δ*G*). Horizontal transformations are difficult as they require the complete annhilation of the ligand in the site and the appearance of the ligand in the solvent. The vertical transformations are more convenient as they are simple perturbations and they can be used to estimate the relative binding free energy.

Now, we detail the slightly simpler yet extremely useful case, in which one seeks a relative binding energy between drugs. Here, instead of annihilating an entire entity (such as a ligand), we only morph the changing part of the ligand (or the protein). Figure 7 depicts a thermodynamic cycle, in which one ligand *a* is mutated into ligand *b*.

The importance of this thermodynamic cycle arises from the fact that, while horizontal transformations in Figure 7 are hard, the vertical ones are significantly simpler. Indeed it holds that
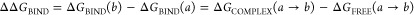
24This forms the basis for the application of the free energy perturbaton (FEP) method to compute differences in the binding free energy in series of ligands. The methodology has had some success^121,122^ and is a strong candidate protocol for prioritizing ligands during lead optimization.^123^

To perform the ligand mutations in the binding site and in the bulk, one must build topologies for both end states. Then, two different ways to morph ligand *a* into ligand *b* can be chosen, known as the single topology and the dual topology methods, respectively. In single topology, one specifies a set of force field parameters at each stage of the transformation. These are often taken as the weighted average of the end-point parameters. In this way, an atom (e.g., an O) can mutate into a different atom (S) literally in place. In dual topology, the potential energy system is a given interpolation of the two end-point energies, such as
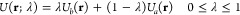
25where *U*_*b*_ is the potential of the destination state and *U*_*a*_ is the initial state. In this way, only the end-point force field parameters need to be specified and, during the transformation, the original O atom and S atom coexist, albeit in scaled forms. There is no clear consensus on which is the best approach for performing the transformation. On the one hand, the single topology approach minimizes the number of transformations, thus facilitating convergence. On the other hand, one is elongating and shortening chemical bonds, which is never a weak perturbation. A further problem with dual topology is that, close to the end states, emerging atoms can clash against the residual component of vanishing atoms. The problem is mainly due to the singularity of the van der Waals potential. For this reason, a modified soft core potential was introduced:^124^
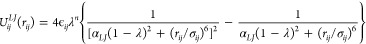
26where α_*LJ*_ is a positive constant and σ_*ij*_ and ϵ_*ij*_ are the Lennard-Jones parameters. Consistently, the Coulombic contribution is also changed to
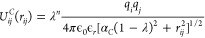
27where ϵ_0_ϵ_*r*_ is the dielectric constant of the medium and α_*C*_ is a positive constant (see^125^ for further recent developments).

The baseline FEP protocol has been extended in several ways, with the FEP/REST^122^ approach being one of the most notable. REST stands for replica exchange with solute tempering. In contrast to classical replica exchange, the advantage of this variation is that the hot region is restricted to the solute, thus excluding water molecules. This, in turn, significantly reduces the number of replicas needed with respect to parallel tempering. For FEP, the hot region comprises the ligand and the nearby residues. The protocol associates a specific solute temperature with each of the m λ windows. In particular, the series is (λ_0_ = 0, *T* = *T*_0_), (λ_1_, *T* = *T*_1_), ···, (λ_*m*/2_, *T* = *T*_*h*_), ···, (λ_*m*–1_, *T* = *T*_1_), and (λ_*mS*_, *T* = *T*_0_), where *T*_0_ is the physical temperature and *T*_*h*_ is the maximal physical temperature. The increased solute tempering ensemble allows the conformational space to be sampled more efficiently, overcoming potentially high energetic barriers^122^ (e.g., in dihedral space).

### 4.4 Double Annihilation and Double Decoupling Methods for Absolute Binding Energy

Beside relative free energy estimators, absolute binding energy is also important, although not yet so widely used in drug discovery. Two important methods in this class are the double annihilation and the double decoupling method, with the latter being a rigorous version of the former.

Denoted by the ligand (L), the protein (P), and the protein–ligand complex (PL), and using the subscripts wat and gas to denote the water and gas phases, one wants to compute Δ*G*_bind_:
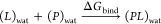
28

In the double annihilation method,^119^ alchemical transformations are used to compute the absolute binding energy Δ*G*_bind_. In particular, the computation of the free energy is split into two components Δ*G*_1_ and Δ*G*_2_:
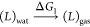
29
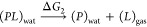
30

In the first of the two equations, the ligand is transferred from the water to the gas phase. In the second phase, the ligand is still transferred to the gas phase when in a complex with the protein. From these two phases, the method takes the name double annihilation, and the free energy is computed with
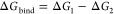
31This procedure has some problems. First, the rigorous free energy depends on the standard state,^120^ but this estimation does not take it into account. Strictly speaking, results from this procedure cannot therefore be compared to experimental results. The second problem concerns the sampling. During the decoupling phase in the second step, the ligand is completely decoupled from the protein. This means that the ligand is free to sample the entire simulation box. To get converged results, the ligand should explore all the possible orientations and positions in the simulation box, which makes the endeavor very difficult. To overcome these limitations, Gilson proposed the double decoupling method.^120^ In this thermodynamically correct version of the double annihilation method, restraints are introduced to maintain the ligand in the binding site during the second step. This trick avoids the sampling problem and obtains a correct standard binding free energy. This additional restraint introduces the need to compute the free energy component due to the restraints themselves. In general, this can be difficult: for protein–ligand binding, Karplus and co-workers^126^ obtained a simple and elegant analytical estimation of this additional free energy component. This gives the double decoupling scheme a rigorous and elegant formulation and practical applicability for the protein–ligand binding problem.

### 4.5 MM-PBSA and MM-GBSA

The MM-PBSA (Molecular Mechanics, Poisson–Boltzmann, and Solvent Accessible) and MM-GBSA (Molecular Mechanics, Generalized Born, and Solvent Accessible) methods^127−131^ represent classes of popular methods, devised to compute absolute and relative free energies, whose accuracy and reliability lie between scoring functions^132^ (or machine-learning black box models) and more rigorous physics-based methods such as FEP.^133^ The rationale of these methods is to trade some accuracy for computational speed, achieved by resorting to an implicit solvent model and to an approximate and largely empirical approach to computing free energies.

**Figure 8:**
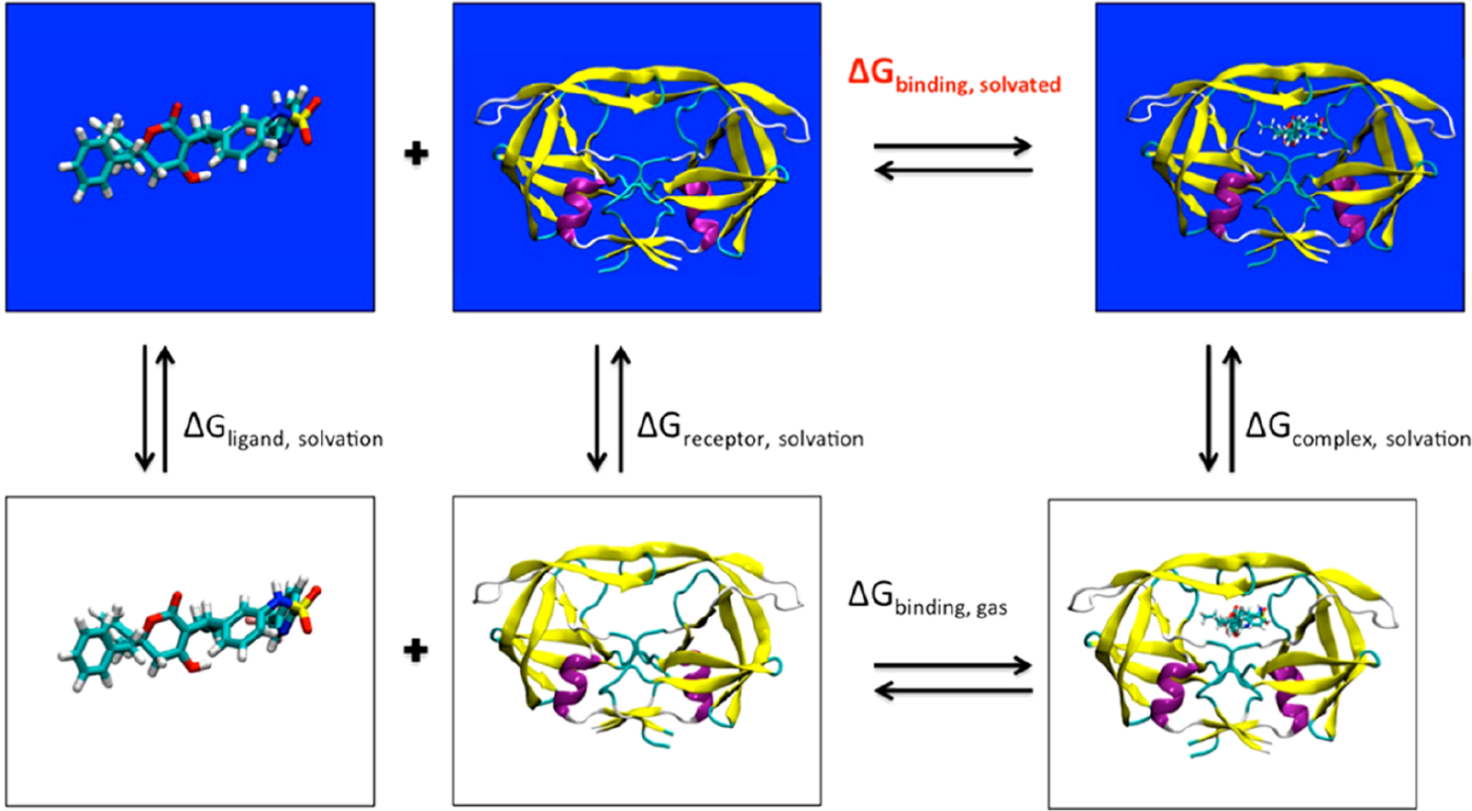
MM-PBSA binding free energy calculation with a thermodynamic cycle. In black, the Δ*G* terms that are explicitly computed. Reproduced from ref (130). Copyright 2012 American Chemical Society.

The MM-PBSA method was originally proposed by Kollmann and co-workers^127^ and is now widely used in the drug discovery community, including pharma companies. In this class of methods, the binding energy of a ligand to a protein is usually computed using the familiar relation:
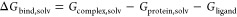
32where *G*_complex,solv_, *G*_protein,solv_ and *G*_ligand_ in principle are absolute free energies. The binding free energy can also be estimated with a more complex thermodynamic cycle (see Figure 8).

Both MM-PBSA and MM-GBSA are usually intended as postprocessing stages running on top of a standard MD or MC simulation based on a classical (sometimes ab initio) force field with explicit solvent. The free energy of each component (i.e., complex, protein, ligand) is computed by averaging:
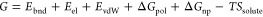
33over a set of configurations extracted from the simulation trajectory. In this last equation, the first three terms are classical molecular mechanics contributions and Δ*G*_pol_ + Δ*G*_np_ are estimates of the solvation free energy, divided into the polar and nonpolar component. The polar component is obtained from the Poisson–Boltzmann equation solution or from Generalized Born approaches, whereas the nonpolar component is often expressed as a linear function of the solvent-accessible surface. Often, the nonpolar contribution plays a lesser role with respect to the polar one.

The average over configurations implies the choice of the ensemble, from which the conformations are to be sampled. In this respect, a further simplification is often made by simultaneously selecting representative configurations for the complex, the protein, and the ligand from the single trajectory of the complex in solution, roughly halving the simulation time.

In addition to efficiency considerations, this single-trajectory variant aims to exploit error cancellations. These are exemplified by the exact compensation of all MM energies when computed on the same coordinates for the bound and for the unbound moieties. This approximation is valuable as long as there is no major conformational change in the binding site or in the ligand conformation when one has the protein or ligand alone. If major conformational changes are present, a multitrajectory approach is advisible to improve sampling.^134,135^ In this case, convergence will be more difficult to obtain because of the uncorrelated fluctuations.

The entropic contribution to the Δ*G*_bind,solv_ of eq 32 is the most challenging part to determine, although widely and freely available software (such as the Python package MMPBSA.py,^130^ using the Amber engine^136^) allows a high degree of automation in the computing process. In practice, the vibrational entropy of all species can be computed at the harmonic or quasiharmonic^130^ level from the vibrational frequencies of normal modes at local minima, identified by quenching the representative configurations for all species. The problem with computing vibrational frequencies is that one must build and diagonalize a Hessian matrix. The approach is time-consuming because filling the Hessian matrix scales as  while the diagonalization scales as , where *N* is the number of atoms. Hence, the normal modes analysis of the complex and the protein may be expensive. A saving grace in binding free energy computations is that one can assume that the entropy of the protein and the ligand do not change upon forming the complex, but this is a rather drastic assumption. Further entropy contributions come from the solvent and are approximatively taken into account by the PB and GB terms, as well as by the SA contribution. Last but not least, the protein and to a lesser extent the ligand might exist in several conformational variants, adding one last entropy term. This term can only be accounted for by extensive sampling of conformations by the full simulation in explicit solvent. A major source of uncertainty and error is associated with the presence of water molecules in the binding pocket, whose entropy variation is often not negligible and whose effect is difficult to model.

Altogether, it is not easy to definitively assess these methods, since the results depend on the details of the implementation, such as the choice of the force field and especially of the atomic charges, the PB or the GB approximation, the single-trajectory or multitrajectory variant, and the inclusion or exclusion of selected water molecules in the explicit system. As noted in Section 5.1.1, the quality is system-dependent, reflecting the different importance and partial cancellation of all the uncertainties for different chemical species.

## 5 Applications

Here, we focus on the most recent literature (approximately 10 years) on applications of small-molecule ligands. This review does not cover other drug families, such as monoclonal antibodies^1−4,137^ or biologicals, in general, because they have not been extensively investigated with computational means. For the biological targets, we focus on proteins, although compounds binding to other biomolecules (e.g., nucleic acids) also play an important role in modern drug discovery. Computational simulation and MD in particular is not so widely used to investigate these biological targets because of uncertainties in the available force fields. However, these limitations are progressively being removed.^138,139^

An important prerequisite in any computational drug discovery campaign and in a biophysical study is the availability of reliable 3D models, often represented by crystallographic structures, whose resolution should preferably be less than 2.5 Å. The availability of cocrystal structures (i.e., the structure of the crystallized drug-target complex) allows thermodynamics and kinetics simulations that start from reliable initial configurations. Combining docking with free energy methods is a more questionable strategy in terms of accuracy. However, in several real-world drug discovery scenarios, this is the only viable alternative. When protein structures are not available, homology modeling^140^ could be used instead. The idea then is that the protein sequence is available together with one or more 3D templates of homologous proteins, which allow a full geometric reconstruction of the target protein. Using these structures to initialize computations is an explored possibility of uneven success.^141^ Clearly, however, structure-based drug design performs best when coupled with solid experimental crystallographic information. Below, in addition to mentioning certain historical achievements, we review recent applications, taking 2010 as the chronological cutoff for identifying the state of the art.^142−144^

### 5.1 Absolute Binding Free Energy Applications

A major aim for computational drug discovery is the accurate and reliable determination of the binding free energy of a small-molecule ligand to a target protein. Estimating relative binding free energies across families of homologous compounds already allows researchers to prioritize drug candidates. However, only the knowledge of absolute binding free energies provides the unambiguous measure of the intrinsic strength of a binder, which is inherently related to the efficacy of the drug candidate. Absolute binding free energies, in turn, are the natural outcome of end-state free energy computations. For clarity, what follows is organized according to methods, although a strict partition is not possible, since many studies also discuss the comparison of different approaches. In each case, a few paradigmatic studies are discussed in some detail, and references to most recent papers are briefly provided.

#### 5.1.1 Validation and Applications of MM-PB/GBSA

Of the methods for computing absolute free energies, the MM-PB/GBSA class^129,145−150^ has been widely adopted for drug discovery.^151^ Their accuracy lies somewhere between fast scoring functions and more accurate methods such as FEP^152^ or potential of mean force (PMF) computations, but their low computational cost makes them a reasonable compromise.

A first validation of the method for drug discovery^151^ considered its applications at various stages of the drug discovery pipeline, i.e., for ranking ligands, for virtual screening, and for the de novo design of molecular scaffolds. After extensive testing, carried out over many ligands and eight different proteins, the authors concluded that MM-PBSA is preferable to docking scoring functions, but, because of the many residual errors in ranking compounds, it is questionable to use MM-PBSA to operatively choose a chemical substituent before synthesis. Moreover, at the level of MM-PB/GBSA discussed in ref (151), thermodynamic integration/free energy perturbation approaches are far superior. Predictions based on a single relaxed configuration were better than those obtained by systematic sampling of MD trajectories. Also, short MD runs (∼200 ps) were found to give better predictions than longer (∼500) ones. The lack of systematic convergence to a better result upon improving the various steps of MM-PB/GBSA may cast a shadow on this method’s reliability.

The accuracy of MM-PB/GBSA results and their sensitivity to the choice of force field and the sampling of configurations is further discussed in ref (153), where the authors also compare the sampling of trajectories generated with explicit or implicit solvent. This study focused on the avidin–biotin complex, since the remarkable strength of its noncovalent bonding makes it a natural benchmark. More importantly, the crystal structure is known for avidin complexes with several biotin analogues, providing a broad basis for the assessment. The Amber 8.0 package was selected to run the simulations,^153^ and the DelPhi PB^154^ solver was used to compute the continuum part of the free energy. The entropy was determined from the frequency of vibrational normal modes, while the nonpolar component was evaluated with the solvent-accessible surface area (SASA) approach, already implemented in the Amber *molsurf* module. For each complex, free energy contributions and differences were averaged over 20 configurations. The standard deviation of Δ*G*_bind_ over the 20 configurations was dominated by the entropic contribution, which is thus the major source of uncertainty. Over the seven biotin analogs considered in the study, the mean absolute deviation of computed and measured binding energies was 16 kJ/mol, with no systematic error. Poisson–Boltzmann (PB) performed better than Generalized Born (GB) as a model for the implicit solvent component of the free energy, as theory dictates. Moreover, concerning the choice of explicit or implicit solvent, the result was significantly poorer with the geometries obtained using GB as an implicit solvent model during MD runs. For instance, the avidin tetramer was not stable in this model, contrary to experimental evidence, and split into two dimers. As in ref (151), a single snapshot usually performed as well or better than several snapshots (with exceptions^153^), although the starting stage of minimizing the configuration becomes crucial for a single snapshot. Moreover, the authors found that several force fields gave equivalent results and that using a polarizable force field was not advantageous. In MM-PBSA computations, the entropic term^129^ is often assumed to cancel between reactants and products, but the major reason for setting Δ*S* = 0 is the poor reliability and time cost of this term. Extracting configurations from many short independent simulations tends to give more converged results than one long simulation. The best implicit solvent method is probably PB, yet its results depend heavily on radii and charges whose values are affected by sizable uncertainties and relatively poor transferability. It is well-known that the molecular surface, radii, and dielectric values significantly influence the reaction field energy, namely, the energy term that arises from the induced charge distribution on the molecular surface.^155^

A similar choice of many short trajectories^156^ provided results for nine inhibitors of factor Xa. Factor Xa is a protein involved in the conversion of prothrombin into thrombin. It thus affects the formation of blood clots. This work compared MM-PB/GBSA with thermodynamic integration (TI). In contrast to other studies, the authors found that GB is better than PB in this case. Once again, this observation points to the lack of unambiguous trends for a possibly overparameterized method that defies attempts at systematic improvement. This limitation should be expected, considering the number of degrees of freedom involved in protein ligand binding. Despite these drawbacks, ref (156) states that MM-GBSA performs better than TI for ligand or protein transformations that involve a change of electrostatic charge. It is known, however, that applying TI or FEP to perturbations that change the system charge requires special care.^157^ Since this technical point is not discussed in the paper, it is difficult to judge the novelty and the relevance of the statement. As a side issue, ref (156) questions the efficiency claim of MM-PB/GMSA compared to TI. However, the proposed comparison is rather uncertain, since the cost of MM-GBSA strictly depends on the implementation and protocol. More importantly, with increasing simulation time, TI converges to the exact result (up to the force field quality), while the convergence properties of any MM-GBSA are much harder to assess. A few studies even suggest that the distribution of binding free energies from independent MM-PB/GBSA measurements is broader than normal (Gaussian) and difficult to estimate correctly. Moreover, besides statistical convergence, the method has many other limitations so seems justified mainly for a quick and dirty application.

Many other assessments of the MM-PB/GBSA methods have followed in recent years (see, for instance, refs (148−150) and especially ref (146)), from which a set of prescriptions might be distilled.^146^ Despite the conceptual and practical limitations identified by the studies discussed above, MM-PB/GBSA and similar methods have been used extensively in pharmaceutical investigations. The scientific literature, which certainly does not cover all studies carried out in industrial settings, already reports a large number of applications. A comprehensive review was recently published.^146^ Here, we report further recent applications of the method and briefly discuss the possibility of extracting best practices.

Binding properties of HIV-protease inhibitors are of obvious pharmaceutical interest and have been extensively investigated, see for instance ref (134) and ref (158). Ref (134), in particular, reports a thorough retrospective analysis of all (nine) inhibitors approved by the FDA at that time. It was found that replica estimates of binding affinity, each based on a single measurement (trajectory), can differ from each other by as much as the value for the best and the worst binder. However, ensemble averages computed over many independent measurements converge to a stable value from about ∼50 replicas, each 4 ns long. The positive message is somewhat spoiled by the model’s intrinsic limitations. In this case, the model overestimates the binding free energy of the two largest binders, since it neglects the free energy cost of deforming the protein. Moreover, other data in the paper show that the variance of the 50-replica ensemble is not a monotonic function of the time duration of each trajectory in the sample. Assuming decorrelation of configurations over ∼1 ns, it is difficult to put together a fully consistent picture of the statistical and convergence properties of MM-PB/GBSA.

Ref (158) considers how mutations at the binding site of HIV protease affect its binding to four FDA-approved drugs (ritonavir, saquinavir, indinavir, and nelfinavir). Binding free energies from MM-GBSA replicated the experimental trends, but the agreement was only qualitative and with clear exceptions. The study is interesting because of the application of an approach to partitioning the binding free energy into pair contributions,^159^ attributed to residues (on the protein) and atomic groups (on the ligand). This analysis is intended to provide a rational basis for designing better inhibitors, which are less sensitive to mutations in the active site.

A popular approach for pharma companies is the use of docking followed by MM-PB/GBSA rescoring. This approach is exemplified in ref (160), where it is applied to 33 inhibitors of sirtuins (Sirt1, Sirt2, and Sirt3), which play a role in the evolution of cancer, neurological disorders, and viral diseases. The advantage of this approach is that it is systematically better than using docking scoring functions only, while still being fast. Remarkably, in this paper, the MM part was dealt with in MD simulations as short as 100 ps. Despite the short simulation time, with some preliminary tuning of model and parameters, this approach achieves fair, linear correlations with respect to experimental values.

**Figure 9:**
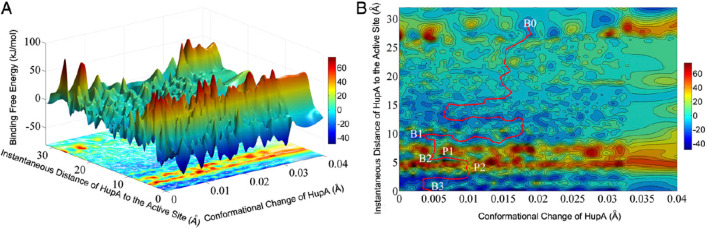
Free energy landscape of the binding process of Huperzine A against acetylcholinesterase computed by MM-PBSA along two collective variables measuring the distance of the ligand from the binding site and the root-mean-square deviation of the ligand configuration at the binding site and at the current position. The red line marks the minimum free energy path from the unbound (B0) to the bound (B3) state. Other metastable states are marked as B1 and B2. P1 and P2 are local free energy maxima. Reproduced with permission from ref (161). Copyright 2013 National Academy of Sciences, USA.

For the final study in this section, we briefly discuss a contribution^161^ that is somehow a mix of machine learning and MM-GBSA approaches. The subject of the study is the binding to acetylcholinesterase (AChE) of (−)-Huperzine A, a natural product drug for Alzheimer’s disease. The aim is to characterize not only the binding free energy but also the binding and unbinding kinetics, by computing the binding free energy landscape (see Figure 9). The free energy sampling is carried out using MM-GBSA. Analysis of the data is carried out using a combination of purpose-built in-house programs and standard grid algorithms. At first, a training stage is performed to tune the free energy surface, with the analysis giving thermodynamic and kinetic parameters in excellent agreement with the data measured during the same study. Given the limitations of the underlying MM-GBSA engine, the achieved accuracy is remarkable.

As is clear from the literature, it is difficult to identify an optimal strategy for MM-PB/GBSA, since the available studies often reach contradictory conclusions. But it is also clear that, to obtain reproducible results, different replicas are needed to get estimations within a preset error, as required by statistical mechanics. However, this might spoil the MM-PB/GBSA advantage of speed, making other more rigorous approaches such as TI/FEP more appealing. From another standpoint, the literature is not clear on whether moving from MM to QM-MM can improve the prediction ability of MM-PBSA.

The literature on applications of MM-GBSA/PBSA methods is quite broad. Here, we highlight some recent works, with particular focus on rescoring approaches. In ref (147), a variant of MM-GBSA that takes into account explicit water molecules was applied successfully to penicillopepsin, HIV1-protease, and BCL-XL systems. The work in ref (162) instead proves that MM-GBSA can detect the binding mode more correctly than docking. These simulations were done with the androgen receptor ligands phosphodiesterase 4B. The authors in ref (163) demonstrated the benefits of ensemble average rescoring of MM-GBSA for a series of antithrombin ligands. Finally, ref (164) again shows how rescoring by MM-GBSA for docking is beneficial.

Our operative suggestion is that MM-PBSA cannot be considered accurate enough for absolute binding energy estimations, but it can be an effective scoring method particularly for large virtual screening campaigns.^146^ Moreover, it can be fruitfully used in cases that are computationally too expensive for other methods. Applications of this type also include protein–DNA and protein–protein interactions.

#### 5.1.2 Applications Based on Thermodynamic Integration and Free Energy Perturbation Theory

Methods such as alchemical transformations, umbrella sampling, and metadynamics are rooted in a stronger physical basis than MM-PB/GBSA. They thus tend to be more quantitative in absolute binding free energy computations, at least when implemented using state-of-the-art atomistic force fields and explicit solvent models.

In the past ten years, there have been several successful applications of alchemical transformations (i.e., using thermodynamic perturbation theory) to compute absolute binding free energies of drug-like ligands to proteins,^165,166^ achieving an error of ∼2 kcal/mol compared to isothermal titration calorimetry measurements.

**Figure 10:**
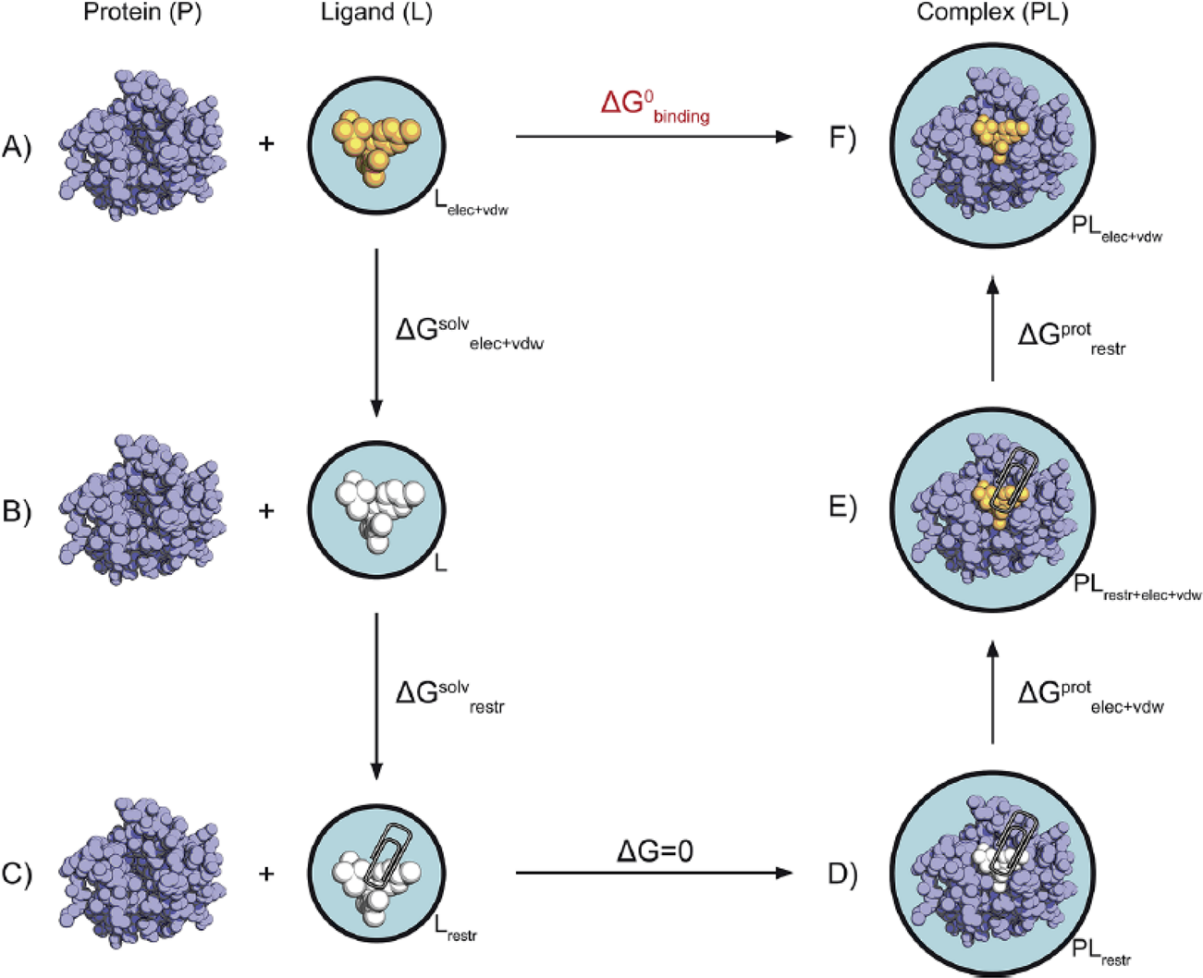
Thermodynamic cycle used to compute binding free energies by the alchemical method. The ligand is white when noninteracting and orange when interacting. The paper clip indicates that restraints are applied, as discussed in the text. The target Δ*G*_0_ of binding is indicated in red, other real or alchemical free energies are in black. Reproduced with permission from ref (167). Copyright 2016 The Royal Society of Chemistry.

A recent example of such a computation is ref (167), where the alchemical transformation was used to estimate the absolute binding free energy of 11 small-molecule inhibitors to selected bromodomains. A suitable nonphysical (alchemical) cycle (see Figure 10) is first applied retrospectively, using experimental geometries and comparisons with known experimental binding free energies, and then prospectively, starting from geometries obtained from docking computations. The retrospective stage validates the method, showing that, with the correct geometries, the error in binding free energies is of the order of 1 kcal/mol. Due to the slight uncertainty in the starting geometry determined by docking, the error of the prospective protocol is somewhat increased, but the protocol remains close to 1 kcal/mol, greatly outperforming the bare docking stage. Needless to say, the computational cost is also not comparable, since each alchemical cycle requires simulations on the microsecond time scale for explicit solvent samples of medium-large size, corresponding to ∼10^3^ – 10^4^ (state of the art 2019) core hours. These requirements exclude alchemical methods from extensive screening methods, but alchemical methods are of interest for rescoring the results of docking computations, where they provide remarkably accurate results.

The similarity of the binding pocket among homologous proteins, exemplified by the kinase family or by the remarkable conservation of the binding fold of bromodomains, raises the issue of the ligand’s selectivity. Namely, the selectivity might need to be sharpened to avoid side effects or relaxed to enhance the drug efficacy. Since differences among proteins are too large to be directly amenable to perturbative comparison, absolute binding free energies are required and provide a comprehensive solution.

The value of alchemical methods has been demonstrated in this difficult case too, as shown in a computational study,^168^ which provides a rational picture of the different selectivity of related inhibitors (Gleevec, G6G) with respect to two (Abl tyrosine, c-Src) kinase proteins. A detailed understanding of the selectivity mechanism might help prevent drug resistance by identifying the minimal mutations on the protein binding pockets that could prevent or at least greatly weaken the ligand binding.

A related broad investigation^169^ used alchemical methods to study the effect of 762 distinct mutations on the thermostability of several proteins. This work found remarkably good agreement to within 1 kcal/mol with experimental results. The dependence on the model is decreased by a consensus refinement of the results of six different force fields. As expected, better results are obtained for mutations that conserve the charge, while charge-changing mutations fare slightly worse. In addition to the force field, the residual discrepancy is attributed to incomplete sampling and the experimental error bar. This study, however, is especially interesting for biophysics and biotechnology, and only indirectly relevant to pharmacology.

Another comprehensive study of selectivity^170^ used alchemical transformations and absolute binding free energies to analyze the affinity profile of 36 complexes with the double decoupling method.^120^ The study considered 22 bromodomains from different families, in combination with three ligands (RVX–OH, RVX-208, bromosporine), comparing the computational results with isothermal titration calorimetry data. The first two ligands displayed a somewhat different selectivity profile, with RVX-208 being more selective than RVX–OH, and thus providing an ideal testing ground for the method. Moreover, RVX-208 is pharmaceutically relevant, since it is being considered in clinical trials for diabetes, atherosclerosis, and cardiovascular diseases. RVX–OH is the chemical precursor of RVX-208. This complex interplay of similarity and small crucial differences requires highly accurate methods. Since different domains are involved, relative binding free energies would hardly be computable and absolute free energy methods are the most suitable choice. As the crystal structure of the complexes was not available, docking was used to generate binding poses, followed by clustering to reduce the number of geometries to be considered. Results for both RVX ligands combined with seven bromodomains show a standard deviation from experimental data of less than 1 kcal/mol, a maximum deviation of less than 2 kcal/mol, and a high linear correlation of computational and experimental data. Compared to the results of a protocol based on machine learning,^171^ the physics-based methods have a clear advantage. The second part of the same work^170^ concerns bromosporine, which is a broad-spectrum bromodomain inhibitor primarily used in biochemistry research. The results obtained by the same protocol are somewhat less accurate than in the previous case, with a standard error of ∼2 kcal/mol, which, although relatively low, still corresponds to a factor of 30 in concentration. The linear correlation of computational results is also not as good as in the RVX case. The overall results were improved by replacing the AM1-BCC charges with RESP charges and by generally refining the charges and torsional bonded parameters, pointing to the likely cause of disagreement. In summary, the paper shows that absolute binding free energies can be computed with a sufficient accuracy to impact drug discovery, although at a high computational cost. However, results can be ligand-dependent and the role of force fields is prominent in the success of the campaign (a similar outcome was reported in ref (167) where experimental crystal structures are used instead). Additionally, bromodomains are relatively stable structures, which facilitates the free energy computation. As discussed later, flexibility has an important role in absolute free energy computations. Nevertheless, ref (170) is a reference study for this kind of computation and is complemented by a useful guide for beginners.^172^ Their protocol is also remarkable because it provides a clear example of the correct management of the ligand during the annihilation process: restraints are imposed on the ligand to avoid unbinding. Moreover, this contribution is analytically taken into account when defining the final binding energy.

**Figure 11:**
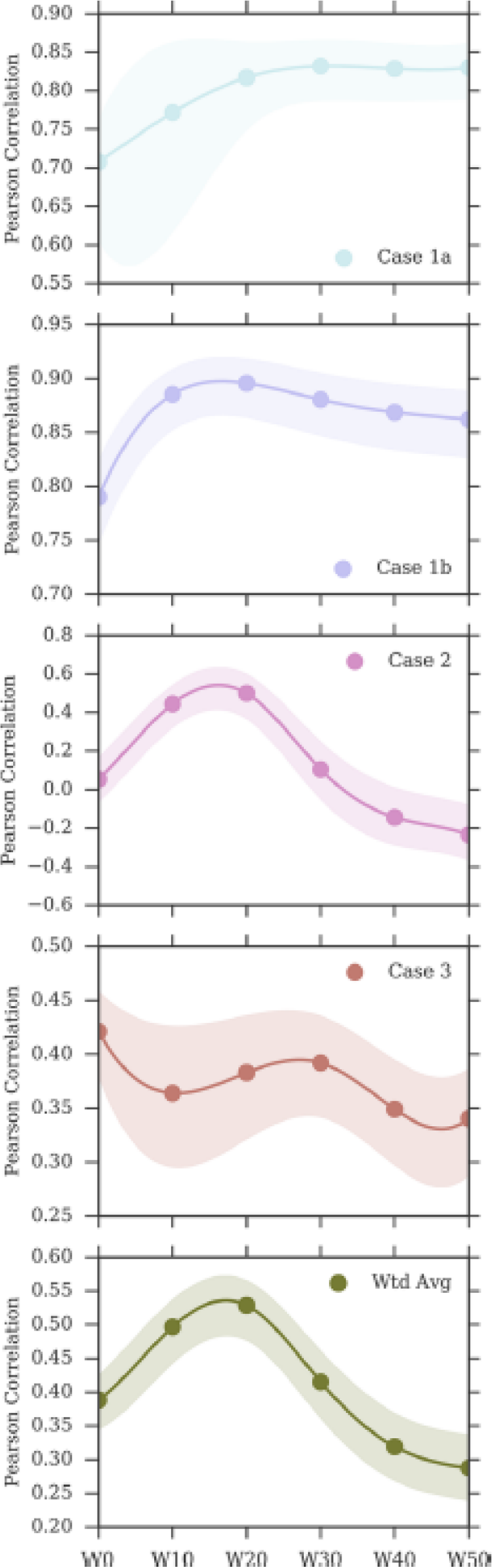
Dependence of the Pearson correlation of experimental and MM-PBSA binding affinities on the number Wn of water molecules included in the computation. Cases 1, 2, and 3 correspond to different drug-model bromodonain pairs. Case 1a and 1b differ in the pose selected for starting the computation. The improvement can be significant, but it depends on the system and on the starting point. Reproduced from ref (173). Copyright 2017 American Chemical Society.

Besides fulfilling their primary aim, high-level alchemical transformations are also routinely used to assess the quality of less expensive approaches such as MM-PB/GBSA.^168,173^ The comparison confirms and expands the assessment based on experimental data, suggesting that simpler methods, although useful in practice, are nevertheless affected by nonsystematic errors exceeding the kcal/mol scale. For instance, a systematic comparison of single-trajectory MM-PBSA and alchemical absolute free energy computations is carried out in ref (173), considering the same set of ligands and bromodomains as ref (170). For MM-PBSA, three setups are tested: MM-PBSA without entropy estimation, MM-PBSA with entropy estimation, and MM-PBSA with explicit water molecules in the vicinity of the ligand (see Figure 11 to see the effect on including explicit water molecules).

MM-PBSA was unable to consistently estimate the absolute binding free energy to within an RMSD error of about 5 kcal/mol in the best case. However, MM-PBSA with entropy estimation was only slightly inferior to alchemical approaches in terms of ranking correlation or linear correlation. This is particularly relevant because, with only 5% of the computational effort of absolute methods, MM-PBSA recovered about 90% of the accuracy in ranking terms.

In a similar spirit, an absolute free energy study^174^ analyzed in great detail the binding to albumin of ibuprofen, a widely used nonsteroidal anti-inflammatory drug. Albumin is the most abundant protein in blood plasma and is important due to its ability to transport a variety of compounds, including several drugs. As required of transport proteins, albumin presents a variety of binding sites, which can deform and adapt to ligands of different size and shape through marked plasticity. Experimental evidence (e.g., the X-ray diffraction structure of complexes) shows that ibuprofen binds to several albumin sites with a rather broad range of affinities. The complexity of ibuprofen and albumin binding is such that several experimental results may look contradictory, especially for the competing binding to albumin of ibuprofen and other compounds. Moreover, since hydrogen atoms are virtually invisible in X-ray diffraction, the protonation state of ibuprofen is not obvious, although it is expected to bind in its deprotonated anionic state, since its p*K*_a_ = 4.4 is relatively low. To shed light on the albumin-ibuprofen interaction in atomistic detail, the study in ref (174) combines docking with plain MD to identify the ibuprofen docking poses, which are then used for an alchemical determination of absolute binding free energies. More precisely, in a first stage, docking is used for an unbiased search of binding sites, exploring the entire protein volume. The number of sites retained for the following step is reduced by clustering and filtering, resulting in a total of 31 binding poses to be refined by the alchemical method. These 31 poses comprise 13 poses for neutral ibuprofen and 18 poses for the anionic form. Restraints are used during the thermodynamic cycle, and analytical corrections are introduced to take their effect into account when computing free energies of binding. The results show a systematically stronger binding for the charged form, although the unpolarizable force field might exaggerate the binding strength of charged species. The most stable bound configurations found by the computations correspond to the most stable binding sites found in experiments. Moreover, a broad variety of binding sites are found, again in agreement with the experimental results. The spatial distribution of sites, and their geometric and mechanical relation, might clarify unexplained observations about competing binding from different species.

Upon the binding of a ligand to its target, the system entropy tends to decrease for the loss of translational and rotational contributions, but the entropy balance can also change sign because of the softening of vibrational modes or due to the release of hydration water molecules. Hence, dissecting the binding free energy into its enthalpy and entropy contributions might point to the binding mechanism, possibly suggesting ways to rationally optimize a drug. Such an analysis is carried out in ref (175) considering the binding of the now-classical HIV-1 protease and two inhibitors, Nelfinavir (NFV) and Amprenavir (APV). First, agreement is demonstrated between the experimental values and the binding free energies computed according to the double decoupling method. Then, the entropy change of each reaction is estimated through the thermodynamic relation Δ*S* = −(*∂G*/*∂T*). Since *G* or, more precisely, Δ*G* is not analytically known, the derivative is computed numerically. This task is challenging, since the small difference of free energy at two slightly different temperatures is affected by a large relative error. Moreover, at variance from free energy, entropy does not satisfy a variational principle, and its determination is consequently more uncertain. Nevertheless, the results show that the binding of APV relies primarily on a favorable enthalpy change, while entropy is the major driving force in the binding of NFV. Moreover, the analysis of ref (175) shows that, in both cases, the entropy balance involves a gain due to the release of water molecules hydrating the solute, competing (and winning in the NFV case) with the entropy loss due to the ligand-HIV-1 protease interaction.

Biomolecular flexibility due to the coexistence of nearly equivalent but separate configurations connected by slow dynamical modes is a serious challenge to any simulation method based on MD, particularly to absolute binding free energy methods based on TI. The problem is also severe because it is difficult to decide a priori whether a system suffers from near-nonergodicity, and it is complicated to verify a posteriori that it has visited all relevant pockets of phase space. Since poor ergodicity can arise in each of the system components, in ref (176) this aspect is thoroughly discussed at the protein, ligand, and solvent level. Absolute binding free energies are computed according to a rather complex thermodynamic cycle, still primarily based on alchemical transformations in a combination called independent-trajectories thermodynamic-integration (IT-TI). The independent trajectory aspect, which could be seen as a surrogate of the replica-exchange method,^177^ is meant to enhance the sampling of weakly coupled basins, and it is compared to the cost of running a single longer trajectory. The influenza surface protein N1 neuraminidase and its ostelmavir inhibitor were the first target of this study. As the name suggests, the N1 protein plays a role in the influenza infection, since it favors the spread of viruses from infected cells. Its active site presents several flexible loops and is highly solvent-exposed. It is thus a suitable benchmark for assessing the flexibility effects on free energy estimates. The binding of the *Mycobacterium tuberculosis* enzyme with ligand 77074 is the second target of this study. This enzyme is important for the assembly of the impermeable mycobacterial cell wall. To validate the IT-TI method, calculations were repeated 20 times, exploring variations of the length of simulations and of the number of TI windows and testing the parallel or serial organization of the simulation. The serial organization, in principle, has the advantage of equilibrating samples in cascade. The distributions of free energies from different trajectories are reported and discussed together with mean and variance. The N1-ostelmavir complex, whose accessible phase space is more deeply divided into distinct basins, is affected by the largest standard deviation of binding free energies. The phase space of the *Mycobacterium tuberculosis* enzyme and ligand 77074 is more evenly connected, and the variance is lower. The detailed analysis allows the researcher to trace the origin of the variance in the different system components and stages of the thermodynamic cycle. Not surprisingly, the protein determines the variance with its flexibility. Somewhat surprisingly, the parallel protocol, forgoing the cascade equilibration and starting all TI steps from the same unperturbed sample, seems to be the most effective strategy, giving the lowest standard deviation of the computed binding free energies.

Finally, we briefly highlight some very recent works on this topic. First, we note that open source codes are nowadays available that support fast GPU-based thermodynamic integration, e.g., Amber.^178^ This increases the wide applicability of these methods. The work in ref (179) uses alchemical transformations to study the antimicrobial peptide microcin J25 (MJ25). This peptide is active against Gram-negative bacteria and binds to the outer-membrane receptor FhuA. This kind of work is important because it can shed light on relevant aspects of antibacterial activity. A further work of interest,^180^ which is still rare in the drug discovery community, applies FEP enhanced by a Gaussian algorithm to compute the absolute binding free energy of 7 protein targets and more than 100 ligands. The same computational study led to the discovery of a potent (subnanomolar) inhibitor of phosphodiesterase-10, which is a target considered to treat colon cancer and a few nervous system (CNS) disorders. Lastly, authors address the problem of uncertainty quantification in alchemical free energy methods.^135^

Overall, in terms of best practice, the strength and weaknesses of the different choices seem to depend on the system and on the severity of near-ergodicity. It is therefore difficult to extract a unique prescription on how to run these complex computations, with the difficulty of sampling dominating the error. In particular, the partial absence of standard benchmarks does not facilitate comparisons, even if the community is making progress here (e.g., blind competitions^181^). As always, however, collecting statistics can alleviate the problem of covering the phase space and improve overall the accuracy of predictions.

#### 5.1.3 Applications Based on Umbrella Sampling and Potential of Mean force

Absolute binding energies are free energy differences between the drug-target complex, the protein, and the ligand, whose computation can rely on a broad class of approaches. These approaches include methods based on determining the potential of mean force, as well as metadynamics, umbrella sampling, or other approaches that use the concept of a collective variable. Sampling the difference between the bound and unbound states in real coordinates instead of alchemical states has the additional and major advantage of providing kinetic information to supplement the thermodynamic free energy difference.

**Figure 12:**
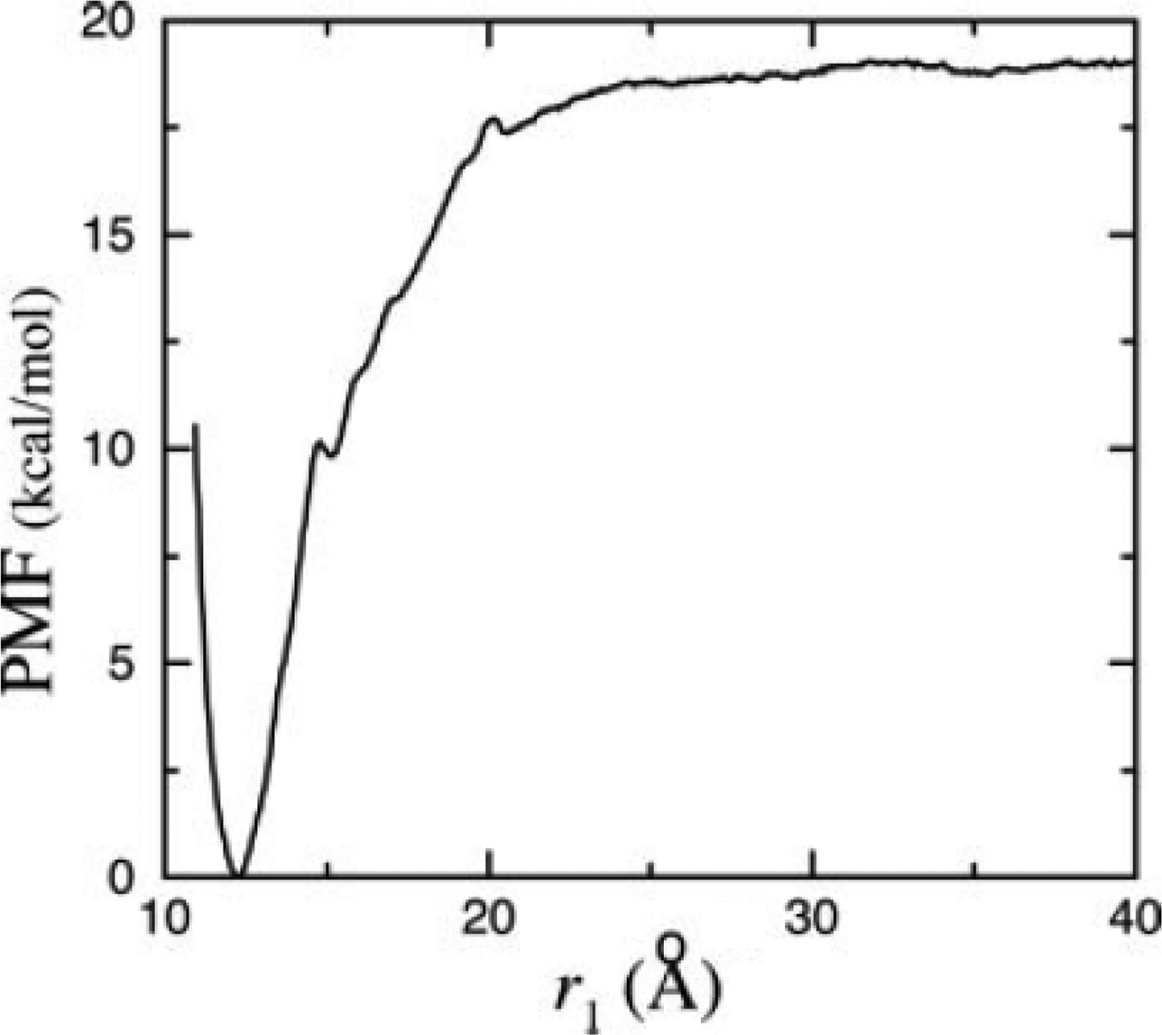
Potential of mean force computed by FEP along the distance of a peptide ligand from its binding site on the Src homology 2 domain. Reproduced with permission from ref (182). Copyright 2005 National Academy of Sciences, USA.

In ref (182), the authors propose a general method for computing equilibrium binding constants using the concept of potential of mean force (PMF). The method is validated by computing the binding free energy of the phosphotyrosine peptide pYEEI to the Src homology 2 domain of human Lck, for which the experimental binding free energy (Δ*G*_bind_ = −8 kcal/mol) is known.^183^ Src domains are highly conserved domains ∼100-amino-acids-long and found on more than 100 human proteins that, through their high affinity and specificity with respect to phosphotyrosine residues, play a role in a number of intracellular signal transduction pathways. Their regulation by peptides such as pYEEI is of pharmaceutical interest to treat cancer, asthma, and autoimmune diseases. This complex is a test case due to the peptide’s flexibility, which makes sampling by MD challenging, and due to the double charge of pYEEI, which greatly enhances the peptide’s solvation energy, complicating the application of the double annihilation method. To overcome these difficulties, ref (182) adopts a complex combination of restrained simulations and FEP steps to determine the PMF profile along a reaction coordinate (see Figure 12), which measures the distance of the ligand from its binding site on Src homology 2. In particular, the approach requires first the estimation of the equilibrium constant *K*_eq_, on top of which the standard binding free energy is computed. The computational result Δ*G*°_bind_ = −7.9 kcal/mol is in excellent agreement with the experimental value. Despite its success, the method is rather complex and requires a detailed prior analysis of the system properties, preventing the development of an automatic procedure for applying the method to large sets of compounds.

To overcome these efficiency problems, De Fabritiis and co-workers investigated the same SH2 and pYEEI^184,185^ system but developed a simpler protocol for computing the standard binding free energy from the PMF computation. First, they used steered MD to generate an initial binding path starting (in reverse) from the bound state. A second bias was applied to maintain the center of mass of the ligand in a plane going through the binding site, and a third bias was applied on the protein to maintain its correct initial orientation, still preserving the plasticity of the binding pocket. The flexibility aspect was dealt with by an ensemble average over independent simulations. A first application of the protocol,^185^ required 19 μs of aggregate simulation time to obtain a Δ*G*_bind_ = −8.5 kcal/mol, less than 1 kcal/mol away from the experimental value. In ref (185), the authors systematically optimized all the free parameters of the umbrella sampling simulations, namely, window width, number of windows, sampling time, restraints, and force constant of the umbrellas, reducing the total simulation time to a minimum of 300 ns. The standard free energy of binding was obtained from the PMF using the following expression:
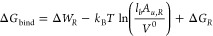
34where Δ*W*_*R*_ is the PMF free energy difference between the bound and unbound states, *l*_b_ = ∫ exp(−*W*_*R*_(*z*)/(*k*_B_*T*))d*z* is the integral of the PMF in the bound state, *A*_u,*R*_ = 2*πk*_B_*T*/*k*_*xy*_ is in the area in the plane going through the binding site, *V*^0^ is the standard volume, and Δ*G*_*R*_ is the free energy to remove the planar restraint. The ensemble average over independent and uncorrelated simulations was crucial to enhancing the convergence of the result to its final value.

In ref (186), umbrella sampling simulations were used to rationalize the different level of agonism achieved by a variety of ligands on NMDA receptors. These are ligand-gated ion channels converting chemical signals carried by neurotransmitters into excitatory electric pulses. Control of this process by compounds such as D-cycloserine or LYX-13 provides a way to treat neurological disorders. It was observed that, while a few compounds elicited a maximal response by the receptors, several other compounds acted as partial agonists, i.e., cause a lesser degree of activation. The ligand-binding domain of NMDA can be divided into two lobes with the binding site situated within the cleft. For similar receptors, there is a correlation between the degree of cleft closure and agonism level, but this crystallographic evidence is missing for NMDA. This observation called for a different and probably subtler explanation. Using two interlobe distances, the authors computed free energy surfaces (FES) for different ligands exploring a neighborhood of the binding site. The curvature of the FES at the binding site correlated negatively with the degree of agonism achieved by different ligands. One possible explanation is that free energy surfaces of low curvature are less able to restrain the ligand to the binding site, thus allowing partly open states of the channel. While this subtle aspect of FES is new, it is not surprising that partial agonism is subtle. Indeed, ref (187) recently reported on how a single bond change into a ligand can modulate the agonist behavior into the D3 GPCR. These studies confirm the complex nature of agonism/activation modulation and identify geometric features in the FES as carrier of the information that fine-tunes this process.

As already stated, information on the thermodynamics and kinetics of binding can be derived from the PMF connecting the bound and unbound states of a protein–ligand complex through a continuous free energy profile. This double capability was exploited in ref (188) to assess the relative role of two competing binding sites of adamantane-based inhibitors in the M2 proton channel of the influenza A virus, whose correct functioning is required for the virus propagation. In more detail, the M2 protein of the influenza virus A is a tetramer embedded into the viral lipid envelope whose activation leads to the unpacking of the virus genome and thus to pathogenesis. Adamantane-based ligands are an important class of M2 inhibitors. Due to acquired resistance to this class of inhibitors, new drugs are needed. Ref (188) considered the two most paradigmatic adamantane compounds (amantadine and rimantadine) together with the wild and mutant varieties of the pore, embedded into a model DPPC lipid bilayer. The goal was to explain their inhibition mechanism and analyze the acquired resistance. Simulations were started from experimental structures determined by NMR, refined by docking, mimicking a surface (S) and a pore (P) binding pose. PMF computations based on MD simulations with Gromos force fields show that the pore binding site is significantly more stable (by about 7 kcal/mol) than the surface site, as suggested by experiments. However, reaching the P site requires the overcoming of a barrier of about 10 kcal/mol, while binding to the S site is barrier-free. Hence, it can be concluded that the pore binding P is thermodynamically stable, while the surface binding S is kinetically favored. Absorption on the lipid surface might be a preliminary step to binding. To assess the role of the force field, computations were repeated using the OPLS force field. The absolute binding free energy of the two sites increased by nearly 4 kcal/mol, the barrier for the P binding decreased by 2 kcal/mol, but the relative binding energy at the P and S sites remained nearly unchanged.

In the pharmacology context, umbrella sampling and PMF computations have also been carried out to characterize the binding of small-molecule ligands to DNA.^189^ However, since this study used primarily metadynamics, its discussion is deferred to the next section.

There are some very recent applications of umbrella sampling (US) and PMF computations to determine binding free energies. One of these contributions is reported in ref (190), where the authors simulated 20 protein–ligand complexes and evaluated the ligand-binding affinity as the difference between the largest and smallest values of the free energy curve. In ref (191), US is used on the acetylcholinesterase (AChE) system using the same technique as in the previous study to estimate the binding free energy of about 40 noncongeneric ligands. In ref (192), in contrast, the extended adaptive biasing force (eABF) is used to estimate the PMF. Finally in a comparative analysis^193^ for host–guest systems, US is systematically compared to the double decoupling method. The results show that the two methods are highly correlated, even if they return slightly different results. This aspect, namely the consistency of the methods’ results is important for their systematic use in industry.

#### 5.1.4 Applications Based on Metadynamics

Another notable class of protein–ligand binding protocols is based on metadynamics,^72^ of which there are many variants.^84,194−196^ Gervasio and collaborators^197^ were the first to use well-tempered metadynamics with the path collective variables of Branduardi^198^ (see also ref (199)) to compute the binding free energy of a protein–ligand complex. Given any regular sequence of configurations joining the bound and unbound state of a complex, the first of the two collective variables of ref (198) measures the progression along the reaction, while the second measures the distance of the actual path from the arbitrary initial path, thus defining a tube from reactants to product. This last aspect is used to enhance efficiency, simulating the unbinding process instead of binding, and limiting the sampling to a tube leading the ligand to the bulk solvent. The method was applied to investigate the binding properties of a homologous series of five 2-anilino-4(hetero) aryl-pyrimidine derivatives to cyclin-dependent kinase, CDK2. First, a putative binding path was obtained via undocking. Then, the path was optimized by computing the free energy in the 2D space of the collective coordinates. The total computational time for each metadynamics run (one ligand) was between 40 and 200 ns. During this sampling, several docking and undocking events were observed, putatively indicating convergence. To compute the free energy difference, the authors used the difference in the PMF between the bound and unbound state. Results for the five ligands displayed a remarkable agreement with the experimental data. Upon correcting for the standard state volume, the largest error was 1.2 kcal/mol. In four of five cases, it was 0.5 kcal/mol at most. If the sequence of configurations chosen to represent the unbinding path is far from the optimal sequence, the free energy profile might deviate from the true one. However, even in this case, there was no effect on the final ΔΔ*G* measuring the activation energy required for the ligand unbinding (thus related to *k*_off_). This demonstrated that the combination of metadynamics and a path collective variable could correctly capture the state variable property of free energy. A similar strategy, also requiring path collective variables, was used in ref (19) to investigate the binding mechanism to purine nucleoside phosphorylase.

**Figure 13:**
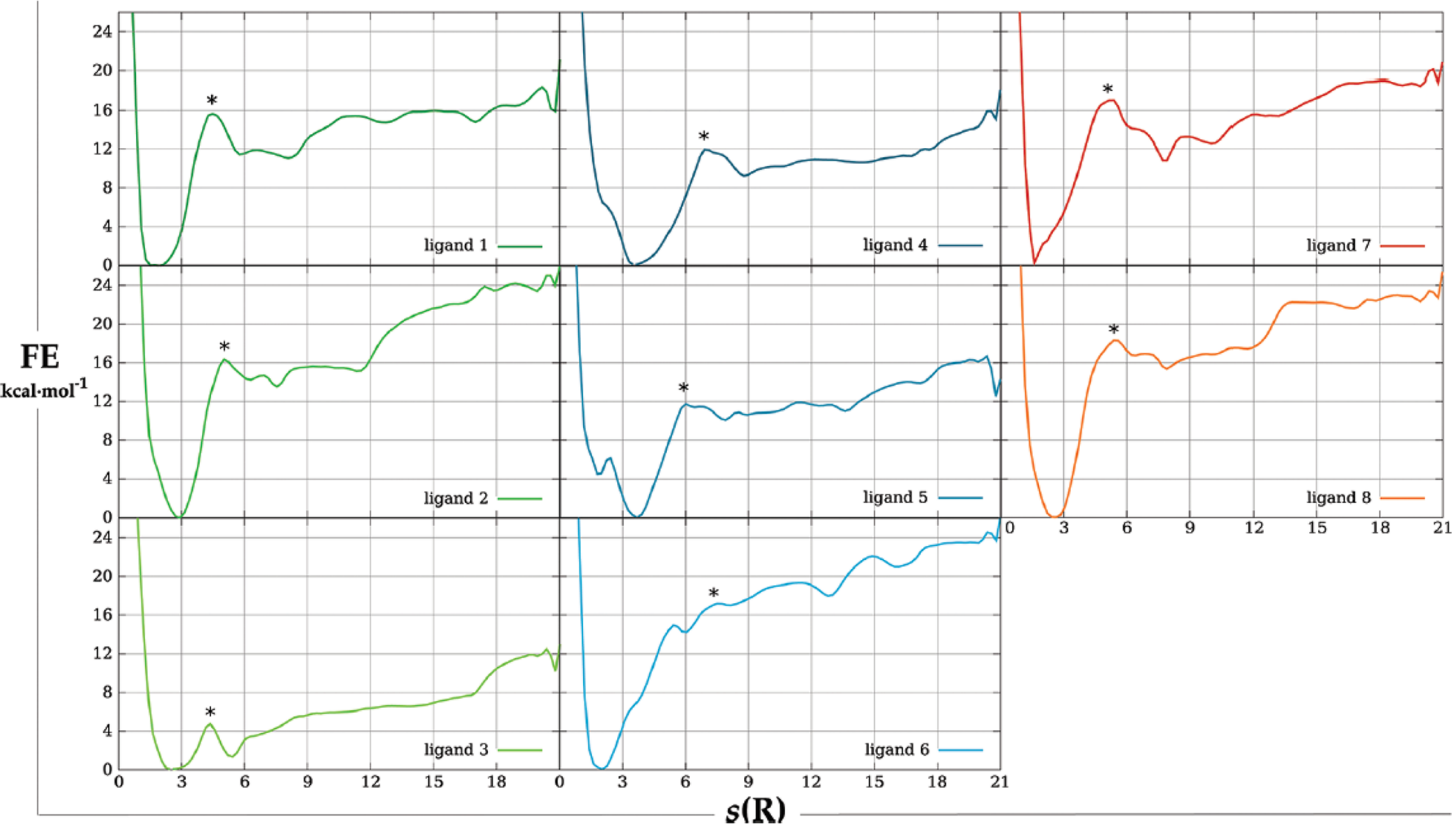
Free energy profiles, computed via metadynamics, along the unbinding path for 8 inhibitors of the MAPK p38 kinase protein.^200^ The *s* collective variable measures the advancement of the binding process. The star (*) marks the transition state between bound and unbound. Reproduced from ref (200). Copyright 2012 American Chemical Society.

The protocol of ref (197), tuned and improved, was used in ref (200) to compute binding free energies for the kinase protein MAPK p38, which, like other kinases, participates in cellular signaling processes. Its regulation might have a positive role in cancer therapies. Ref (200) considered eight inhibitors of MAPK p38, defined by slight side chain variations of a single basic scaffold. Once again, the unbinding process (not the binding process) was simulated. The major improvement was the development of an unsupervised approach, based on collective variable and multiple walkers, which could be scaled to computations involving many ligands, minimizing human intervention. Automatic procedures were implemented to analyze the results and, in particular, to identify an approximate transition state from which a *k*_off_ parameter could be estimated. To compute the binding free energy, one first attributes a range of ligand-binding pocket separations to the bound and unbound state, respectively. This is easy for the bound state, since the minimum of the PMF is clearly identified (see Figure 13). It is less easy for the unbound state, since in this case the PMF has oscillations and fluctuations at long-range too. Then, the free energy of the two states is determined by averaging over the two ranges, and Δ*G* is computed as the difference of these two values.

On the basis of previous formulas,^200^ the authors proposed a free energy correction to account for differences of concentration in the simulation from the standard concentration *C*_0_ = 1 M. However, the uncorrected approximation already gave a good correlation with experimental values.

The path method is appealing despite the following potential drawbacks: first, it requires prior knowledge of one binding/unbinding path. Second, regarding the path collective variable,^198^ one must get nearly equidistant frames in the RMSD space to define the path. This has not been completely solved automatically (physics-based) or, at least, there is no widely available solution. These technical aspects might restrict the use of this valuable approach.

The picture of a binding funnel in phase space driving a ligand and a protein toward their bound state is a popular notion in the statistical mechanics of biosystems.^201^ A reversed geometrical funnel, broader at the binding site and narrower toward the solvent, is the defining concept of funnel metadynamics, a widely used protocol,^202^ whereby metadynamics is combined with a funnel-shaped restraint to reduce the phase space explored by the ligand in the unbound state. The broad cross-section of the funnel at the binding site is meant to allow full freedom in the exploration of bound configurations. The effect of the funnel bias on the free energy estimation is removed a posteriori through an analytical correction (see eq 3 in ref (202)). The method has been successfully applied to the benzamidine/trypsin system and the SC-558/cyclooxygenase system. The first system is a widely studied model of binding kinetics and thermodynamics,^16^ and the second system concerns a protein (cyclooxygenase, COX-2) involved in inflammation and pain and one of its selective inhibitors (SC-558).

It is interesting to compare the path-based^197,200^ and funnel approaches, since both are built around metadynamics. For the path-based approach, one must first build an approximate unbinding/binding path, identified by nearly equidistant frames,^198^ and then run metadynamics. Then, the path approach allows the collective variable (CV) to naturally emerge from preliminary simulations. Interestingly, these preliminary simulations may require the definition of CVs, which appears to be a contradiction. However, the choice of collective variable for a preliminary binding/unbinding run is much less critical than the choice of the CVs for the full free energy calculation.

In the funnel approach, a preliminary unbinding/binding path is not needed, although one must still define the correct collective variables. However, the funnel approach can directly deliver the free energy.

Both path-based and funnel methods are valuable. However, in our opinion, the path approach is probably superior since it does not need a finetuned reaction coordinate to start and requires less supervision and could be automated. Thus, if one uses a reliable default CV-based method^25,203^ to generate an initial unbinding/binding path, then the path approach becomes extremely appealing. Conversely, if the CVs can be reasonably identified then the funnel approach is more directly applicable. For real-world blind drug discovery, we expect the two-step path-based approach to be more widely applicable. In particular, it should be possible to combine dynamic docking/undocking^71,203−205^ with this path-based approach to obtain reasonable paths and accurate free energy estimations. Recently, the path method has been combined with MSM, which may represent a further way to provide an initial path, despite the fact that the number of intermediate configurations could result in less than those needed for accurate path-based free energy calculations.^206^

A somewhat different case of binding is the intercalation of small-molecule drugs between two base pairs of DNA, disrupting its replication and causing apoptosis of cells. This chain of events is behind the action of a few anticancer drugs.^207,208^ The thermodynamics and kinetics of the anticancer drug daunomycin’s intercalation in DNA was investigated by metadynamics in ref (189). Three independent collective variables were introduced, describing the distance and orientation of the daunomycin ligand with respect to its intercalation site. MD sampling lasted 110 ns. The resulting free energy landscape shows several minima underlying a three-step intercalation process, starting with the barrier-free binding to the minor groove, an activated rotation into an intermediate site, separated by a small free energy barrier from the final intercalated pose. The computation of a PMF profile through umbrella sampling gave similar results. The agreement between methods is important, and it is a strong assessment of the reliability of the calculations. Notably, metadynamics has been run using several collective variables. This is not easily achievable with umbrella sampling whose complexity scales poorly with the number of variables. Indeed, in this paper, umbrella sampling was used considering only one collective variable. In general, this is the setting where umbrella sampling simulations are advisible, whereas metadynamics are slightly less affected by this issue. Nevertheless, to avoid convergence problems, it is good practice in metadynamics to use no more than two collective variables. Overall, metadynamics is a very powerful and recommended technique for protein–ligand binding studies.

Very recent applications of metadynamics for protein–ligand binding include a variety of target proteins like GPCRs,^209,210^ the lysozyme,^211^ the human neuroreceptor M2,^212^ the kisspeptin receptor,^213^ the NOP receptor,^214^ trypsin-benzamidine^23^ and the vasopressin receptor,^215^ as well as model host–guest systems such as β-cyclodextrins.^216^

#### 5.1.5 Applications Based on Steered MD

This method (SMD) was used for the first time in the ligand design context^217^ to classify a set of flavonoid inhibitors of the β-hydroxyacyl-ACP dehydratase complex (a two-chain dimer) of *Plasmodium falciparum* as active or inactive. To this end, starting from the experimental structure of the protein, each of the ligands was positioned in the best binding pose on the protein found by docking, followed by clustering. The strength of the protein–ligand interaction was probed by pulling the ligand out of its binding site by SMD, with a reaction coordinate given by the distance of the time-dependent center of mass of the ligand from this same center of mass at time *t* = 0 in the binding pose. The raw data provided by the simulation is the histogram of the required pulling force as a function of the reaction coordinate. Hence, the measure of the binding strength is a combination of the force intensity and the range of the corresponding interactions. While the method is not so quantitative as to provide an accurate scoring of compounds, the difference in the force versus distance histogram is sufficient to discriminate active (i.e., strongly bound) from inactive (i.e., weakly bound or even unbound) compounds. This provides a better assessment of the compound’s potential as a drug, fully taking into account the flexibility of the protein and ligand. The insights from this process led the researchers to propose a new inhibitor, whose experimental activity confirmed the computational prediction. The approach is simple and can be easily automated.

The same method was used in ref (218) to assess the binding strength of nine ligands, organized into two subgroups, of the cyclin-dependent kinase-5 (CDK5) enzyme, which promotes the hyperphosphorylation of the tau protein, and thus could be a target for drugs to treat Alzheimer’s disease, multiple sclerosis, Parkinson’s disease, amyotrophic lateral sclerosis, etc. Kinase proteins are a difficult target for steered MD because of the simultaneous presence of a solvent-exposed active site and marked flexibility at the same binding site. Once again, the method is unable to quantitatively rank compounds of similar binding affinity, but it was nevertheless able to discriminate between active and inactive compounds.

The human version of the mouse double minute protein 2 (MDM2) interferes with the tumor suppression activity of the TP53 protein. It is therefore a suitable target for inhibition in order to enhance the innate anticancer defense of the organism. The binding free energy of four such inhibitors of MDM2 was estimated in ref (219), using a combination of Brownian dynamics and SMD and using the fluctuation–dissipation theorem to map the free energy landscape in the vicinity of the binding site. The collective variable for the SMD simulation was represented by the parallel displacement of two selected atoms on the ligand during unbinding. The full trajectory from bound to unbound was divided into 16 segments and each segment probed in both (unbinding and binding) directions. If *A* and *B* are the end points of each segment, the free energy variation Δ*G*(*r*) at a generic point *A* ≤ *r* ≤ *B* is computed as
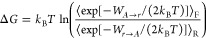
35where F/R are forward and reverse pulling simulations, brackets indicate average, and *W* is the work on the system. An excellent agreement between the experimental data and the computational results was achieved. A corresponding set of binding free energies was computed by MM-PBSA. A comparison with experiments and with steered MD demonstrated the superiority of the latter with respect to MM-PBSA. MM-PBSA indeed is more advisible for a quick scoring, whereas repeated steered MD is a more rigorous way to address the free energy computation problem. Nevertheless, steered MD is an out-of-equilibrium method, and several repeated replicas are often needed to converge the free energy. Other methods such as umbrella sampling and metadynamics are probably more advisible as there is no strong evidence that using steered MD is systematically advantageous and equilibrium methods should be the default choice. Steered MD could be used for getting an initial path, whereas metadynamics and umbrella sampling can be used to compute the free energy along this initial guess.

Recent works have used this technique to study the following targets: focal adhesion kinase,^220^ the cancer target LSD1,^221^ neuraminidase,^222^ FK506 binding protein together with trypsin and cyclin-dependent kinase 2,^223^ xylose permease,^224^ and enzyme 5-enolpyruvylshikimate 3 phosphate synthase.^225^

Summarizing this section, absolute binding energy computations are now computationally feasible, up to the accuracy limit of existing force fields, and of finite sampling of the relevant phase space. As expected, computations are more reliable for relatively rigid systems, whereas very flexible systems pose severe sampling problems. Methods like MM-PBSA are not quantitatively accurate, at least in current implementations of the method. Double decoupling with proper restraints, umbrella sampling, and particularly metadynamics are powerful techniques, being computationally more expensive but also much more predictive than MM-PBSA.

### 5.2 Relative Binding Energy Estimation

As emphasized in the previous section 5.1, determining the absolute binding free energy is the most comprehensive way to quantify the ability of small-molecule ligands to bind and thus affect target proteins. Nevertheless, from a practical standpoint, knowledge of relative binding free energies is already highly valuable, providing crucial guidance and insight for the lead discovery and lead optimization stages of drug development.

From the computational point of view, the rationale of focusing on relative binding free energies is that, as already explained in the theoretical section, a convenient thermodynamic cycle can be used. A further opportunity to optimize the time and accuracy is provided by spanning an extended set of compounds moving along molecules of maximum (sub)structural overlap, minimizing the sensitivity of the overall picture on the individual steps. This maximum-overlap strategy is easily integrated into computer approaches to generate new drug candidates.^226^ An additional appeal of relative binding free energies is that several systematic errors could be canceled in the comparison of different cases, opening the way to cheaper models, less stringent computational protocols, and broader searches. The underlying methods are largely those detailed in section 5.1, which, with the exception of MM-PB/GBSA, already target free energy differences.

In this respect, free energy perturbation (FEP) and thermodynamic integration are two powerful techniques extensively used to compute free energy differences. The Zwanzig equation is central to FEP.^227^ In 1985, William Jorgensen^227^ was the first to report the mutation of methanol to ethane. In that paper, Monte Carlo was used for sampling. Nowadays, molecular dynamics is more often used. Nevertheless, Jorgensen’s paper included the typical issues and checks that arise when FEP is applied to ligands in a pharmaceutical context. In particular, convergence checks, the subdivision of the 0 ≤ λ ≤ 1 interval into windows, and simulations run in both directions emerged as the main aspects to carefully monitor when running FEP. This paper used the TIP4P^50^ model of water with the OPLS force field parameters.^28^

#### 5.2.1 Application of FEP to Directly Compute Binding Free Energy Differences

The first application of FEP to compute relative free energies of biomolecules was reported in 1986 (McCammon^228^), achieving agreement with experimental data for the difference in binding free energy of two benzamidine inhibitors of tripsin and for benzamidine for native and a mutant trypsin. In 1987, Kollmann and co-workers^152^ reported another estimation of the free energy difference of protein–ligand binding. For the thermolysin enzyme and a pair of phosphonamidate and phosphonate inhibitors, they found a difference in binding free energy of 4.21 kcal/mol versus the experimental value of 4.1 kcal/mol. In addition to the immediate interest of its quantitative determination of relative binding free energies, this work is also important because it made available a general-purpose FEP implementation within the popular Amber package,^136^ thus greatly promoting the dissemination of FEP within the computational community. Despite the positive impact of these pioneering works, FEP did not immediately become an industry standard in drug discovery, mainly because of its high computational cost but also because of occasional poor convergence of FEP, especially with explicit solvent models, limited accuracy and transferability of force fields, and incomplete coverage of small-molecule species by widely available force fields.^229−232^

The Jorgensen group provided a medicinal chemistry success story for FEP.^233^ With the use of the Monte Carlo FEP algorithm, a 5 μM non-nucleoside inhibitor of HIV reverse transcriptase (RT) was improved into a highly potent 55 pM drug.^233^ The FEP/MC stage of the lead optimization relied on OPLS force fields for the protein and the ligand, together with TIP4P^50^ water. In contrast to current practice with MD, the protein backbone was kept fixed. Protein flexibility, however, is known to be crucial in several cases, and the success of the rigid-backbone setup of ref (233) is likely to be an exception. The comprehensive study included a docking and scoring stage on a two-million-compound library and was guided by the X-ray structure determination of a number of small-molecule crystals and of complexes of RT with analog compounds. The FEP-driven lead optimization focused primarily on a single compound and was made easier by the knowledge that non-nucleotide RT inhibitors bind to an allosteric pocket ∼10 Å away from the RT active site. The potency of the lead compound variants was experimentally measured by the EC_50_ dose required to protect 50% of infected cells, represented in this case by MT-2 human T-cells.

More recently, researchers significantly expanded the scope of FEP/TI calculations, especially for relative binding free energy studies. First, sampling was improved by fast and relatively inexpensive GPUs and codes to efficiently exploit them.^234^ This currently provides at least 1 order of magnitude of acceleration with respect to CPU implementations, measured at equal accuracy and comparable cost. Second, force fields became more generally applicable, thanks to (among others) recent versions of CHARMM,^32^ OPLS,^35^ and Amber,^30^ especially in its GAFF extension,^34^ covering a wide variety of organic small molecules. Several works demonstrated the reliability and efficacy of the approach.^235−237^ An optimized version of FEP supplemented by Hamiltonian replica-exchange and solute tempering (FEP/REST, see section 4.3) was particularly effective in overcoming quasi-nonergodocity conditions and the challenge of explicit solvent.^122^

The computation of the protein/ligand relative binding free energies [benzene and *p*-xylen with the L99A mutant of the T4 lysozyme, as well as two closely related but flexible ligands with thrombin (Factor IIa)]^122^ was first applied to compare the performance of FEP/REST to that of bare FEP, emphasizing the ability of FEP/REST to overcome free energy barriers, and to account for sizable structural reorganization in the protein or ligand. More recently, researchers conducted a retrospective assessment of FEP/REST for relative binding energy determination on a set of 200 ligands and 10 targets.^121^ This work demonstrated broad applicability to lead optimization. At the validation/retrospective stage, both ref (122) and ref (121) emphasize the role of improved force fields^238^ in achieving high-quality results.

**Figure 14:**
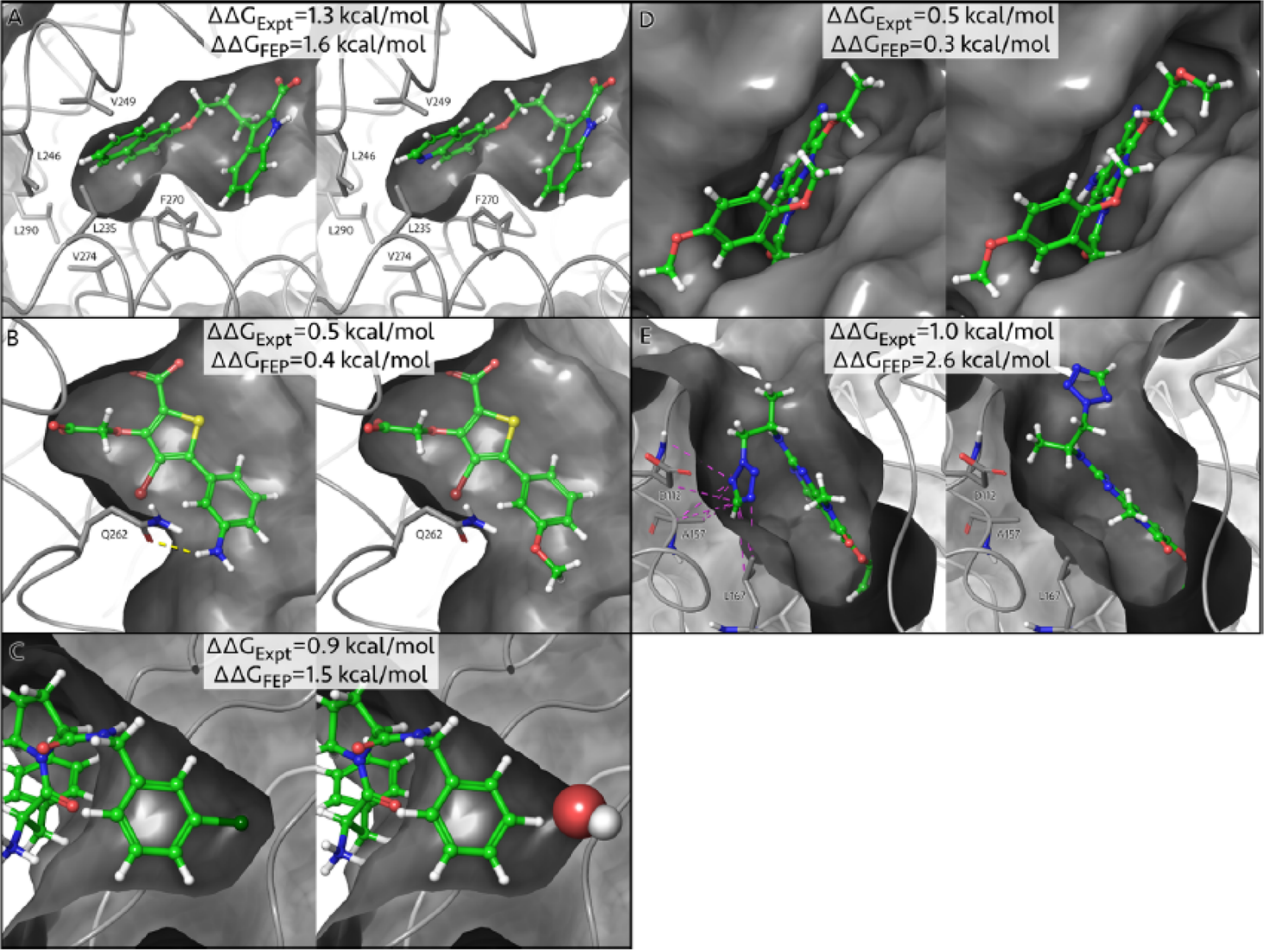
Structure–activity patterns determined by FEP for ligands of the induced myeloid leukemia cell differentiation protein Mcl-1. Differences of binding free energies (ΔΔ*G*) between pairs of compounds compare favorably with experimental data. Reproduced with permission from ref (121). Copyright 2015 American Chemical Society.

More specifically, in ref (121), several FEP/RESP computations were carried out with the Desmond MD engine,^239^ optimized for GPUs. This Desmond version can perform four perturbations per day on eight Nvidia GTX-780 GPUs, which is a great improvement on the CPU-only version.^121^ An automated workflow with a graphical user interface simplifies the definition of the transformations needed. In total, 330 perturbations were performed, many of them covering the change of up to 10 non-H atoms, with an absolute error of less than 1.5 kcal/mol in 81.2% of cases. The approach was thus validated for a real-world hit optimization campaign (see Figure 14 for selected successes).

The result is remarkable, considering that the inherent experimental uncertainty can be estimated at 0.4–0.7 kcal/mol for each transformation.^121^ Moreover, a much higher correlation was obtained with FEP compared to the MM-GB/SA^240^ and Glide SP^241^ scoring methods. In the prospective stage of the same study, FEP/REST was applied to inhibitors of IRAK4 and TYK2 with remarkably accurate results.

The protocol in ref (121) was not the only attempt to automate the FEP/TI framework. Christ and Fox from Boehringer defined an automated TI framework for Amber 11 (an open-source MD code) with significant results.^242^ The framework was systematically applied with 92 ligands binding to five different targets, including six different ligands of the Mouse Major Urinary Protein and 32 substrate analog PDE5 inhibitors.^242^ Several other compounds were used, including bromodomain inhibitors. The automated procedure was implemented using the OEChem toolkit.^243^ It takes into consideration the maximum common substructure of all pairs of ligands in order to define a maximum spanning tree that minimizes the total number and the strength of perturbations. Then, all the topologies are automatically computed and written to start the simulations. Considering the whole set of windows, the total simulation time was around 50 ns per binding free energy comparison. Postprocessing was performed with TI or with the Bennett acceptance ratio method to multiple states (MBAR^111^), with a negligible difference. To estimate the statistical error bar, computations were replicated for each ligand-protein pair, starting from different initial configurations. A nonnegligible difference of the two results was found, quantifying the dependence of the results on the choice of starting configuration, thus pointing to insufficient sampling. Moreover, the authors did not find apparent correlation between the size of the perturbation and the statistical error bar, confirming that the separation of the relevant phase space into nearly disjoint basins is the major source of error. For PDE5, the second system, the results were not so encouraging, since a relatively poor correlation was obtained with respect to experiments. Since the protonation state of the ligands is not certain from X-ray structures, these computations were also repeated twice, considering both the neutral and the protonated state of each compound. Both series of binding free energy differences displayed a similar deviation from the experimental values, leaving the determination of the protonation state unsolved. At any rate, the inclusion of charged ligands in the computation is an additional challenge, and it is known that specific countermeasures need to be taken into account^244,245^ with charged moieties.

A very recent contribution to the high-throughput screening of compounds was reported in ref (246), defining an automated workflow for systematic relative binding free energy computations. The protocol, dubbed QligFEP and implemented in an application programming interface, is based on FEP in its double topology variant. It exploits the open-source MD engine Q^247^ and uses the most popular force fields such as OPLS, CHARMM, and AMBER. The FEP capability is complemented by modules that implement linear interaction energy (LIE) and empirical valence bond (EVB) schemes. The strength of QligFEP is its ability to set up series of FEP computations, using the concept of maximum structural overlap, and drastically decreasing the need for expert supervision. Ref (246) is primarily devoted to extensively and successfully validating QligFEP by comparing it with FEP data from previous studies. The results reported in the paper concern simulations with the complex embedded into a finite droplet of water solvent. Nevertheless, periodic boundary conditions are implemented in QligFEP.

In ref (248), FEP was used to reanalyze the binding of a series of amino-adamantane inhibitors to the same M2 (proton channel) protein of influenza A virus that had been investigated with PMF computations in ref (188). Exploiting the relative binding free energy framework, the analysis was extended from 2 to 11 inhibitors. This study also covered the determination of the binding mechanism and the analysis of the different factors (Coulomb, dispersion energy, and solvation) that decide the pose of each ligand within the M2 pore. As crystal structures were not available, a preliminary docking phase was carried out. This step is crucial because an incorrect pose may lead to incorrect results. In general, this strategy significantly increases the overall uncertainty of predictions, and it might not be advisible in real-world blind studies. In this retrospective study, however, the docking plus FEP approach worked well. To probe the effect of protein flexibility, two sets of FEP calculations were carried out. In the first set, the protein backbone was restrained. In the second set, the backbone was flexible and the protein was embedded in a DPPC lipid bilayer, providing an idealized model of biomembrane. Results from the rigid-backbone stage correlated poorly with experimental data, while the second set of results displayed the usual good correlation of fully fledged FEP computations. The comparison confirms the important role of protein flexibility. However, one cannot draw an unambiguous conclusion because of the lack of information on how the effect of the backbone restraint was removed from the results. Detailed analysis of the results shows that the binding free energy of ligands within the pore reflects the fine balance of hydrogen bonding, Coulomb, and dispersion forces. An important if not decisive role is played by the dehydration of ligands required to enter the pore.

The cyclin-dependent kinase CDK2 had already been investigated by replica-exchange FEP in ref (197). It was considered again in ref (249), which used FEP/REST to compute the relative binding free energy of 10 ligands. Comparison with the results of plain MD/FEP shows that FEP/REST greatly improves the sampling of multiple free energy basins, leading to faster convergence with relatively short (a few ns) MD runs. A further FEP/REST study using Desmond (MD-based) and the MCPRO package (MC-based) again retrospectively analyzed the binding of non-nucleoside inhibitors of the HIV-1-reverse transcriptase, quantifying the effect of mutations on relative binding free energies. The results confirm the ability of FEP/REST to provide valid indications for lead optimization. Moreover, there is fairly good agreement between the predictions provided by MD-based and MC-based FEP/REST implementations, with the computational cost on GPU hardware being limited to a few hours per perturbation. Discrepancies between the two implementations, in particular, were at the acceptable level of 1 kcal/mol in most cases, despite the use of different force fields and different accounts of the protein backbone flexibility.

A recurring observation in recent works on free energy computations is the important role of water molecules. Besides ref (248), several early studies (see, for instance, refs (250−253)) found that water molecule networks can significantly influence free energy estimations and so potency predictions. Algorithms have been implemented to create^254^ and score^255,256^ water networks, and they are extensively used to identify hot spots on the protein surface that are suitable for hydrophilic and lipophilic complexation.

Because of the unavoidable incompleteness of sampling, the water structure created to initialize MD simulations might affect the estimate of the binding free energy. It is thus important to match proteins, ligands, and their complexes with a nearly optimal water network. This is discussed in detail in ref (257), which used FEP to run an in silico campaign on 17 inhibitors of the p38α MAP kinase. This data set mimics a typical lead optimization medicinal chemistry scenario, in which a congeneric series of compounds is analyzed to find the optimal substituent of a chemical group (here, a benzene ring). This lead optimization exercise demonstrates the importance of the initial placement of water molecules around the ligand for FEP calculations. In all computations, the solute structure was derived from X-ray diffraction on a lead complex. In a first attempt, a popular default strategy used by many tools to set up simulations was used to embed the solute into a finite droplet of water. This was obtained by replicating a small water seed into a larger aggregate, removing water molecules whose distance from any nonhydrogen atom in the solvent was less than a preassigned cutoff or whose position was outside the droplet radius. There was no further optimization of the water placement before the free energy determination. In a second series of FEP computations, a water droplet of the same radius was created by a more refined algorithm implemented in the JAWS software,^254^ supplementing a slightly more refined initial placement with a preequilibration step for the solvent only. Despite a fairly long FEP stage, the estimated relative binding free energies still reflected the different starting choice. In particular, the difference between the relative binding free energies obtained with the optimized water placement and experiments is much smaller (reduced by half) than the difference between the data from the first set (simple water placement) and experiments. Analysis of configurations showed that part of the advantage is because JAWS can correctly populate cavities. Residual inaccuracies are attributed to incomplete sampling of the protein structure, possibly due to flexibility.

Another broad subject for FEP/TI computations of relative free energies is the variations on the protein side of the ligand-protein complex. In particular, not only it is possible to run FEP/TI computations modifying the ligand, but the same strategy can be adopted to analyze the effect of variations in protein residues,^258^ maintaining or varying the ligand. This is a way, for instance, to gain insight into the effect of point mutations on the ligands’ binding, as briefly considered in previous examples. Considering again the protein side, it is also important to study the sensitivity of FEP/TI binding free energies to protein reorganization, which could be due to induced fit or to conformational selection (see section 5.3). Below, we analyze two recent papers that address these important issues.

One study (see ref (259)) considered the adenosinic receptor GPCR (A_2*A*_) by computing the binding energy variation for an agonist (N-ethylcarboxamide adenosine, NECA) and an antagonist (the triazolotriazine derivative ZM241383) ligand while the receptor underwent alanine-scanning, that is, mutating a sequence of residues to alanine one by one. The A_2*A*_ was simulated embedded inside a model POPC membrane. The computational results along the sequence of mutations (17 for NECA, 14 for ZM241383) were compared with saturation assay experiments with a reference radioligand. In these saturation assay experiments, the experimental binding affinities along this series were measured based on their ability to compete with the radioligand, thus obtaining ratios between the mutated activity and the wild-type. Such an experimental and computational analysis can support site-directed mutagenesis or the design of personalized drugs for rare diseases. The relative binding free energies computed by FEP display a good agreement between experiments and computations. A few failures in the case of agonist ligand NECA might be explained by the only partial activation of the simulated complex, since the G-protein intracellular component is missing from the model.

In general, dealing with GPCR is challenging because minimal changes in the ligand or in the residue side chain may result in sudden changes of binding affinity, sometimes giving rise to near-discontinuities (activity cliffs), which are difficult to capture computationally.^187^ This scenario might be complicated further by the active or inactive state of the GPCR itself. This implies that it is risky to seek correlations based on FEP/TI calculations on GPCRs, particularly when the reference experimental values (in this case mutations) are not obtained under ideal conditions.

Another study (see ref (260)) discusses the common challenge of changes in the protein conformation when it comes to using FEP to accurately determine the relative binding free energies. Such changes occur fairly often during the alchemical transformation of ligands. A suitable test case to investigate this issue is provided by a mutant of the T4 lysozyme (L99A), a molecular target that has been extensively characterized by experiments. The feature of interest is a small apolar binding site, which can accommodate a variety of neutral ligands. According to the size of the ligand, the protein binding site may adopt one of three conformations upon binding: closed, intermediate, or open. The mutual interconversions of these conformation are activated processes and occur only rarely. Ref (260) focused on determining the relative binding energies for a sequence of congeneric ligands, starting from benzene, and growing a saturated chain on one of its carbons up to hexylbenzene. The FEP implementation with a standard REST setup,^122^ based on relatively short (5 ns) sampling of the λ windows, achieves only moderate accuracy. This appears to be due to the difficulty of sampling the three relatively disjoint conformations. The error in the relative free energies can be as large as 5 kcal/mol, making the computation unreliable. This is particularly worrisome, since the size of the structural rearragement is limited to 3.5 Å at most. To mitigate the problem, the parallel tempering hot spot, in which temperature is artificially raised, is enlarged to include a small portion of the binding site. This quickens the rate of conformational sampling and improves the situation, without fully solving the problem of accurate sampling. The simplest and most effective solution is to increase the sampling time from 5 to 55 ns for each λ window. This time increase is sufficient to sample a few changes of configurations, and restores the consistency of the results.

The analysis carried out in this study suggests that perturbative methods such as FEP and TI may not be adequate for big conformational changes but that absolute free energy methods are more suitable in these cases. A further field where FEP/TI may not be fully adequate is fragment-based drug discovery. Here, the large size difference between ligands and active pockets may hamper a proper convergence of this kind of simulations and eventually impact free energy difference predictions. Nevertheless for pure correlative and relative free energy studies, FEP or TI are probably the best available approaches.

### 5.3 Binding and Unbinding Kinetics

In the previous sections, we discussed recent computational studies of the thermodynamics of protein–ligand binding, focusing on the determination of absolute and relative binding free energies. This emphasis reflects the medicinal chemistry community’s historical interest in the thermodynamic aspects of the drug-target binding. In recent years, however, the community has developed a growing awareness of the role of kinetics, particularly unbinding kinetics,^11^ in determining the efficacy of a drug in a clinical trial.^261−263^ Since this field is relatively recent, there is less literature relative to thermodynamic studies. Similarly to the literature on thermodynamics, the current literature can be divided into two groups (other classifications are possible^264^). First, there are studies that target the absolute estimation of kinetics parameters. Then, there are works that are more oriented to drug discovery, where scientists seek linear or ranking correlations between experiments and simulations. These usually involve an entire series of systems, rather than being restricted to a single drug-target choice. These two categories have different aims. On the one hand, obtaining the absolute kinetic constant is difficult and often time-consuming. On the other hand, ranking and finding correlations is usually faster and more likely to be used by industry to prioritize drugs. The first approach is closer to basic research, while the second approach is closer to engineering, in that one is seeking a practical solution to the need of industry to prioritize drugs based on the *k*_off_.

#### 5.3.1 Kinetic Properties from Unbiased MD

The first group of binding-unbinding kinetics studies that we review is based on unbiased MD. Unbiased MD is expensive but has a few important advantages. First, it does not require previous knowledge of binding poses, and it provides an impartial coverage of hortosteric and allosteric binding sides. Second, in addition to kinetic coefficients, it provides a detailed description of reaction pathways and intermediate stages. The challenge is to extract as much meaningful information as possible from the overwhelming amount of data provided by MD trajectories.

**Figure 15:**
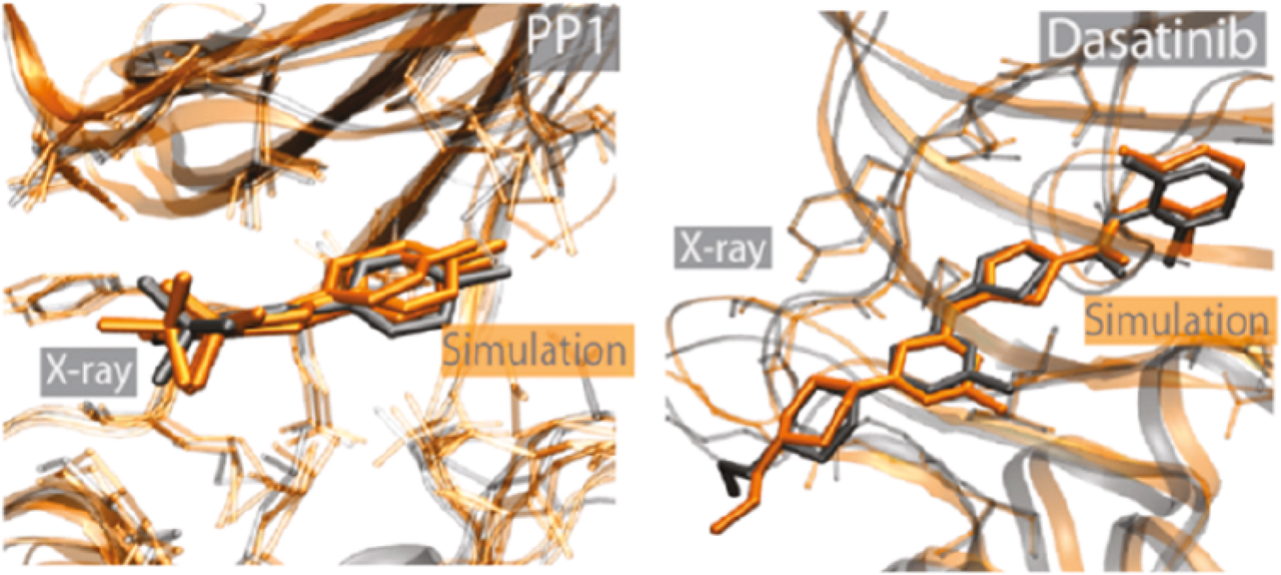
PP1 and Dasatinib binding poses obtained from μs-long unbiased MD simulations.^17^ Reproduced with permission from ref (17). Copyright 2011 American Chemical Society.

A work from the D. E. Shaw group^17^ is the first recent milestone in MD-based kinetics in plain MD for protein–ligand binding. The authors used the Anton machine^59^ to observe, for the first time, the cancer drug dasatinib or the kinase inhibitor PP1 spontaneously bind to the Src kinase. The simulation is unbiased, and the observation of a few binding events allows an estimation of the corresponding kinetic rates (see Figure 15). Moreover, the majority of successful binding events reproduce the pose observed by X-ray crystallography, and the progression of the ligand toward the binding pocket supports the funnel picture of protein binding.

In particular, the authors observe that Dasatinib binds after 2.3 μs in one of four independent simulations (for a total of 35 μs), while the other simulations end with Dasatinib close to other regions of Src kinase. In contrast, PP1 was observed to bind after 15.1, 1.9, 0.6 μs (out of seven independent simulations, for a total of 115 μs). For Dasatinib, the authors obtained an on-rate coefficient of 4.3 s^–1^μ M^–1^, in striking agreement with the experimentally measured value of 4.3 s^–1^μM^–1^ (although with an indefinite error bar, since a single successful binding was observed). The authors also remark on the important role of water molecules, consistently finding crystallographic water molecule positions. Moreover, for PP1 binding, a water shell surrounding the ligand generated an entrance kinetic barrier. The authors also noted that the protein ligand binding is a complex and sometimes activated process, with water molecules playing a major role. Also very interestingly, it was observed that PP1 is able to dock at alternative binding pockets, demonstrating that long unbiased MD runs can detect allosteric sites that are not apparent in crystallographic structures.

The same group simulated the spontaneous binding of three antagonists and one agonist ligand to the β_1_ and β_2_ adrenergic receptors (AR), running 82 simulations for a total aggregated time of 230 μs,^18^ achieving 21 successful binding events. As for the previous study, they used unbiased MD. The choice of the ligands covers compounds used to treat (as antagonist) hypertension and angina pectoris, as well as (as agonist) bradycardia and heart block. Since β-AR is an integral membrane protein, the simulation represented it embedded into a lipid bilayer. Ligands were placed at least 30 Å from the orthosteric binding pocket, and their release once bound was not observed. Simulation results gave an on-rate coefficient of 31 s^–1^μ M^–1^ for alprenolol and dihydroalprenolol binding to β_2_*AR*, which is very close to the experimental value. In one case, the binding free energy estimated by FEP (−13.4 ± 1.6 kcal/mol) was close to the experimental value (−12.2 kcal/mol). The binding mechanisms for (S)-alprenolol and (S)-dihydroalprenolol (now both named Alprenolol) were found to be very similar. Of 12 simulations that gave a bound pose, 6 matched the crystallographic structure, while the others ended in less energetically favored prebinding poses. In simulations that gave bound poses, alprenolol almost always followed the same binding path, with metastable states also being consistently reproduced. In more detail, the authors identified a major intermediate step where the ligand spent a significant amount of time. They named this area of the GPCR the extracellular vestibule.^18^ With additional simulations, it was shown that the first and highest barrier is not from the vestibule to the binding site but rather from the bulk to the vestibule. This finding is somewhat surprising since the path from the vestibule to the binding site involves deformation on the protein and the squeezing of the ligand through a constriction. Analysis of simulation trajectories suggests that the high barrier from the solvent to the vestibule site is determined by the near-complete desolvation of the ligand and by the partial desolvation of the binding pocket.

**Figure 16:**
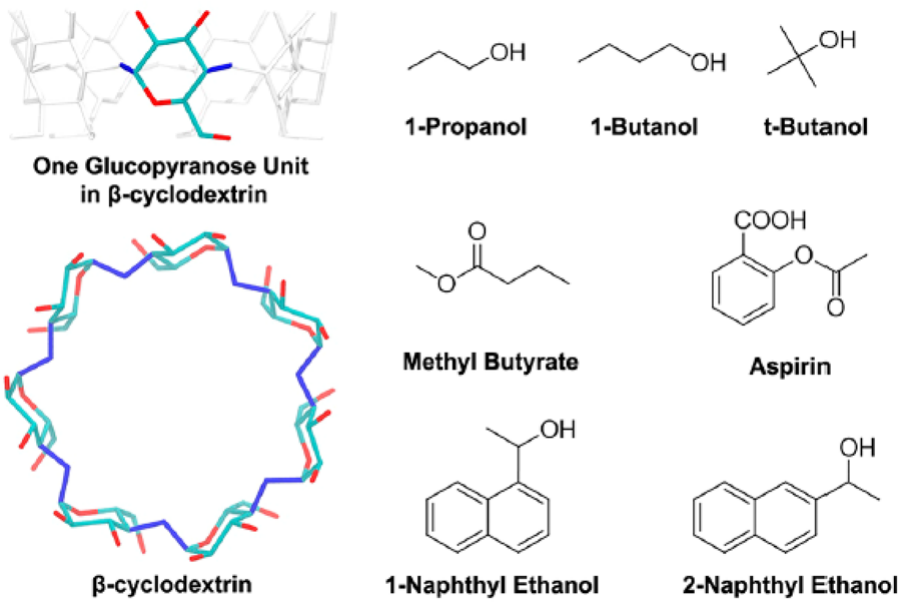
Host (β-cyclodestrin)-guest systems studied in ref (265). Systems of this kind are increasingly used to benchmark free energy computations since their small size allows exhaustive simulations. Reproduced from ref (265). Copyright 2018 American Chemical Society.

The role of water solvation/desolvation of the ligand and of the binding pocket was analyzed again by multi-μs plain MD simulations in ref (265), which discussed the binding and unbinding kinetics of a host–guest system. While these systems are not classical protein–ligand binding complexes, their analysis is of paramount importance as they are simplified systems in which reproducibility of results is increased due to their simplicity. As such, these systems are particularly interesting for discussion and benchmarking. The host system in this case is a β-cyclodextrin (β-CD), which is a model system for a binding cavity, whereas the guest is one of seven small organic molecules, including aspirin (see Figure 16).

In pharmacology, systems of this kind are primarily models. However, as anticipated, CD are nevertheless of practical interest in many fields, such as cosmetics, drug delivery, catalysis, food, and agriculture. Two force fields were used (i.e., GAFF and q4MD) to compare their predictions. The binding enthalpy and entropy of each complex were directly estimated from plain MD either as average potential energy or via configurational integrals. During the simulation of each complex in ∼1700 water molecules, several binding and unbinding events were observed, and kinetic parameters were estimated directly. Then, *k*_on_ was obtained via the inverse of the average unbound time multiplied by the solute concentration, and the *k*_off_ was the inverse of the average bound time. A ligand was considered unbound when the distance with respect to the center of mass of the β-CD was greater than 7.5 Å. Moreover, to account for fluctuations, a bond was not considered broken or formed unless the complex remained dissociated or associated, respectively, for at least 1 ns. To compute the free energy of binding, the authors used and compared two formulas. The first formula was Δ*G*^*o*^ = Δ*H* – *T*Δ*S* (quite unusual for simulations). The second formula was derived from kinetics according to Δ*G*^*o*^ = −*RT* ln(*k*_on_*C*^0^/*k*_off_), where *C*^0^ is the standard concentration (1 M). An important result was that the Δ*G*^*o*^ computed by the two routes and with the two force fields in most cases agreed with each other and with experiments to within 2 kcal/mol. However, the decomposition of Δ*G* into its enthalpy and entropy contributions strongly depended on the force field. To some extent, this reflects the fact that free energy is a variational quantity and is thus more stable and easier to compute than either enthalpy or entropy. Despite the uncertainties, it is apparent that the entropy gain in releasing water molecules absorbed on CD is a major driving force for ligand binding. Interestingly, the estimate of Δ*G* obtained from *k*_on_ and *k*_off_ is more accurate than the estimate obtained from phase space averages. To some extent this is not surprising, since the estimation of thermodynamic properties from kinetics is considerably simpler numerically than the phase space approach. Moreover, the logarithmic dependence of Δ*G* on the kinetic coefficients moderates their variations and might help explain the better performance of the kinetic route. However, it is exceedingly time-consuming in most cases to observe the many binding/unbinding events required for a reliable estimate of *k*_on_ and *k*_off_ by plain MD. Obtaining kinetics from thermodynamics is difficult, and obtaining thermodynamics alone is already challenging. However, whenever feasible, obtaining thermodynamics from kinetics may be convenient as kinetics is directly observable by measuring rates and times.

#### 5.3.2 Unbiased MD and Markov State Models

De Fabritiis’ group reported a comprehensive study of the binding-unbinding kinetics of the benzamidine inhibitor of β-trypsin using unbiased MD, whose results are distilled into a Markov state model.^16^ In an impressive campaign, 495 simulations of the trypsin-benzamidine system were run for 100 ns each. The simulation started with the ligand in the solvent, and spontaneous binding was observed in 187 cases (37% of all simulations), with the system settling into a binding pose whose RMSD distance from the experimental one was less than 2 Å.

Three Markov state models were built from the collected data. Two were mainly used to summarize data, representing the system evolution on a 2D projection, or according to a simple 5-state model. The third MSM was 3D and more quantitative, obtained by covering a 3D space of collective variables with a grid of 18 × 18 × 30 bins. Analysis of eigenvectors and eigenvalues of the transition matrix allowed researchers to identify stable and metastable states and to compute their relative free energy. This information, in turn, provided both *k*_on_ and *k*_off_ coefficients in fair (but not excellent) agreement with experiments. Unbinding was never observed during unbiased MD runs, but the corresponding *k*_off_ rate coefficient is implicitly determined by sum rules and equilibrium relations built into the MSM. No attempt was made to improve the model by selected MD runs, launching swarms of short trajectories probing the transition states among stable and metastable valleys. In addition to this quantitative information, the study provides insight into the binding mechanism. As with the D. E. Shaw group trajectories, the ligand here probed several regions of the surface. This again highlights the complex nature of the binding process, which involves intermediate stations and alternative pockets. Similarly ref (19) used MD simulations 13-μs-long, coupled to machine learning (clustering, graphs), to map the complex binding kinetics of the DADMe-immucillin-H inhibitor (DADMe) to the human purine nucleoside phosphorylase (PNP) again underlying the complexity of the phenomenon.

Proteins are dynamical entities on all length scales, up to whole domain motion, visiting different conformations on the microseconds time scale. This plasticity aspect of proteins and the binding of small ligands affect each other. This is because the spontaneous change of conformation may modify binding pockets or expose new ones, while binding itself may induce the protein transition to a new stability basin (induced fit). A feature that is so similar that it is difficult to distinguish is conformational selection, in which the change of protein conformation occurs before the binding of a ligand. These wide amplitude structural changes are slow, hence they are challenging to investigate by simulating their effect on binding kinetics.

Ref (15) successfully investigated the effect of plasticity on the kinetics of ligand binding. This study again considered the benzamidine and β-trypsin system, using the same method as ref (16). Here, an MSM was built based on an even longer (150 μs) MD simulation. The transition matrix was refined by restarting trajectories connecting undersampled basins. Analysis of eigenvectors identified a variety of metastability basins, representing six apo-Tripsin states, seven bound and four associated conformations. Binding and unbinding was fast, while the structural interconversions were slow. For instance, the six apo conformations were visited by the protein on time scales of tens of microseconds, exposing different binding pockets on their surface. The different conformations found by MD-MSM can be recognized in the crystal structure of the wild and mutated proteins, supporting the validity of the computational picture.

Induced fit and conformational selection are key issues in two further studies using MD/MSM to compute kinetic constants and to characterize the binding mechanism. Ref (91) reports the use of MD with Markov State Modeling to study the single domain protein par-6 PDZ, carrying the peptide recognition module PDZ, whose ability to recognize peptides is allosterically modulated by binding to the Cdc42-GTP protein. In ref (91), 400 ns-long MD, followed by clustering of configurations and analysis of transitions, show that this approach can identify both conformational selection and induced fit in the system. Conformational selection, in particular, plays a significant role in the kinetics of par-6 PDZ binding.

The binding of choline to the choline binding protein (ChoX) was investigated in ref (92). This study used the MD/MSM combination supplemented by analysis of fluxes along each pathway from reactants (choline and ChoX in solution) to product (the choline/ChoX complex in solution), accounting for all intermediate states identified by MSM. It was concluded that conformational selection and induced fit are in fact idealized extreme models of binding, while real cases occur by a superposition of both.

#### 5.3.3 Applications based on the Weighted Ensemble

Recent applications of the weighted ensemble method in biophysics and in pharmacology rely primarily on its WExplore formulation,^94^ which runs multiple trajectories in parallel, and covers the space with a hierarchical and adaptive choice of bins, consisting of Voronoi polyhedra (see section 3.7.2).

WExplore applications relevant for pharmacology are exemplified by a recent study of the unbinding of the TPPU inhibitor from its soluble epoxide hydrolase target,^266^ which is a protein involved in the synthesis of cholesterol. It is therefore of interest for the treatment of hypertension, arteriosclerosis, and a number of other cardiac and circulatory diseases. TPPU can bind at several sites buried within the protein, and its experimental residence time is 11 min, far exceeding the μs–ms range accessible to several other accelerated methods. It is crucial here that WE can provide unbiased results on time scales much longer than those explicitly simulated.

In this work, simulations were started from the binding site and the target state is the fully solvated ligand. To provide data to compute rates, trajectories from the initial to the final state were reinterpreted without further refinement according to a steady state picture. The mean first passage time was computed from the flux from the starting to the final bin, according to the Hill relation (Eq. 18) reported in section 3.7.2. The estimated residence time of 42 s with a standard error of ∼10^2^ s was within 1 to 2 orders of magnitude of the experimental value of 11 min = 660 s. Notably, this result was obtained based on an aggregated time of only 6 μs covered by unbiased MD.

Trajectories were analyzed, revealing a number of features of the unbinding process, including the precise identification of the interactions that most affect the unbinding time. The role of hydrogen bonding, solvation, and desolvation along the unbinding trajectory and the ligand dynamics in a sort of near-unbound state were also analyzed.

WExplore’s ability to identify and rate different transition paths was highlighted by a previous study from the same group studying the unbinding of benzamidine from its trypsin target.^267^ The multitrajectory character of WExplore is a crucial feature here. In this investigation, ns-scale unbiased MD runs were used to generate steady state data. These, in turn, were used to predict an exit time of 180 μs, i.e., within an order of magnitude of the measured value (1700 μs). Clustering of configurations and analysis of trajectories revealed the parallel activity of three exit channels, two of which form through large-amplitude motions of loop structures of trypsin. One of these modes was still unknown, since previous simulation studies had failed to reveal it.

A further relevant study carried out by WExplore concerned the unbinding of three ligands from the model protein FK506,^22^ which is known to bind a large number of drug-like molecules. The low binding affinity of the ligands results in short residence times of the order of nanoseconds, but WExplore also enjoyed an efficiency advantage here with respect to other methods, besides achieving a remarkable agreement with the results of plain MD simulations that were obtained at significantly higher cost. On short time scales, not having to rely on a Markovian assumption is a critical advantage of WExplore. The method’s ability to characterize structural properties of the unbinding pathways was exploited here to investigate for the first (and probably only) time the distribution of the ligand exit points and to determine their spread. The resulting probability distribution was analyzed with a general statistical tool (von Mises-Fischer model).

As pointed out by the papers reviewed here, the efficiency of WExplore is achieved by increasing the probability of visiting high free energy regions of the phase space, such as transition states. The method requires a number of user-defined parameters to run the simulations, such as the definition of bins, the number of trajectories, and the time interval between two resampling steps. Remarkably, these parameters turn out to be rather transferable from one system to another, and the method is one of the most promising choices now available, especially for long time scales.

#### 5.3.4 Applications Based on Metadynamics (MTD)

Switching to bias-based methods, a metadyamics-based approach to kinetics was recently proposed^21,268^ showing that, if the deposition rate of Gaussian-shaped potentials is slow enough, then the metadynamics bias does not significantly affect the transition state, and the kinetics can be recovered quantitatively. This conclusion is based on the assumption that the time to cross the barrier from bound to unbound (or vice versa) is short with respect to the residence time in the starting and final free energy basins, and a statistical test is available to verify a posteriori the validity of this assumption.^269^ This technique was used to study the benzamidine inhibitor of trypsin,^21^ a drug-target model already investigated several times by computational means. Besides its testing value, the interest in this system is justified by the fact that trypsin is a protein that catalyzes the hydrolyzation of amino acid chains, it is relevant in a number of biotechnology processes, and it plays a role in the onset of pancreatitis. Metadynamics with an aggregated MD time of 5 μs activated a statistically significant number of benzamidine-trypsin unbinding events, giving a prediction of *k*_off_ = 9.1 ± 2.5 s^–1^, only in qualitative agreement with experiments (*k*_off_ = 600 ± 300 s^–1^). Comparison with the results of a Markov state model built from the same simulation data showed fair agreement, mutually supporting the validity of both approaches. In this respect, a few considerations are in order. First, the detailed knowledge of the transition matrix of MSM is not needed if the basic kinetic coefficients *k*_on_, *k*_off_ are the only objective. However, the analysis of the eigenvalues and eigenvectors of the transition matrix provides a more comprehensive view of the process. Moreover, building the MSM from the trajectories generated during the metadynamics runs does not require further large computations. However, in this case, the view provided by the MSM stage could be limited by the sampling restrictions introduced to enhance the efficiency of both metadynamics and its funnel variant.

A recent refinement of the method introduces an adaptive rate of Gaussian bias deposition,^270^ motivated by the observation that a low deposition rate along the whole process may result in long simulation times. The adaptive approach uses a fast deposition rate at the beginning of simulations, when the system is well within the starting basis. The deposition rate is decreased later, when the system approaches the transition state, whose unbiased sampling is crucial for an accurate estimate of the true transition rate coefficients. As noted in ref (271), the main limitation of this promising approach is the assumption that the unbinding process is just a two-basin problem. A second drawback, common to other methods, is the need to define the collective variables.

A recent application of metadynamics in the drug discovery context is reported in ref (272), where the unbinding process of Dasatinib from c-Src kinase was investigated (see also section 5.3.1). In this paper, 12 independent metadynamics runs were conducted, each between 150 and 750 ns long. Each run led to the unbinding of the ligand, which was not observed in the 35 μs unbiased MD of ref (17). The estimated residence time of 21 s is in excellent agreement with available experimental values. The collective variables used were: (i) the distance of the ligand from the binding site and (ii) the solvation state of the binding pocket. Although the method of infrequent metadynamics was conceived for kinetics, an approximate but comprehensive picture of the free energy surface could also be obtained, revealing six (meta)stable basins, including the final unbound state. Each of these basins was thoroughly described from the structural viewpoint. Analysis of trajectories, for instance, highlighted that a salt bridge and a water molecule temporarily residing in the binding pocket played a complementary role in triggering the unbinding process.

Another work^273^ investigated the degradation of the persistent anthropogenic pollutant 1,2,3-trichloropropane (TCP) due to haloalkane dehalogenase (DhaA) or to a mutated version (DhaA31). The computational analysis focused on the unbinding kinetics of the enzymatic product 2,3-dichloropropan-1-ol (DCP) because it has been identified experimentally as a rate-limiting step, particularly for DhaA31 which exhibits a much improved catalytic rate with respect to the wild type. The rationale of the study was that, by boosting the unbinding process, it would be possible to design further modified DhaA31 variants with fast enzymatic rates that avoided the bottleneck of the unbinding of DCP, thus resulting in an even faster degradation of TCP. Despite its biotechnology and biocatalysis flavor, this paper is interesting for pharmacology, in that the ligand is left unchanged and the modification is on the protein side. This paper combined several computational approaches: adaptive sampling and high-throughput MD,^274^ infrequent metadynamics and Markov state models for kinetics, and funnel metadynamics for the free energy determination. To use metadynamics, a path collective variable was introduced according to ref (198). Twenty five simulations were run to refine the description of kinetics and, following infrequent metadynamics theory, unbinding times were debiased and an estimation of the k_off_ was obtained. In line with experimental knowledge, the release from DhaA was much faster than in the DhaA31 case. Moreover, hotspots suitable for DhaA31 modifications were found, suggesting ways to improve both the unbinding kinetics and the overall rate of TCP degradation. Somewhat surprisingly, a much cheaper docking protocol (CaverDock^275^) gave indications about the DCP undocking from DhaA31 that were close to those of MTD. Here too, the results of metadynamics were compared to those from a Markov state model. In contrast to ref (21), the absolute *k*_off_ value obtained here from metadynamics differed by 2 orders of magnitude from that obtained via MSM. The relative ranking between DhaA and DhaA31, however, was retained albeit with different ratios. The disagreement between the nominally equivalent protocols implementing metadynamics and MSM points to a residual development deficit, meaning that the results still depend on user choices concerning the metrics (Markov State Models) and collective variables (metadynamics).

Notably, the ranking was correct in both cases, highlighting how relative estimations tend to be easier and more reliable. It is thus obvious that, in a real-world drug discovery campaign, there is no need to run very long simulations for absolute values, when fast and relative estimators are more effective.

#### 5.3.5 Studies Involving Diffusion

Another key aspect that emerges in the literature on kinetics predictions is the importance of taking into account not only the late stage of the binding process, when the encounter complex is already established, but also the diffusive stage, when the ligand approaches its target through a random walk. In fully atomistic unbiased MD, the diffusive stage might take longer than the actual binding stage, especially if the activation barrier for entering the binding site is low. The different character and duration of these stages requires a multiscale approach.

**Figure 17:**
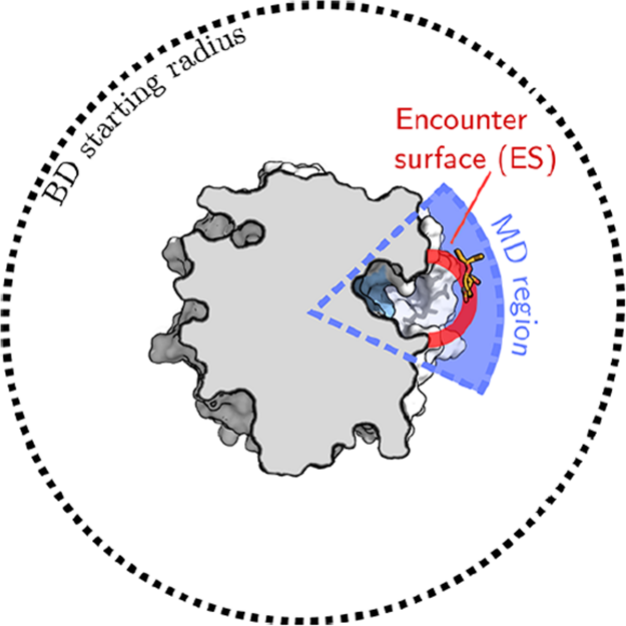
Example of space decomposition in regions explored by Brownian dynamics (BD) and MD. The BD region is far from the protein, where the ligand diffuses nearly freely, subject to only long-range electrostatic interactions. Dispersion forces and steric interactions need to be accounted for in the explicit MD region. Reproduced from ref (276). Copyright 2017 American Chemical Society.

In ref (276), the problem of predicting *k*_on_ is split into two steps. The first step applies Brownian dynamics (BD) to simulate the ligand’s approach to the binding site from 60 Å away. The second step considers the actual docking and is simulated by plain MD. BD allows the user to simulate longer time scales with respect to MD by approximating electrostatic interactions and using an implicit solvent, represented simply by a stochastic force. The ligand motion is restricted to roto-translations, neglecting molecular flexibility. The crucial matching of the BD trajectory and the plain MD simulation with explicit solvent occurs at a predefined encounter surface located 12 Å from the geometric center of the binding site (see Figure 17).

**Figure 18:**
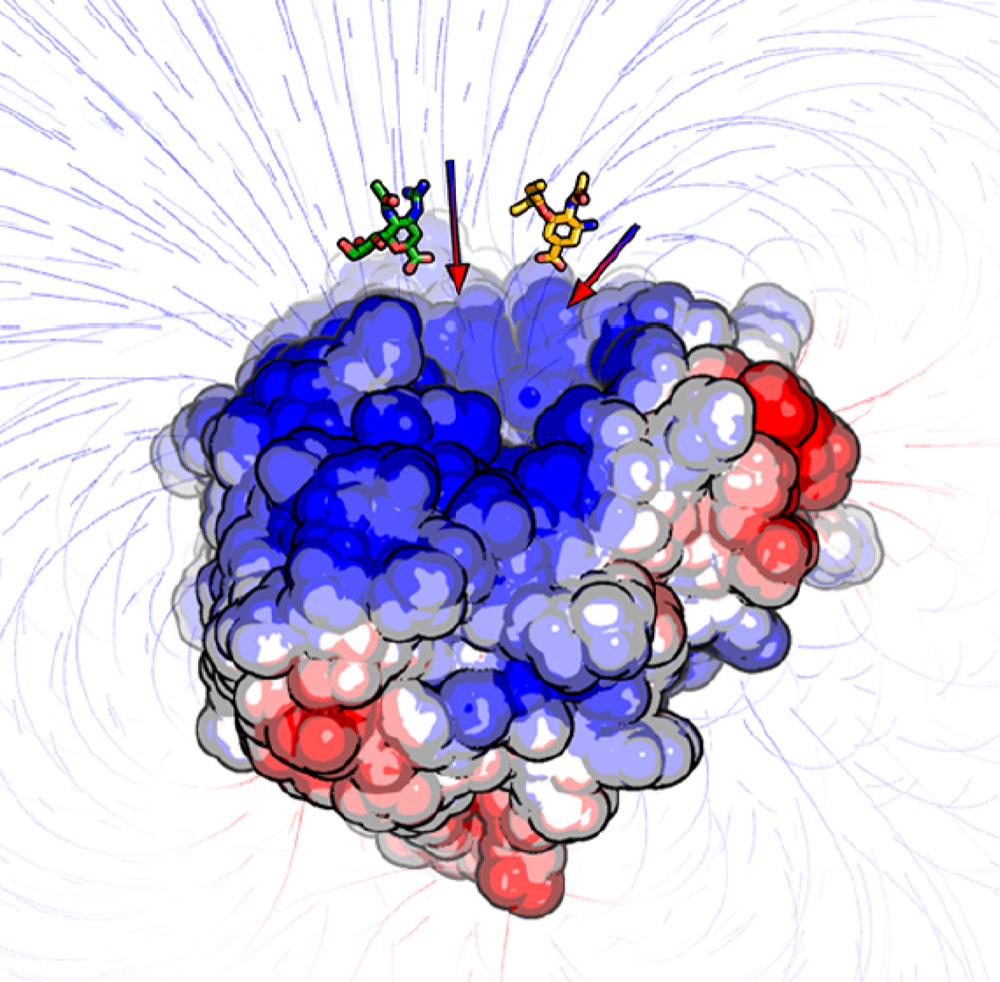
Neuraminidase electrostatics field lines guide the binding of oseltamivir (orange) and zanamivir (green). This is a key phenomenon that rules the *k*_*on*_ in protein–ligand binding. Reproduced from ref (276). Copyright 2017 American Chemical Society.

The advantage of this approach is that the long-range diffusion part of the ligand-target approach can be properly taken into account when computing absolute *k*_on_ values, which was the main aim of that work. The method was applied to two inhibitors (oseltamivir and zenamivir) of the influenza H1N1 neuraminidase (see Figure 18).

The computational results, based on a 50 μs trajectory for oseltamivir, and 37 μs trajectory for zanamivir, agree fairly well with experimental data on *k*_on_.

One crucial question raised by the paper is how to decide when a ligand changes from unbound to bound, as required in the computation of kinetic parameters. Indeed, the simplest definition of a bound state is when the ligand acquires the crystal pose to within a preassigned root-mean-square distance. This, however, requires the detailed experimental knowledge of the complex structure in the vicinity of the binding pocket. Moreover, when simulating the binding process, the ligand often enters the binding site but does not acquire the experimental bound pose, hovering on an alternative site, before eventually moving to the final binding pose. Since this final step might not occur during the simulation time, one is left to decide whether the final result is due to inaccuracies of the model or to incomplete sampling. Moreover, it could also be that the binding pose found by simulation is the most realistic one in solution, while it differs from the most stable one in the structure of the complex determined by X-ray diffraction on the crystal phase at low hydration.

To overcome this problem, a new definition of a bound state has been introduced,^276^ based on the residence time of the ligand. More precisely, a trajectory is considered bound when the ligand resides for at least 2 μs in the explicit MD region, i.e., on the bound side of the encounter surface. This point of view is interesting since it requires only a very approximate knowledge of the actual binding pose. More importantly, it implicitly assumes that even alternate poses and encounter complexes are already inhibiting. While we agree that alternate poses may be inhibiting and may thus pragmatically match the bound definition, it is questionable to assert that a ligand is bound only because it sits within a sphere around the binding site, especially when it is still at least partially solvated.

Another limitation of the overall approach, as the authors point out, is that the advantage of the multiscale method is significant only when the activation barrier for binding is low. In the opposite case, the time required for moving from the encounter surface to the bound state far exceeds the duration of the diffusive stage, and the efficiency gain in using the multiscale approach cannot be a major one. In all cases, the explicit and unbiased MD for the final binding stage may reveal important details on the binding mechanism. In ref (276) for instance, the explicit MD stage highlighted the role of a salt bridge in the binding process.

In a related paper,^277^ Brownian dynamics and MD were again combined. This time, they were used to parametrize a milestoning model.^278^ The test system was again the trypsin-benzamidine complex. Milestones, in this case, are represented by concentric spherical surfaces centered on the binding site. BD and MD together provide an efficient way to estimate the transition rates across these surfaces, thus easing the required parametrization of the milestones model. On the basis of only 19 μs of detailed MD simulation, the method determined *k*_off_, *k*_on_ and consequently the binding energy via the Arrhenius relation. This performance is about 1 order of magnitude faster than that of Markov state models of comparable resolution and accuracy. In our opinion, milestoning is by construction more efficient than Markov state models, so this result is not surprising.

This work is also relevant because it introduces a collection of scripts dubbed SEEKR,^279^ whose aim is to automate the preparation and running of simulations and to ease the analysis of trajectories. The SEEKR tool is open-source and distributed via github. This tool’s availability greatly enhances the method’s applicability to other complexes and increases the reproducibility of results. Interestingly, a few years before this paper’s publication, a forerunner study^280^ had already promoted BD as a suitable tool for studying protein–ligand binding kinetics, although this early paper targeted encounter complexes only.

Overall, the SEEKR approach and WExplore^267^ are very powerful and should be seriously considered when absolute kinetics computations are sought.

Another important aspect in kinetics studies is the role of solvation and desolvation of both the ligand and the binding pocket, which is highlighted and emphasized by many studies. The role of solvation and desolvation is often reflected in free energy barriers that affect *k*_on_, *k*_off_, which explains why explicit solvent simulations are strictly required in many cases.

In ref (281), the desolvation (drying) of the binding pocket before binding is explicitly addressed in a study of Dasatinib binding to the Src kinase, i.e., the same system investigated in ref (17). In this study, the authors ran umbrella sampling with reaction coordinates consisting of (i) the center of mass distance between ligand and binding pocket and (ii) a smooth function of the water occupancy of the pocket itself. Simulations were based on the OPLSA force field^28^ and the TIP4P water model.^50^ As a first step, steered MD for unbinding was used to obtain an initial trajectory for subsequent umbrella sampling. This preliminary simulation was started from the experimental crystal structure and used the single coordinate corresponding to the center of mass distance. Overall, the 2D umbrella sampling domain had a total of 60 windows, which allowed a sufficient sampling at 2 ns per window to let WHAM correctly reconstruct the free energy surface. The WaterMap^282^ analysis tool was also used to quickly estimate the desolvation barrier due to the presence of water molecules. The potential of mean force (PMF) obtained through WHAM clearly showed an entrance barrier for the ligand with a height of about 3.7 kcal/mol. Attributing this barrier to the pocket desolvation free energy required some checks. First, the authors computed the PMF using MM-GBSA. This showed that the entrance barrier was lost when moving from the explicit to the continuous model, suggesting that the barrier was due to desolvation. As a semiquantitative test, the authors also used WaterMap to estimate the desolvation energy, obtaining good agreement with umbrella sampling calculations. As a last verification, the PMF was recomputed by applying a restraint to the binding pocket. The similarity of the free energy barrier computed with and without the restraint excluded the possibility that the entrance barrier was due to the pocket rearrangement. Overall, this paper quantified and underlined the important role of water during the binding process, with desolvation of the binding pocket being a fundamental prerequisite for completion of the binding process.

In addition to the real molecular complexes considered in this section, it can also be interesting to study model systems to obtain an in-depth understanding of the chemical-physics aspects of binding, albeit in an idealized configuration. One such example is ref (283), which considers the rate of hydrophobic association using a spherical concave surface recess and a spherical model ligand, both hydrophobic, taking place in explicit TIP4P^50^ water solvent. Starting from the results of previous studies, pointing out that solvent fluctuations create a barrier to hydrophobic association, the paper analyzes how geometric and energy parameters affect the association rate, measured by *k*_on_. In particular, a more water-exposed pocket (a larger entrance radius) is shown to create a higher entrance barrier for binding, whereas a deeper pocket favors hydrophobic interactions. As expected, the association rate is greatly increased by increasing the pocket’s hydrophobicity, measured by the inverse average number of water molecules it contains. This effect was quantified in relation to water fluctuations. Large fluctuations, in particular, point to dewetting and stronger hydrophobicity. It was also possible to identify a critical threshold value of the geometric pocket depth, for which the water fluctuations significantly increased, thus allowing a simpler replacement of water molecules by the ligand. It was also noted that, if water fluctuations vanished, the binding time could diverge to infinity. This conclusion might seem paradoxical, but it becomes more acceptable when considering that no (or little) fluctuation corresponds to the solid phase limit, in which the association process is infinitely slow.

#### 5.3.6 Ranking Ligands According to *k*_off_

The papers discussed above all aimed to compute absolute kinetic rates. Other approaches are suitable for relative ranking or correlative estimates^24,25^ in order to analyze entire series of ligands. Such methods can be particularly useful for comparing *k*_off_ rates that cannot yet be computed by plain MD and that are also challenging for accelerated sampling methods.

A recent attempt in this direction^25^ was based on scaled MD, uniformly reducing the whole potential energy by a constant scale factor λ and preventing the protein unfolding by imposing restraints on the protein backbone except the binding site. The protocol was tested on the chaperone heat-shock protein 90 (HSP90), the 78 kDa glucose-regulated protein (Grp78), and the adenosine A2A receptor (A2A GPCR), all of great pharmaceutical interest, combined with three corresponding sequences of ligands, each derived from a different scaffold. This simple scaled MD trick with the scaling factor λ = 0.4, equivalent to increasing the temperature by a factor λ^–1^ = 2.5, caused the unbinding of all ligands during times from nanoseconds to tens of nanoseconds. Each unbinding simulation was repeated several times, providing an average over the initial conditions. The ratio of the unbinding times for a series of congeneric compounds were scaled to a common baseline by an Arrhenius-like relation.

The unbinding times computed in this way do not reliably estimate the absolute unbinding time (or, equivalently, *k*_off_). However, it is intuitively acceptable that they could retain the correct ranking of compounds when the same scaling λ is applied. The correlation with the experimental ranking would improve by increasing λ. The results of this study confirmed the expectation, displaying consistent correlations (from 0.85 to 0.95) with the experimental results. The internal consistency of the data and their confidence limit is assessed by a statistical bootstrap analysis. The method, moreover, is trivially parallel, and does not require the prior definition of a reaction coordinate. The choice of the binding site that is left free from restraints is the only aspect that might prevent a completely automatic application of the method.

The same group challenged the scaled MD protocol against seven noncongeneric glucokinase activators^69^ that are being considered as targets for treating type 2 diabetes mellitus. The authors investigated the dependence of the ranking on the choice of the scaling factor λ, showing that λ = 0.5 (already used in ref (25)) was adequate in all the analyzed cases, but a lower λ = 0.4, giving faster unbinding, was sufficient to rank congeneric compounds. It was also found that the unbinding paths could be a source of interesting albeit approximate information from this kind of simulation. Hence, the results of this study further validated scaled MD as a tool for ranking compounds on the basis of their *k*_off_ value.^284,285^

Callegari and colleagues^271^ devised a conceptually simple method based on metadynamics to rank ligands according to their *k*_off_. Like other methods, the proposed approach requires experimental structures for the complex to start the unbinding simulations. In this study, the method was used to rank 10 arylpyrazole inhibitors of the cyclin-dependent kinase 8 (CDK8) protein. Metadynamics was applied using a complex choice of seven collective variables. Variations in the deposition rate of the Gaussian bias of MTD was used to identify the unbinding event and thus define an unbinding time *t*_MTD_. Needless to say, this time is greatly affected by the MTD bias. Although the real residence time could be recovered by elaborating the simulation data, *t*_MTD_ itself was deemed sufficient to rank the ten ligands. The reported results were able to discriminate between ligands with short and long residence times. The authors claim that their approach’s positive result was because it included the residence time estimate of all stages of unbinding, covering not only the crossing of the highest activated state but also the intermediate steps.

**Figure 19:**
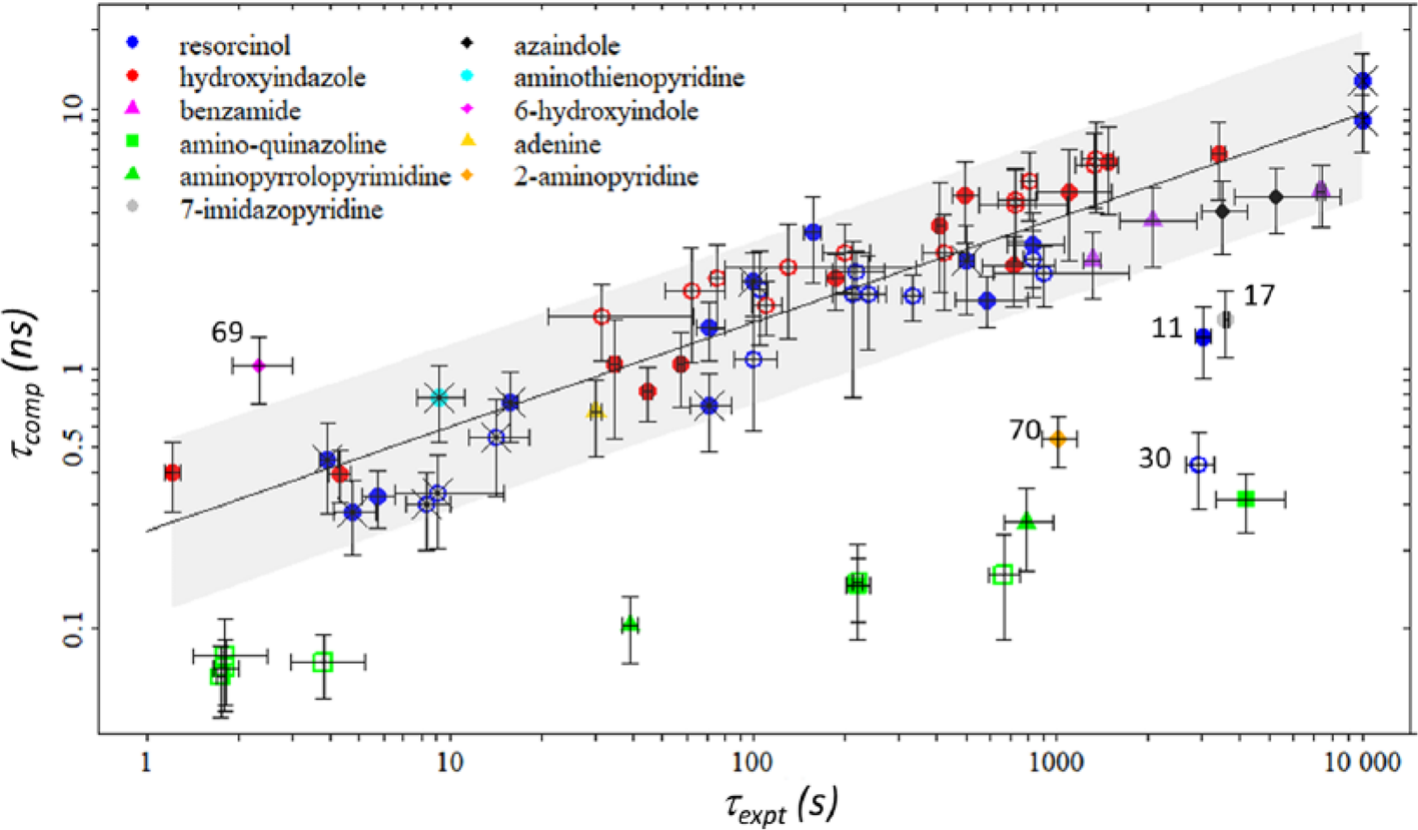
Correlation of residence times for a series of ligands to the N-terminal domain of HSP90α experimentally measured and estimated by τRAMD. Reproduced from ref (24). Copyright 2018 American Chemical Society.

In ref (24), the authors proposed the τRAMD method, consisting of an MD steered by a force whose direction is randomized whenever the ligand displacement over a given time is less than a threshold. Once again, the protocol is initialized (in most cases) by the experimental structure of the crystallized complex, it entails that several simulations started from different velocity distributions, and the size of the random force (14 kcal mol^–1^ Å ^–1^ in this study) is gauged in such a way as to cause unbinding over the time scale of one to tens of nanoseconds. The protocol was used to rank 70 drug-like inhibitors binding to the N-terminal domain of the HSP90α protein, already investigated in ref (25). This protein is involved in the folding of proteins responsible for cell growth and it is therefore a target for cancer treatment. A total of 40–200 trajectories were simulated for each compound, and the quality of the statistical distribution of times estimated for each compound was assessed by the Kolmogorov–Smirnov test. Prior to the computational study, compounds were synthesized, cocrystallized, and the SPR data collected, obtaining residence times that spanned a few orders of magnitude. A comparison of the logarithm of computed and measured residence times showed a good linear correlation, although, by necessity and by design, the computational values were much lower than the experimental ones. Assuming that the linear correlation can be used to extrapolate the τRAMD values to the unbiased limit, the average deviation of computed and measured residence times τ was 2.3τ for all compounds, which was reduced to 2τ if only congeneric compounds were considered (see Figure 19). Additionally, the unbinding trajectories contained useful mechanistic insights. The large amount of data given by this study provides convincing evidence that the method can be useful in the computational ranking of large families of compounds according to *k*_off_.

Another study from the same group^286^ shows how the COMBINE methodology can be used for *k*_off_ prediction. The proposed approach exploits the computed interaction energy terms between the ligand and the protein. Then, partial least-squares (PLS) is used to train a linear predictor of *k*_off_ values, representing a quantitative structure-kinetic relationship. This straightforward protocol was applied to HSP90 and HIV-1 protease. For HSP90, 207 Coulombic terms and 207 Lennard-Jones terms were used, later reduced by filtering to just 12 + 30 as these were the only terms exhibiting statistically significant differences in the ligand set. For HIV-1 protease, 33 compounds were selected and, upon filtering, 17 + 17 energy terms were selected for PLS analysis. Good correlations were obtained for both systems, showing that machine learning methods (PLS being a simple example) can lead to good accuracy predictions when trained with clean data. The limit of this method and of machine learning methods in general is that they require a good and extensive data set for training. This is not required for physics-based methods. However, machine learning methods are vastly more efficient than physics-based methods. In practice, machine learning methods are preferred when sufficient experimental data are available, whereas physics-based approaches are needed when experimental data are not available.

Along similar lines, a previous work proposed a high-throughput data-driven approach for binding kinetics predictions.^287^ In this study, energy and conformational dynamics properties were integrated and fed to a multitask random forest (named multitarget in the paper). The structural dynamics properties were based on the normal-mode analysis (NMA) of a coarse-grained model. Energy properties were represented by the pairwise decomposition of interresidue interaction energies computed at the atomistic level by the CHARMM27 force field. The method was applied to 39 inhibitors of the HIV-1 protease. The computations relied on experimental data to initialize the structure and, when no cocrystal structure was available, ligands were docked into the receptor. The method was trained on experimental *k*_on_ and *k*_off_ values available for the 39 inhibitors, while method was validated using data on mutations of the wild-type protein that are responsible for drug resistance. The prediction task was applied both to *k*_on_ and *k*_off_. Regression was avoided, and ligands were split into four classes, identified by a combination of ranges of *k*_off_ and *k*_on_. This changes the problem from regression to classification, thus significantly simplifying the task, since classification is much simpler than regression and provides a coarse-grained picture. Encouragingly, residues identified as crucial by NMA were also found to be important in molecular dynamics simulations. Moreover, electrostatics energy components were found to be more important in predicting kinetics than full potential or van der Waals energy terms. The results of the NMA analysis are intriguing. They suggest that normal modes of the coarse-grained model already contain predictive kinetic fingerprints, since they drive the estimation of *k*_on_ and *k*_off_ coefficients. This is interesting and potentially useful, but the generality of the results needs further validation. The surprising aspect is that the information entering the NMA is local, focusing on the bound pose of the complex, while the target kinetic coefficients are more global properties, affected by intermediate states and by the whole unbinding path.

A recent work also addressed the problem of ranking ligands based on residence time.^288^ This approach uses the adiabatic bias molecular dynamics^63^ combined with an electrostatics collective variable to promote the unbinding event.^203^ The rationale of this choice is to obtain unbinding events in short times while still trying not to perturb the system in a vigorous way: this is why the adiabatic bias protocol was chosen. The unbinding events are time-averaged and a final ranking based on the timing is given. This approach was successfully applied retrospectively to the glucokinase and prospectively to GSK3β. For GSK3β, the nonnegligible variance of SPR experimental results meant that the comparison between experiments and computations was not trivial.^288^ Finally, the quality of the obtained unbinding paths made them suitable for further free energy computations using path-based methods.

To summarize this section, kinetics is at the forefront of recent computational methods in drug design. Absolute kinetics estimation methods are already powerful and have evolved very quickly in recent years. Some of these approaches have large computational costs, whereas others seem more affordable. Absolute estimations, particularly of *k*_off_, are challenging and not yet particularly accurate, but the correct order of magnitude can often be estimated. In contrast, simpler and powerful relative kinetics methods promise useful ranking capabilities at a more acceptable price. Methods of this type, such as scaled MD and τRAMD, have already been used in drug discovery pipelines and have proven to be reliable and much faster than the absolute approaches, which are expected to become more usable in the long term.

## 6 Recent Machine Learning Trends

Thanks to growing computational power, physics-based simulation is becoming a more viable solution to computational drug discovery, whereas more approximate methods like docking have dominated until now. In this scenario, machine learning (ML) is playing an increasingly prominent role. Machine learning has been used for several years for protein–ligand binding studies in the form of QSAR^289^ or clustering/projection analysis of trajectories.^290,291^ Today, deep learning is widely available. The basic concepts of deep learning are not new and embody the paradigm of learning from data. Well-designed computer libraries and GPUs have now made deep learning computationally feasible.

A new wave of neural algorithms is being used to study protein–ligand binding or even to design ligands, with examples including generative adversarial networks^292^ and variational autoencoders.^293^ Without seeking to cover the whole field, we briefly discuss the role of recent machine learning solutions in protein–ligand binding. We give some pointers to ML topics such as applications of deep learning to predict the affinity of protein–ligand binding and a simplified sampling of the Boltzmann distribution.

### 6.1 Deep Learning for Affinity Prediction

**Figure 20:**
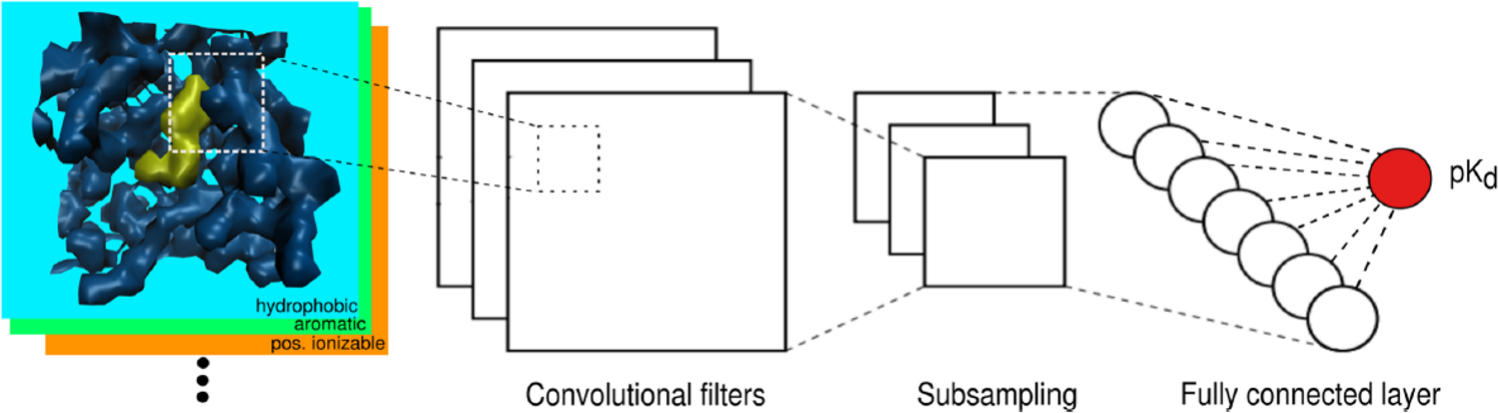
Neural scheme adopted in Kdeep. Reproduced from ref (295). Copyright 2018 American Chemical Society.

AtomNet is the first application of modern neural networks [namely deep and convolutional (CNN)] to the prediction of bioactivity.^294^ The authors built a 3D grid of properties, unfolded the 3D grid into a 1D vector, and applied a CNN to the resulting grids. They applied this technique to the DUDE and ChEMBL-20 PMD data set with good results. Interestingly, the network can actually learn the chemistry from basic grids during the training process. A closely related approach, *K*_DEEP_,^295^ was later proposed. This approach uses 3D convolutional neural networks (see Figure 20).

The main difference between this approach and AtomNet is that the 3D structure of the input grid is maintained. Additionally, the input grids embed more physical information than in AtomNet. The results are competitive with other scoring methods but are significantly less accurate than FEP methods.^295^ These two examples show that deep learning methods (e.g., convolutional neural networks) have been used to predict activity with interesting results. Here, however, the tests are still missing a big data aspect. Indeed, these methods were tested on relatively small data sets, for which the activity data are known. So despite their promise, these methods have not been coupled to a real big data scenario. It is not trivial to obtain big data regarding the activity of druglike molecules. This context is very different from images, which is a classical application of convolutional neural networks. Building a very large data set of labeled pictures is a feasible task.^296^ However, it is very difficult to build a similarly large data set of activity values for druglike molecules for a drug discovery campaign. For this reason, we argue that the full potential of deep models has not yet been exploited in this field.

### 6.2 Collective Variables and Learning the Boltzmann Distribution

Deep learning can also be unsupervised. This means that the value (e.g., activity) to be predicted is not defined a priori. Various studies^297−300^ have leveraged autoencoders, with variants that can automatically learn the important collective variables of the process at hand.

These results are promising because they address one of the most compelling problems in free energy computations, namely, how to identify the slow degrees of freedom that rule the phenomenon under investigation. Reconnaissance metadynamics was an ante litteram method in this regard, although not based on deep learning.^301^ In spirit, the work in ref (300) is quite similar to reconnaissance metadynamics, although metadynamics is not used. If these methods succeed in automatically finding the correct set of collective variables, this would be an important step toward automating the computation of free energy using collective variables, making these approaches more user-friendly, more systematically deployable, and less user-dependent.

A recent contribution, not immediately applied to protein ligand binding, but of undeniable relevance is from Noé and colleagues.^302^ In this paper, an architecture analogous to a variational autoencoder was developed to sample the Boltzmann distribution in an agile way and may potentially overcome the rare events problem. In a variational autoencoder, one defines an input space (the original coordinates) and a latent space, a low-dimensional space in which typically a Gaussian distribution maps the original distribution. This is an inherently generative model, that is, one can generate a new sample in the latent space and correspondingly it can be decoded into the original coordinates. Such an architecture is used in ref (302) to map the Boltzmann distribution in a latent and much simpler space. Learning is possible because trainable neural networks rule the mapping between the target distribution and the latent one by encoding and decoding the samples. The crucial difference between a classical machine learning task and this proposal is that, in the machine learning task, the samples’ generating distribution is unknown. Here, the generating distribution is known and available, namely the Boltzmann distribution. Thus, Boltzmann generators can be trained not only by samples but also by the direct knowledge of the potential energy function. Such a network can be trained to mimic the distribution of complex systems such as a protein in an implicit solvent. It can deliver quite naturally and efficiently free energy estimates at various temperatures. This is because the temperature is directly encoded in the variance of the latent distribution. Although the practical applicability to the ligand binding problem is not yet clear, this approach is elegant in that it links the best of both worlds, creating a kind of gray box modeling as it combines statistical mechanics (white box modeling) with deep learning (black box modeling). We expect more of these connections between machine learning and statistical mechanics^303^ in the future because, in the end, they both deal with distribution sampling and mapping problems.

## 7 Practical Guidelines

As we have discussed in the previous sections, the available methods each has specific virtues and potential pitfalls. A requirement common to all the methods discussed is that one begins from solid structural data. High-resolution crystal and cocrystal structures are an essential requirement for obtaining robust results. Without solid structural data, the systematic application of methods is mostly doomed to failure.

Unfortunately, the user’s method knowledge and the system dependency still plays a major role in successfully applying the methods in several cases. The potential of a method to be engineered in a software, and consequently its automation level, is thus a significant indicator of a method’s maturity. If a method requires many human choices that can significantly change the final outcome then it is simply not mature enough to be used prospectively and quantitatively in real-world scenarios. Commercial considerations alone cannot explain why some methods are not implemented in commercial software or, if implemented, why they are not used by computational drug discoverers, particularly those in industry. Moving from the theory to usable software requires that the method delivers actionable knowledge for drug discovery and that the number of free parameters be reduced. From a user perspective, it is therefore important to differentiate between methods that do and do not require a collective variable. FEP and scaled MD, for example, do not require prior knowledge of a reaction coordinate. This makes them widely usable, although still challenging in complex scenarios such as protein–ligand interactions where the phase space is large and intricate. Of course, in real-world drug discovery projects, there is often significant prior knowledge of the receptor and the ligand series. This makes it possible to operatively define reaction coordinates. But methods that require prior knowledge of a reaction coordinate will likely not become mainstream in the drug discovery community until a higher degree of automation is achieved in defining the collective variables in such a way as prospective applications are feasible.

As a general guideline, we therefore suggest that beginners first familiarize themselves with methods that do not require collective variables. Methods that do require collective variables can be more powerful than relative free energy (FEP) or approximate methods such as MM-GBSA/PBSA, yet they require more effort to be applied and understood so should not be the first methods that one learns in this field.

Despite this didactical premise, we will now offer some rules of thumb for choosing a specific method for a specific problem. The first consideration must be the amount of computational time that one can afford and the level of a priori knowledge of the system. Evidently, if one has both significant computational power (and time) and significant a priori knowledge of the system, then one should immediately consider methods for estimating absolute quantities, such as umbrella sampling, metadynamics, and Markov State Models. In time-constrained situations, more approximate methods such as FEP and scaled MD are advisible. We would suggest using scoring functions or MM-GBSA/PBSA only when time/resources are very limited or when dealing with virtual screening and docking where several compounds are to be evaluated simultaneously.

We offer the following additional guidelines and caveats concerning the methods and scenarios discussed in this review. When an explorative search of the phase space is required and one has a certain knowledge of the phenomena of interest, metadynamics is probably the best solution. Metadynamics automatically and quickly moves in phase space once a collective variable is defined, and it is not difficult to define walls to restrain the exploration to an area of interest. By playing with the hill size, it is also possible to intuitively tune the method’s accuracy and speed. Metadynamics is probably the best tool for exploration when a reaction coordinate can be guessed a priori. The delivery of a free energy estimate can be a significant bonus in many cases, but this requires a careful check of convergence to declare a reliable free energy result. For instance, one reasonable criterion is to check that the free energy difference or the obtained profile is stable for sufficient time and to run several replicas of the metadynamics simulations.

Markov State Models and the weighted ensemble method can be used for explorative purposes and are less dependent on collective variables. However, they still require binning or a metric, which is equivalent to a collective variable. Their quantitative results do depend on the choice of phase space partition method. However, the metrics (e.g., RMSD on conformations) used in WE or MSM are typically quite obvious, making them more widely applicable for exploration than metadynamics, which usually requires the definition of a less generalist collective variable. Considering the theory behind the methods, we think that WE is generally better than MSM and could be used instead of MSM in almost all cases. However, parallel tempering is probably the method of choice for pure exploration without the need to define collective variables. We note again that scaled MD is ultimately the single-trajectory degenerate version of parallel tempering.

If exploration is not necessary and the reaction coordinate evolution is well-known, umbrella sampling can be used to estimate a potential of mean force. Umbrella sampling has some drawbacks compared to metadynamics. For example, one must manually select the centers a priori and enforce window overimposition to ensure proper reconstruction (if WHAM^110^ is used to reconstruct the profile, other methods are less dependent^112^). Umbrella sampling is more amenable for relatively simple coordinates, such as distances or better absolute coordinates. Indeed, to rigorously reconstruct the potential of mean force in umbrella sampling simulations, one must take into account Jacobian corrections that depend on collective variables. This is not required for metadynamics.

Steered MD and adiabatic bias molecular dynamics (ABMD) are excellent for exploring phase space when a target collective variable value is foreseeable. For instance, if the unbound state of the ligand is desired starting from the crystral structure, both steered MD and ABMD are powerful methods. However, ABMD is preferable because it can still navigate the phase space but is much gentler than steered MD, thus providing much more physically plausible trajectories. In theory, steered MD could be used (several replicas are needed) to obtain the free energy profile. In our experience, this nonequilibrium approach is usually less efficient than metadynamics, so we do not suggest using steered MD in this way.

Switching to the issue of computing the binding free energy, the double decoupling method^120^ is the ideal and rigorous choice for absolute free energy computations because it does not involve a collective variable. However, care must be used in dealing with restraints^120^ and significant computing time can be required. The stability of the host is also important for this method’s success.^173^

Metadynamics may also be used for computing the absolute binding free energy. Metadynamics (or umbrella sampling) for this kind of computation poses some nontrivial problems. Supposing a time-stable potential of mean force is obtained from a metadynamics simulation, it is not obvious how to move from this quantity to the binding free energy. One could consider the free energy difference between the bound and unbound states and declare this quantity as the binding free energy. However, this is not rigorous and will generally not provide a result that is directly comparable with an experiment. The rigorous approach is to compute the partition functions for the unbound and bound states. This requires the PMF to be partitioned into two regions, unbound and bound. This partition is potentially ambiguous and can significantly influence the final result. The final binding energy is thus influenced not only by a possibly poor convergence but also by a possibly user-dependent choice of the partition of unbound and bound states. Nevertheless, one should consider that different runs of metadynamics could lead to different potential of mean force curves due to poor convergence, further complicating the scenario. This poor convergence is often due to the problem’s inherent complexity and not metadynamics per se, which has been shown to converge theoretically. It is still not trivial to use metadynamics to compute the free energy of binding from a potential of mean force. To be widely applicable, a sufficiently reliable protocol must still be defined. Since one must also choose a collective variable, the approach requires a high number of possibly uncontrollable degrees of freedom, making it difficult to use. The adaptive biasing force method shares these practical difficulties.

For relative free energy estimations, we advise using methods that do not require collective variables. As explained in the Applications section, FEP and TI are now mature enough to be systematically applied in drug discovery problems and are probably now the most physics-based methods used by industry. The big first limitation of FEP/TI is that the methodology can have serious convergence problems if the perturbation is too consistent. A scaffold-hopping perturbation, for example, can become very complex. The second big limitation is that additional care is required when the perturbation involves a net charge change.

To calculate the potential of mean force or free energy surfaces in general, end-state methods are completely unusable by definition. Adaptive biasing force and metadynamics are the best solution for this task. Even if convergence is not reached due to the problem’s complexity, these methods can provide important qualitative information such as the ligand intermediate binding stations during the binding/unbinding process. This information is not yet considered sufficiently when designing ligands and it is a challenge for drug discovery endeavors.

For absolute kinetics estimation, MSM and WE are powerful tools in that they directly estimate rates rather than trying to directly rebuild the free energy surface. WE is particularly convenient due to its efficiency and the absence of any particular hypothesis. Concerning the ranking based on unbinding kinetics rates, there are methods that are much easier and faster than WE or MSM, namely scaled MD and τ-RAMD. Industrial applications of scaled MD already exist.^37^ The scaled MD algorithm is generally applicable. In addition, in many cases of interest, *k*_on_ in a congeneric ligand series is almost constant and the obtained *k*_off_-based ranking will likely correlate well with *K*_D_ and thus with the free energy. In other words, in systems where the entry barrier is often absent (e.g., kinases), scaled MD ranking is a good alternative to FEP and, because scaled MD is not a perturbative method, it does not have FEP’s limitation of tiny perturbations and charge variations.

## 8 Conclusions and Perspectives

In recent years, computational biophysics and biochemistry have developed remarkable models and simulation algorithms. Combined with the equally remarkable growth in computer facilities, these models and algorithms are beginning to impact drug discovery and development. This impact is likely to grow for the foreseeable future, with computational methods and simulation providing an important complement to experimental and clinical approaches in this challenging field.

This review first provided a brief overview of the theoretical and computational foundations of these developments. The discussion focused on mechanistic approaches that seek to reproduce the equilibrium and kinetic properties of biomolecules and drug-like compounds at the microscopic scale and, in most cases, following the real-time dynamics of the system. In other words, our discussion focused on simulation, based on atomistic or lightly coarse-grained models. For simplicity, we limited our discussion to methods that have already been used in applications. However, the literature reports several methods that we believe could be used more systematically. These include transition path sampling methods^93,101^ and confinement methods for free energy computations.^304−307^

The second central section of this paper reviewed key computational studies in drug discovery since 2010, focusing on physics-based approaches. This section was organized by method, discussing a few paradigmatic studies and reporting a short list of the most recent papers.

At present, simulation studies generally aim to predict and explain how small organic or biological species affect biological targets such as proteins, nucleic acids, and lipids. Simulation studies isolate these aspects from the more comprehensive considerations of systems biology and the clinic. Simulation is likely to become more complex and inclusive of chemical and physiological aspects, approaching but not replacing experiments and other theoretical or computer-based methods operating at higher levels of abstraction. We expect simulation to continue to provide a detailed high-resolution description of drug-target properties and phenomena, helping experimentalists and clinicians to understand the mechanisms behind the observed effects and to identify structural improvements for drugs at the molecular level.

This task is challenging due to the complexity of biomolecular systems, which is reflected in the difficulty in sampling their phase space and in the many time scales to be covered. This review therefore considered enhanced sampling methods and rare event approaches. Both fields have experienced rapid development. Just over a decade ago, many tasks were considered far beyond the reach of simulation. These tasks include folding a small protein or estimating the reaction kinetics of ligand-protein complexes. They are now becoming feasible. Moreover, the discussed algorithms have played a greater role in these developments than the increased computational power. This is important because it suggests that technology issues will not limit the future rate of development.

Methods for drug discovery must be rigorous, based on state-of-the-art knowledge, yet still deliver results in a way that is affordable and fast enough for industrial drug discovery. These methods often feature relatively short computing times and a reduced number of degrees of freedom, which are determined by the user. These aspects are critical for the systematic real-world application of any algorithm to any problem. Interestingly, FEP was not feasible for a drug discovery campaign when it was designed, but today it is a gold standard. It is likely that, in the next 20 years, this path will be followed by several methods that currently require a significant sampling effort. To ensure that these future winners survive the development stage, the drug discovery community must be aware of a broad variety of concepts and methods. Needless to say, improvements in methods and in sampling capability must go hand in hand with the improved efficiency and reliability of basic modeling and force fields especially. In general and in the very long-term, plain MD with a management of the trajectories (such as the weighted ensemble) could be a highly promising strategy. This is because it couples unbiased potentials with proper strategies for sampling rare events. Adaptivity in the sampling process cannot be overlooked in practice if one wishes to obtain an efficient sampling machinery. Another interesting aspect is that it is already effective to use simulation kinetic methods to rank compounds. These methods can be used in drug discovery campaigns today, although broader validation is needed.

Critically, simulation methods are rarely prospective. Methods are mostly applied retrospectively without further validation. This situation requires standardized benchmarks, blind competitions, common force fields, shared code bases, and data repositories. It is encouraging that some sections of the community are working hard in this direction.^181,308−311^

We identify several major challenges for the community and for the methods in achieving the maturity required for the systematic application of these approaches in drug discovery. The first big challenge is cultural and it requires standardization. The computational drug discovery community that deals with free energy and kinetics methods cannot seriously proceed in testing and validating algorithms without a widely accepted benchmark reference. As anticipated, the situation is already changing and host–guest systems are very well suited for this aim. Comparing methods using these systems is essential to understanding the features of each method in depth. The community has run algorithms alone on a specific system without comparison in many cases. There are many reasons for this, including the difficulty of mastering several methods, a bias in promoting a specific methodology, and the ever-present limitations on computational power. That is why we need more automation in methods, less user intervention, and more computational power. It is the only way to run rigorous comparative assessments. This goes back to the already discussed need for almost black box methods or, at least, default values for parameters and choices. If this is not possible, then unbiased comparisons become difficult. This demonstration of maturity will be challenging for the community to achieve. One example of the lack of standardization in this field is that there is still no accepted standard for naming residues. For example, a double-protonated histidine can still be named HIS, HIP, and HI+. Although not practically disabling, this issue should sound the alarm that standardization is needed at various levels.

The second big challenge is computational power. GPUs have started a revolution in this field, but we are just at the beginning. The fact that we can run *one* simulation does not mean that we have enough computational power. On the contrary, to really assess errors and poor convergence, we should run the same simulation several times and accumulate statistics. Given the complexity of the Boltzmann distribution for molecular systems, the available computational power is not yet sufficient to work as robustly as we would like (in the time frame we would like). Interestingly, non-Turing classical attempts are an emerging field per se. Recent results^312,313^ of quantum simulations on quantum Turing machines are a fascinating and promising path, although error management and scalability in general are nontrivial problems that must still be addressed.

The third big challenge is force fields. While there are already excellent force fields (we have cited several of them), the divergence of results using difference force fields is troubling. Force fields should ultimately converge to a common open gold standard. Consortia could be the solution and we look with extreme optimism to this path.^310^

Overall, the enhanced sampling community has created splendid theories in recent years, but it needs to develop the proper tools, good practices, and language from engineering to move forward from what are currently somewhat fragmentary research experiences. This is a huge effort but it is necessary to take excellent theory and apply it to real-world problems. This is not just a matter of implementation. Rather, it may require that we revisit theoretical aspects that can differ between simple model systems and complicated target complexes. This need has already been recognized by a large section of the community, which is already working to significantly increase the technological readiness of the available methodologies.
